# Advanced Nanomedicines for Treating Refractory Inflammation-Related Diseases

**DOI:** 10.1007/s40820-025-01829-7

**Published:** 2025-07-07

**Authors:** Xiuxiu Wang, Xinran Song, Wei Feng, Meiqi Chang, Jishun Yang, Yu Chen

**Affiliations:** 1 Naval Medical Center of PLA, Naval Medical University, Shanghai, 200052 People’s Republic of China; 2 Materdicine Lab, School of Life Science, Shanghai University, Shanghai, 200444 People’s Republic of China; 3 Laboratory Center, Shanghai Municipal Hospital of Traditional Chinese Medicine, Shanghai University of Traditional Chinese Medicine, Shanghai, 200071 People’s Republic of China; 4 Shanghai Institute of Materdicine, Shanghai, 200051 People’s Republic of China

**Keywords:** Nanomedicine, ROS scavenging, Nanoparticles, Nanozymes, Pancatalysis

## Abstract

This review examines inflammation as a physiological defense mechanism against infectious agents, physical trauma, reactive oxygen species (ROS), and metabolic stress, which, under dysregulated conditions, may progress into chronic diseases. Nanomedicine, which integrates nanotechnology with medicine, suppresses inflammatory signaling pathways and overexpressed pro-inflammatory cytokines, such as ROS, to address inflammation-related pathologies. Current advances in nanomaterial design and synthesis strategies are systematically analyzed, with parallel discussions on toxicity mechanisms, influencing factors, and evaluation methods that are critical for clinical translation. Applications of functional nanomaterials are highlighted in the context of refractory inflammatory conditions, including wound healing, gastrointestinal disorders, and immune, neurological, or circulatory diseases, along with targeted delivery strategies. Persistent challenges in nanomedicine development, such as biocompatibility optimization, precise biodistribution control, and standardized toxicity assessment, are critically assessed. By bridging material innovation with therapeutic efficacy, this review establishes a framework for advancing nanomedicine to improve treatment outcomes while addressing translational barriers.

## Highlights

- An overview of inflammation related diseases has been provided.
- The classification of nanomaterials commonly utilized in the treatment of various inflammatory diseases has been outlined.
- The current state of nanomedical applications with desirable therapeutic efficacy in the treatment of inflammatory diseases has been sum marized.
- The challenges and perspectives in the evolving field of nanomedicine for treating inflammatory diseases have been discussed and proposed in depth.

## Graphical abstract

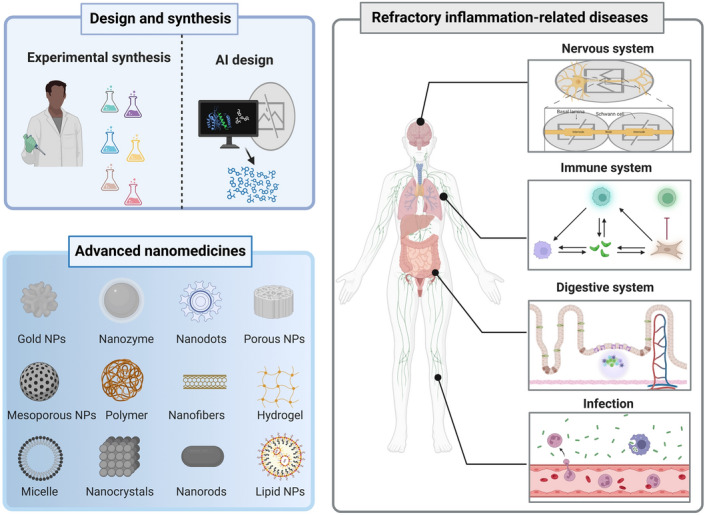

## Introduction

Nanomedicine is an emerging discipline that integrates nanotechnology into medicine, enabling nanomaterials to play a pivotal role in the diagnosis and treatment of diseases, thus offering new therapeutic options for a wide range of common health conditions-often referred to as nanotherapies [1]. In contrast to conventional drugs, nanomedicines have nanoscale dimensions that offer a larger specific surface area, facilitating easier surface functionalization. Additionally, these therapeutics exhibit unique physicochemical properties, such as low toxicity, high bioavailability, and improved pharmacokinetics, often leading to enhanced therapeutic effects [2–4]. In recent years, research in nanomedicine has gained significant momentum, fueled by advancements in research technologies. This progress has led to a substantial increase in the diversity of nanomaterials and their applications. Notably, there has been a growing use of nanomaterials in the diagnosis and treatment of inflammatory diseases [5].

Inflammatory diseases pose significant challenges and draw considerable attention in the medical field, as inflammation is linked to nearly all human diseases [5, 6]. An appropriate inflammatory response is a crucial defense mechanism, triggering tissue repair through immune modulation in response to pathogen invasion or tissue damage [7]. Persistent inflammation evolves into a pathological response, leading to uncontrolled damage to the organism. Additionally, tissue or organ necrosis can trigger further inflammation, creating a vicious cycle that exacerbates the condition [8]. When tissues are exposed to exogenous or endogenous stimuli, inflammatory mediators and cytokines are released by inflammatory cells. These mediators act on effector cells, including immune cells (monocytes, macrophages, and neutrophils) and non-immune cells (endothelial and smooth muscle cells). This can lead to cellular phenotype polarization or overexpression of relevant proteins, triggering a cascade of biological processes that may result in tissue and organ damage [9]. Moreover, an inflammatory response that is not properly controlled can persist, leading to chronic inflammation and refractory inflammatory diseases. This causes long-term damage to the organism and increases treatment costs, presenting an urgent biomedical challenge.

Among the various inflammatory mediators, ROS have garnered significant attention for their role in the inflammatory response, including superoxide anion (O_2_·^−^), hydrogen peroxide (H_2_O_2_), hydroxyl radical (•OH), and singlet oxygen (^1^O_2_) [10]. Under normal conditions, ROS serve as key signaling molecules in metabolic processes and play a crucial role in various physiological functions, such as the oxidation of proteins, lipids, and polynucleotides, all of which are essential for maintaining physiological homeostasis [11]. Excess ROS are considered toxic by-products that can activate inflammatory responses. Oxidative stress induced by excess ROS not only damages biomolecules such as DNA, proteins, and lipids [12], but also directly leads to cell death [13]. Additionally, oxidative stress can contribute to the development of various conditions, with inflammation being the most prevalent. Beyond promoting inflammation, ROS can cause mitochondrial damage, disrupt cell membranes, and trigger apoptosis (leading to neurological damage), affect Ca^2+^ homeostasis, and activate related channels or receptors, such as TRPC3 and TRPC4 channels and ryanodine receptors. This can contribute to the development of cardiovascular disorders (e.g., myocardial infarction, arrhythmia, and atherosclerosis) and neurodegenerative diseases (e.g., Parkinson’s disease and Alzheimer’s disease) [14–18]. Thus, ROS exhibit a dual nature, with their impact being dependent on their concentrations, suggesting that the toxicity of ROS can be controlled. While ROS production can have beneficial effects, such as killing bacteria, aiding infections, and promoting wound healing, it can also be harmful. The removal of excess ROS can reduce toxicity, minimize bodily damage, alleviate symptoms, and slow the progression of disease. Given the complexity of inflammatory signaling pathways, managing inflammation-related diseases requires more than just targeting oxidative stress. The mechanism of action of nanomaterials extends beyond ROS regulation, taking a broader approach. Current treatments heavily rely on hormonal drugs, which lack diverse therapeutic mechanisms. In contrast, nanomedicine offers a comprehensive solution, with nanomaterials targeting ROS as well as other mechanisms. Furthermore, poor adherence to traditional treatments is well documented, and the precision-targeted, highly effective features of nanomedicine could address this issue.

This has led to the development of various anti-inflammatory and antioxidant nanomaterials. For instance, nanozymes utilize their catalytic properties to generate or eliminate ROS [19], while certain metallic nanoparticles (NPs) exhibit similar properties and can function as drug carriers, thereby enhancing therapeutic efficacy [18]. Additionally, exosomes can transport natural medications or genes for palliative purposes [19]. Although these nanomaterials have shown remarkable efficacy in in vitro tests, replicating these effects in complex physiological environments remains challenging. Employing effective targeting strategies, such as passive and active targeting, will enable precision therapy and enhance the efficacy of nanomedicines [20]. Optimizing the design of nanomaterials is vital for ensuring their therapeutic efficacy, while employing suitable synthesis methods is essential for minimizing toxicity and enhancing efficiency. Despite the diversity and widespread use of nanomaterials, along with their superior efficacy over traditional therapies in treating various diseases, the toxicity of certain nanomaterials continues to be a major obstacle to their clinical translation. Consequently, the toxicological study of nanomedicines has garnered increasing attention, with the goal of providing a theoretical foundation for designing safe and effective nanomaterials, focusing on mechanisms and influencing factors.

**Scheme 1:**
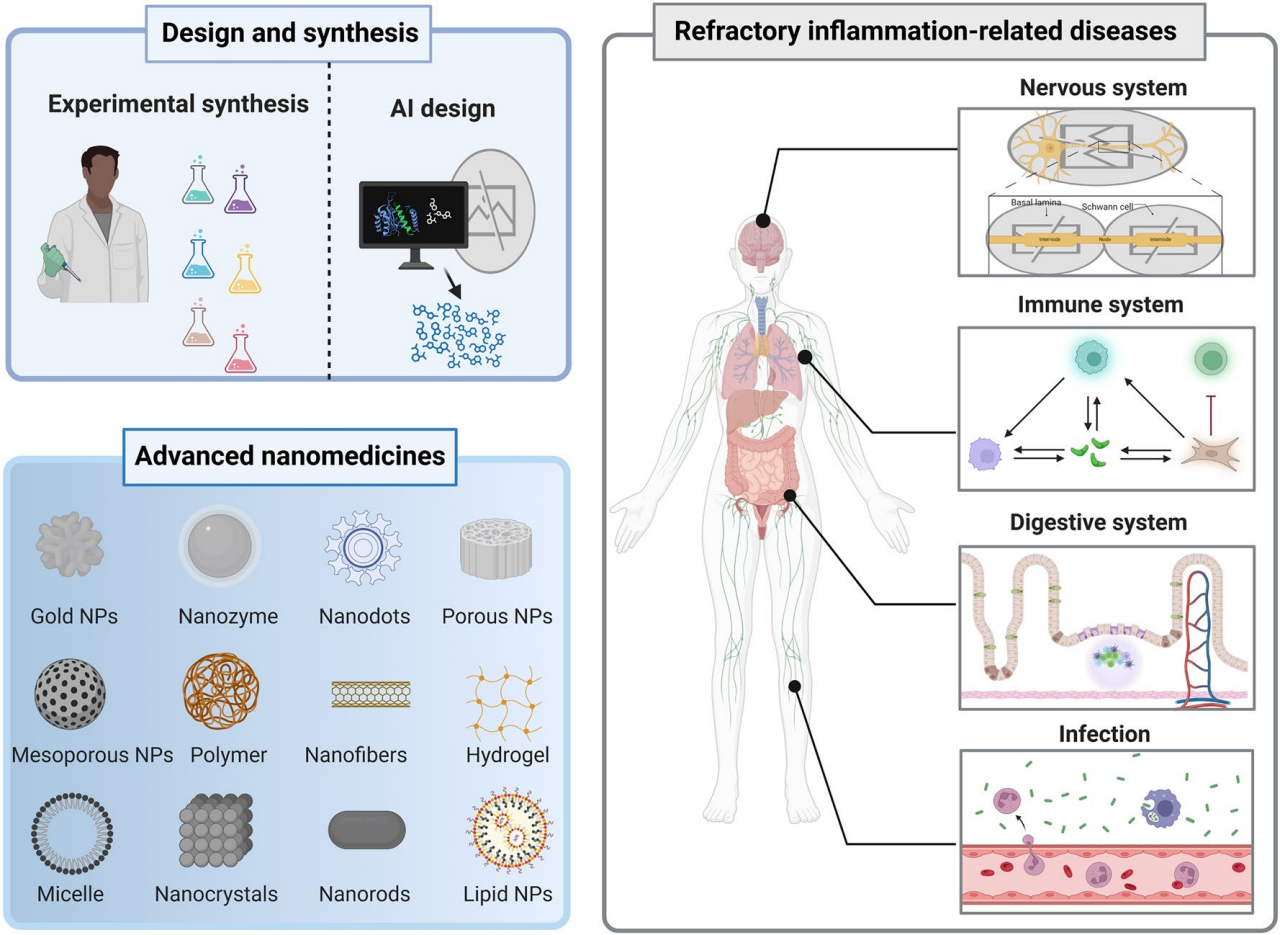
Schematic illustration of therapeutic nanomedicine. This includes various nanomaterials, such as NPs, liposomes, nanozymes, nanofibers, nanocrystals, micelles, and nanorods, intended for the treatment of inflammation-related diseases. Created with BioRender.com

In this review, we present a comprehensive summary of the design and synthesis strategies for nanomaterials, with a particular emphasis on the application of advanced “smart” methods. We then provide a concise overview of nanomaterial toxicity, covering mechanisms, influencing factors, and evaluation approaches that will serve as a foundation for toxicological studies facilitating the clinical translation of nanomedicines. Additionally, we highlight the use of diverse functional nanomaterials in treating refractory inflammation-related diseases, including wound healing, digestive disorders, immune conditions, neurological diseases, and circulatory disorders. The various strategies for enabling targeted therapies for these diseases through nanomedicine have also been explored. Finally, the current barriers to the application of nanomedicines in treating inflammation-related diseases are discussed (Scheme 1). This review offers valuable insights into the types of nanomaterials and their role in treating inflammation-related diseases, contributing to the development of innovative nanomedicines aimed at enhancing disease treatment efficacy.

## Design and Synthesis of Nanomaterials

The design and synthesis of nanomaterials are fundamental to their physicochemical properties and biological effects. As research progresses, there is a growing trend toward the development of intelligent nanomaterials, such as stimuli-responsive and biomimetic types, which enhance the precision and efficiency of nanomedicine for more targeted treatments. Furthermore, with advancements in intelligent technology, emerging fields like artificial intelligence (AI) are increasingly being integrated into nanomedicine. AI applications, such as machine simulations and high-throughput screening (HTS), provide more accurate and efficient methods for designing safe and effective nanomaterials. In the synthesis of nanomaterials, the adoption of appropriate techniques can reduce reliance on chemicals and extreme conditions, thereby facilitating the safe, energy-efficient, and controlled mass production of nanomaterials. Additionally, intelligent approaches like biosynthesis, biomimetic self-assembly, and biomimetic mineralization offer precise control over the structure, properties, and functions of nanomaterials, facilitating their autonomous and responsive synthesis.

### Smart Design of Nanomaterials

**Scheme 2:**
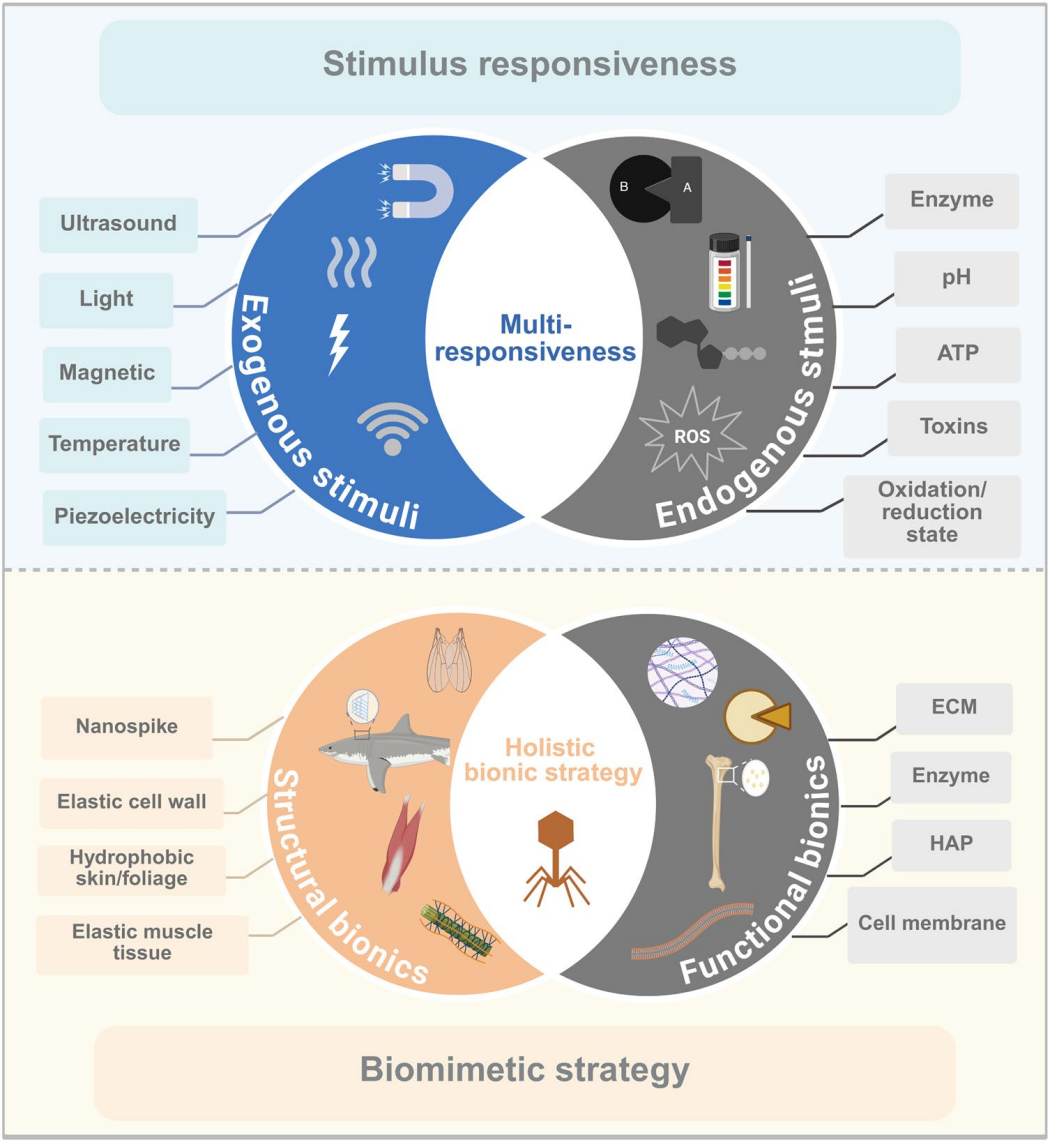
Schematic diagram of the design scheme for smart nanomaterials. This design strategy is primarily categorized into two approaches: stimulus-responsive and biomimetic strategies. Created with BioRender.com

Unlike conventional drug molecules, nanomedicines possess distinct nanoscale structures. Through rational and advanced structural design, these nanomaterials can demonstrate enhanced properties, including improved mechanical strength, catalytic efficiency, and biological activities such as anti-inflammatory effects. In recent years, “intelligent” nanomaterials have been developed to respond to physiological stimuli in the body, or to specifically target and interact with disease-causing genes or proteins. This enables more precise and efficient intervention, curbing disease progression and promoting the restoration of health. Furthermore, multitargeting and hierarchical targeting strategies enhance the capacity of nanomedicines to overcome in vivo obstacles, ensuring their delivery to the intended target site for effective treatment [21]. Therefore, the “intelligence” of nanomaterials significantly enhances their performance in the field of medicine. The current design of smart nanomaterials is principally categorized into two types: stimuli-responsive nanomaterials, which facilitate controlled drug release at targeted sites, and biomimetic nanomaterials, which are functionalized through biomimetic strategies to improve the targeting of pathological sites by nanomedicines (Scheme 2). Additionally, to improve the practicality of nanomaterials, AI, with its advanced statistical and analytical capabilities, is increasingly being used in their intelligent design. This approach offers an efficient strategy, focusing on biological principles from the outset and reducing the costs associated with trial and error [22].

#### Stimuli-Responsive Nanomaterials

Nanomaterials with specialized structures and functions are often used as carriers for therapeutic substances. These materials not only provide a functional delivery platform for active compounds but also improve the physicochemical properties of the drugs, such as solubility and in vivo stability. Additionally, they enhance the on-demand release or specific accumulation of active ingredients in targeted regions. In stimulus-responsive nanosystems, the “smart response” capability arises from the specific interaction of nanomaterials or their functional groups with particular in vivo environments. For example, boronic acids are multifunctional groups that can selectively react with substances such as ROS, ATP, and glucose [23–25]. Stimulus-responsive nanocarriers can sense both exogenous and endogenous stimuli, responding sensitively with actions such as cleavage, content release, and aggregation. Exogenous stimuli, including physical factors like magnetic fields, temperature, light, ultrasound (US), and piezoelectricity, are applied externally to trigger the release of active ingredients from the nanocarriers under altered environmental conditions. Among these, US-responsive nanomaterials are capable of mediating sonodynamic therapy (SDT), which involves the efficient generation of ROS to effectively kill cancer cells and bacteria when combined with low-intensity US and sonosensitizers [26, 27]. Our team has developed an oxygen vacancy-rich MoOx@Mo_2_C nanoagent that efficiently captures bacteria through its tightly packed mesh structure. It rapidly generates a significant amount of ROS with a broad-spectrum bactericidal effect. This is achieved through the rapid separation of electron-hole pairs, coupled with oxygen vacancy reduction and electron-hole pair recombination, in the presence of US [28].

Over the last decade, research on stimuli-responsive nanomaterials has entered a new phase driven by continuous innovations in functionalized nanotechnology. Traditional systems, such as magnetic iron NPs [29], thermosensitive nanogels [30], and photosensitive metal–polymer NPs [31], were previously designed to respond to singular external physical stimuli. However, the latest advancements involve integrating multiple response mechanisms and therapeutic approaches to achieve higher-performance nanomaterials. For example, light is an exogenous stimulus that can be flexibly applied. Nanomaterials with photosensitive or photothermal properties can undergo photochemical reactions or photothermal transformations, respectively, to destroy harmful cells such as bacteria and cancer cells. This is achieved through photodynamic therapy (PDT) and photothermal therapy (PTT) [32, 33]. Our team developed oxygen-deficient TiO_2-x_ nanocrystals (B-TiO_2-x_) for synergistic PTT/SDT in cancer treatment. The oxygen-deficient layer enhances US-induced electron–hole separation, improving SDT efficiency. Meanwhile, the core–shell structure enhances photothermal conversion, boosting PTT effectiveness. This dual-action nanoplatform exemplifies the synergy between SDT and PTT, broadening the potential applications of stimulus-responsive nanomaterials in precision oncology [34]. Piezoelectric nanomaterials generate electrical currents in response to mechanical forces and induce a mechanical response in an electric field [35]. Our team developed piezoelectric 2D MoS_2_ nanosheets that, under US, convert mechanical vibrations into electrical energy, generating ROS to kill cancer cells. Additionally, the photothermal properties enable photoacoustic imaging, thermography, and PTT. The synergy between piezoelectric and photothermal effects further enhances their anticancer efficacy [36].

Exogenous nanoplatforms depend on external stimuli, which can introduce complexity and imprecision due to uncontrolled inputs. In contrast, endogenous nanomaterials autonomously sense disease-specific microenvironments, enabling precise drug release and aggregation without the need for external intervention. Research on bioresponsive nanomaterials, targeting enzymatic factors, pH, toxins, ATP, and redox states in disease microenvironments, focuses on catalytic and redox reactions as well as bond cleavage to enable precision drug release. Redox-responsive designs are notably effective, leveraging elevated glutathione (GSH) levels to cleave disulfide (-S-S-) bonds in nanocomposites. Similarly, diselenide (Se-Se) and carbon–selenium (C-Se) bonds demonstrate heightened GSH sensitivity, enabling controlled therapeutic delivery. These materials intelligently respond to pathological conditions through endogenous triggers, optimizing targeted antibiotic release. Oxidation-responsive functional groups include boron esters [37] and tetrasulfopentene [38], among others. In particular, these materials exhibit distinct behaviors in oxidizing and reducing environments, undergoing cleavage or aggregation, respectively. This renders them multifunctional groups with significant roles in nanomedicine synthesis and targeting. Our team developed GSH-responsive TGA-Cu NPs, where TGA-GSH exchange disrupts tumor proteins, while GSSG-Cu triggers Fenton-like reactions with H_2_O_2_ to generate cytotoxic •OH, enabling dual-action anticancer therapy [39]. Additionally, the integration of both exogenous and endogenous stimulus-responsive strategies is a common design approach for smart nanomaterials. This often involves a combination of multiple responsive forms, such as pH/US response or magnetic/pH/temperature responsiveness. We designed dual pH-/near-infrared (NIR)-responsive DOX@silicene–bovine serum albumin (BSA) nanosheets to enhance chemotherapy and PTT. Acidic pH conditions protonate DOX, increasing its hydrophilicity and promoting drug release. Silicene’s photothermal properties enhance NIR sensitivity, and the heat generated accelerates DOX release in a synergistic manner, improving the targeted tumor treatment efficacy [40]. Similarly, due to their high photothermal conversion efficiency, Ti_3_C_2_ MXenes have been shown to effectively eradicate tumors through the synergistic combination of PTT and chemotherapy [41]. Qiu et al*.* developed multistimuli-responsive V-HAGC NPs, hollow mesoporous CuS-based nanodrugs targeting fibroblast-like synovial cells for precision therapy in rheumatoid arthritis (RA). In the pathological environment of RA, hyaluronic acid (HA) (ROS-responsive) first undergoes cleavage, followed by NIR light and acidic pH-triggered decomposition, which releases GOx and atovaquone. This hierarchical, spatiotemporally controlled drug delivery system enables highly effective treatment of RA [42]. Additionally, our team documented the pH/US-responsive release of ropivacaine from hollow mesoporous organosilicon NPs (HMONs), enabling the induction of prolonged analgesic effects [43]. Cardoso et al*.* developed triple-responsive (thermo/magneto/pH) magnetic liposomes for the controlled delivery of anticancer drugs [44].

Bioorthogonal chemistry, which enables efficient reactions under physiological conditions, is integrated with advancements in nanotechnology to enhance biomedical applications such as in situ drug activation, targeted delivery, bioimaging, and biosensing, thereby enabling precise therapeutics. Tetrazine-based bioorthogonal chemistry, leveraging ultrafast kinetics and high selectivity, drives these innovations through interactions between tetrazine and target reagents [45]. Wu et al*.* developed a ROS-responsive prodrug, TCO-NB-GABA, by linking nitrobenzyl, 4-TCO, and GABA. Exploiting the excess ROS in epilepsy, tetrazine precursors formed hydrogelators that reacted with TCO-NB-GABA via bioorthogonal chemistry, releasing GABA at lesion sites [46]. In essence, the click reaction between tetrazine and 4-TCO enables targeted drug release. This strategy, which responds to specific environmental cues or components, is categorized as a stimuli-responsive targeting approach.

Host-guest interaction-driven, stimuli-responsive materials integrate supramolecular chemistry with advanced material design. Macrocyclic hosts (e.g., cyclodextrins, cucurbiturils) dynamically bind hydrophobic or hydrogen-bonded guests (e.g., adamantane, azobenzene). These complexes dissociate reversibly in response to environmental triggers (e.g., pH, temperature, enzymes, ATP), allowing precise control over material responses for applications such as targeted drug delivery and adaptive systems [47]. Ni et al*.* developed a photo- and temperature-responsive antibacterial surface using azobenzene-cyclodextrin (Azo/CD) host–guest interactions. A hydrophilic polyHEMA layer prevents bacterial adhesion, while polyNIPAM’s conformational changes, in synergy with Azo/CD dissociation under UV and heat, enhance bactericidal performance and recyclability. Specifically, UV light triggers Azo/CD dissociation, while visible light restores binding, enabling reversible regeneration for sustainable antibacterial applications [48].

Molecular recognition-driven responsive designs leverage biomolecular interactions (e.g., ATP, miRNA) to dynamically regulate material functions in response to external stimuli. Unlike static ligand modifications, these systems use aptamers (synthetic ssDNA/RNA) that competitively dissociate from cDNA when target molecules with higher binding affinity replace them. This release exposes active sites (e.g., drug channels), enabling stimuli-triggered drug delivery. Binding energy differences drive the separation of the aptamer-cDNA duplex, providing precise control through bioorthogonal molecular competition [49]. For example, Esawi et al*.* developed a chimeric complex consisting of two aptamers to deliver doxorubicin to cancer cells: the AS1411 antinucleolin aptamer for targeting cancer cells and the ATP aptamer for drug loading and triggered release [50].

#### Biomimetic Nanomaterials

The smart materials being developed are multifunctional and dynamically adjustable, capable of navigating complex pathological environments and optimizing therapeutic treatment delivery. Biomimetic design, inspired by nature, brings to life a range of possibilities for smart materials and serves as a powerful approach to enhancing the intelligence of nanomaterials. In recent years, research in biomimetics has progressed significantly, leading to the emergence of concepts such as “biomimetic medicine” and “biomimetic interfaces” [51, 52]. Precisely, biomimetic materials replicate natural substances of interest, with varying degrees of mimicry in both structural and functional aspects, thus imparting nanomaterials with the desired properties and functions. For example, cell membranes coated with nanomaterials demonstrate multifunctionality [53]. The biological origin of the cell membranes enhances the biocompatibility of materials. Targeting is facilitated by the specific binding of membrane proteins to receptors in the target area, while immune evasion and extended circulation time are promoted through the activity of membrane proteins. Thus, this biomimetic design exemplifies functional biosynthesis [54], a purpose-driven approach that involves selecting natural substances with specific functions and replicating or leveraging their structures to mimic particular biological functions. Another biomimetic approach focuses on replicating the synthesis processes of natural substances, known as process biosynthesis [54], which will be discussed in the following section.

*Structural biomimicry*. This overview highlights several advanced biomimetic design methods from the perspective of natural objects mimicked by nanomaterials. These methods include the simulation of macroscopic structures such as plants and animals, as well as microscopic entities like viruses, biomolecules, and extracellular vesicles (EVs), thereby broadening the scope of nanomedicine applications. The surfaces of many organisms in nature exhibit unique structures and properties, which contribute to specific biological activities. The term “biomimetic interface” refers to the process of replicating and fabricating surface nanostructures based on these distinctive morphologies or properties, offering valuable biological insights for the design of functionalized nanomaterials. For example, surface nanopillar array structures are a well-known instance of physical bactericidal surfaces. Tian et al*.* fabricated cicada wing-inspired silicon nanosheets with 600-nm-high, 50-nm-base nanospikes using reactive ion etching, achieving a 60% *Escherichia coli* (*E. coli*) elimination within 30 h, demonstrating potent antibacterial activity [55]. Studies show that the surface protrusion column density and height of nanomaterials critically influence antimicrobial efficacy by modulating bacterial adhesion. Peak bactericidal efficiency is achieved at an optimal surface roughness, which is determined by high column density and height. At this optimal density, bacterial membranes adhere perpendicularly to the tips of the spikes and their adjacent surfaces, causing membrane stretching and deformation. Variations in spike height further amplify roughness, increasing membrane stress and structural damage. This dual mechanism—geometric adhesion forces and mechanical disruption—enhances the physical destruction of bacteria, thereby improving antimicrobial performance [56].

Additionally, physical properties such as adhesion, wettability, and mechanical characteristics of the surface interface play crucial roles in determining the antimicrobial activity of nanomaterials. For instance, the diamond-like corrugations on shark skin minimize water resistance while providing antifouling properties. Enhanced by elastic, stress-resistant mechanical traits, this biomimetic approach inhibits bacterial adhesion and enhances bactericidal efficacy through an optimized synergy of structure and material. Arisoy et al*.* mimicked the antimicrobial properties of TiO_2_ NPs by creating a fouling-resistant shark skin surface using solvent-assisted nanoimprint lithography, yielding a smooth biomimetic interface that significantly diminishes microbial attachment [57]. In addition, Valiei et al*.* highlighted an intrinsic relationship between a material’s surface wettability and its antimicrobial efficacy. The total capillary force, acting as the external driving force responsible for bacterial deformation, is weakest on hydrophobic surfaces and strongest on hydrophilic ones. Consequently, the bactericidal activity of superhydrophilic surfaces reaches its peak and diminishes as surface hydrophobicity increases. However, superhydrophobic surfaces effectively reduce bacterial adhesion and provide excellent self-cleaning properties, along with antimicrobial benefits [58]. Chen et al*.* developed fruit leaf-inspired nanoflakes with superhydrophobic surfaces and randomized serrated edges that physically rupture surface-adhered bacteria. This dual structural and functional biomimicry synergizes antifouling properties and mechanical sterilization, enhancing antimicrobial efficacy [59].

Nanomaterials possess exceptional mechanical properties. For instance, carbon nanotubes (CNTs) are made of carbon atoms arranged in *sp*^2^ hybridized covalent bonds, with a higher proportion of s-orbitals contributing to their remarkable mechanical characteristics. This unique structure provides CNTs with high modulus, tensile strength, and elongation at break, making them highly resistant to deformation and capable of withstanding substantial stress. However, many current nanomaterials struggle to replicate the elastomechanical behavior of natural organisms. Inspired by nature, superior elastic properties can be achieved by mimicking the structure of animal muscle tissue and plant cell walls. Du et al*.* used poly(lactic-co-glycolic acid)–polyethylene glycol (PLGA-PEG) to replicate the highly elastic structure of natural skeletal muscle tissue, which also promotes myoblast differentiation and tissue formation [60]. Plant cell walls, with their microfibril-reinforced polymer matrices, provide significant mechanical strength to resist environmental stresses, inspiring the development of biomimetic nanomaterials. Shi et al*.* replicated this structure by engineering a bionic cell wall (BCW) on animal cells, utilizing supramolecular DNA templates to guide the assembly of an extracellular polysaccharide–peptide matrix. The BCW protected mammalian cells from adverse conditions, enhancing their viability and illustrating how plant-inspired protective layers can be synthetically replicated to strengthen cells. This breakthrough opens new avenues for synthetic biology and the design of stress-resistant nanomaterials [61].

*Structural and functional biomimicry*. Bioactive nanomaterials mimic the functional components of living organisms, actively recruiting progenitor cells to enhance adhesion and differentiation for tissue engineering. Hydroxyapatite (HAP), a key component of vertebrate calcified tissues, serves as a prime example, offering biocompatibility, osteogenic bioactivity, and strong cell adhesion. Its osteoconductivity and controlled biodegradability work synergistically to optimize bone regeneration [62]. Furthermore, bionic HAP creates an ideal microenvironment that promotes osteogenic differentiation in cells [63]. Jeffrey et al*.* combined HAP/collagen scaffolds with PRP, which serves as a cell-recruiting matrix, releasing chemokines and bioactivators to attract MSCs and fibroblasts, thereby stimulating their proliferation [64]. The synergistic co-application of osteogenic agents and HAP creates smart biomaterials that mimic natural bone repair mechanisms. These biofunctional, biomimetic nanomaterials meet orthopedic clinical needs by integrating cell recruitment, differentiation, and structural support, thereby advancing regenerative strategies.

*Functional biomimicry*. The extracellular matrix (ECM), composed of collagen-based filamentous networks and signaling molecules, regulates cellular adhesion, growth, and metabolism [65]. Despite its bioactivity, natural ECM faces limitations in stability and biocompatibility [66]. Biomimetic nanomaterials that replicate the ECM’s structure and components offer enhanced durability for tissue regeneration, enabling targeted biomedical applications through functional synergy.

Heparin, a key active component of the ECM, plays crucial roles in biological processes such as growth factor binding and release, anticoagulation, immune regulation, and promoting cell migration and differentiation [67]. In recent years, heparin-inspired biomaterials, integral to ECM-mimicking materials, have shown significant potential in medical applications such as tissue repair, anticoagulation, and antiviral therapy [68, 69]. Their primary function is to replicate the sulfated structures of heparin or heparan sulfate, thereby mimicking similar biological functions. This approach overcomes the limitations of natural heparin and enhances the biomimetic properties of the materials. For instance, sulfation modifications of polymers such as chitosan (CS) and PEG replicate the negative charge characteristics of heparin, enabling the synthesis of materials like nanofibers and hydrogels [70]. Alternatively, heparin oligosaccharides, protein sequences, or heparin-like molecules can be directly incorporated into nanomaterials, enabling the design of heparin-inspired biomaterials [71, 72].

Furthermore, nanofibrous materials can replicate the fibrous architecture of the ECM, serving as biomimetic scaffolds to guide cell growth and tissue regeneration. These materials are widely used in various tissue engineering applications. For example, Wang et al*.* prepared nanofibers made of polycaprolactone (PCL), silk fibroin (SF), and CNTs via a dry-wet electrospinning method. This approach mimics the three-dimensional hierarchical structure of natural neural tissues, promoting neural synapse migration and elongation along the direction of the nanofibers, thereby aiding neural tissue repair [73]. Additionally, ECM secreted by cells can further promote the differentiation of bone marrow mesenchymal stem cells (MSCs) under specific physical stimuli. As a result, combining ECM with stimulus-responsive nanomaterials has become a common approach in bone tissue engineering. For instance, Wu et al*.* developed a biocompatible graphene–ECM nanocomposite film. The photothermal effect of graphene elevated the surface temperature under light, thermally stimulating cell growth and osteogenesis, thereby demonstrating light-triggered enhancement of bone regeneration [74]. However, each component of the ECM theoretically serves a specific function in cell growth, and mimicking a single component may not fully replicate the complex physiological environment of the ECM. Therefore, achieving a complete mimicry of the natural ECM holds greater clinical value for biomedical applications.

Cells, as fundamental biological units, serve as inspiration for bionic nanotechnology. Traditional methods modify nanomaterials with polymers or ligands to create stealth coatings that reduce immune clearance and enable targeted delivery. However, sequential ligand coupling is inefficient and raises safety concerns. Cell membrane-camouflaged biomimetic nanomaterials address these issues by incorporating natural membrane proteins that evade immune phagocytosis, prolong circulation, and facilitate cell-specific targeting through ligand-receptor interactions. This strategy harnesses the intrinsic functionality of cells to enhance nanomaterial performance, combining biological precision with engineering efficacy for advanced therapeutic applications [53, 75]. Cell membrane biomimetic coatings effectively endow nanomaterials with multifunctionality and have been extensively studied in preclinical research. Various sources-such as erythrocytes, macrophages, neutrophils, platelets, stem cells, cancer cell membranes, and vesicles (microvesicles, exosomes)-broaden the biofunctional versatility of these materials for targeted applications [76, 77]. Tan et al*.* encapsulated macrophage membranes in the outer layer of polymeric NPs (PLGA-LPV NPs) loaded with lopinavir. The resulting PLGA-LPV@M NPs demonstrated a remarkable ability to target inflammatory sites, neutralize multiple pro-inflammatory cytokines, and reduce inflammation, while also decreasing tissue viral loads [78]. Hybrid cell membrane coatings address the limitations of single-source membranes, enabling dual-targeted drug delivery. Chen et al*.* developed the nanomimetic Asp8[H40-TPZ/IR780@(RBC-H)] to achieve immune evasion and targeted therapy for oral squamous cell carcinoma [79].

Exosomes, natural cell-derived nanovesicles, retain the original cell membrane proteins and lipids [80]. Their membrane proteins facilitate intercellular communication and cell-specific targeting, while the lipid bilayer protects the cargo from enzymatic degradation and clearance [81]. As biomimetic nanocarriers, exosomes harness inherent biological functions to enable precision therapy and targeted drug delivery in nanomedicine.

Virus-mimicking nanomaterials enhance penetration, targeting, and immunogenicity by utilizing the efficient cell invasion and vaccine-like antigenicity of viruses. These strategies, employed in tumor immunotherapy and vaccine development, leverage viral traits, such as electroneutral surfaces, to enhance intestinal absorption, advancing the design of biomedical nanomaterials. Zhang et al*.* engineered electroneutral MSN-NH_2_@COOH/CPP5 by coating mesoporous silica NPs (MSNs) with the KLPVM peptide and glutaric anhydride. This nanoparticle successfully penetrated intestinal barriers, enabling efficient insulin delivery and a significant reduction in blood glucose levels in diabetic rats [82]. Additionally, nanomaterial-modified polymers, peptide chains, and nanotubes can mimic the spiky structure of viral surfaces, enhancing cellular endocytosis, increasing the bioavailability of nanomedicines, or improving the immunogenicity of nanovaccines. Gao et al*.* engineered MSNs with virus-mimetic radiating nanotube spines, replicating viral surface topology to enhance cellular uptake and endosomal escape of antigens, thereby enabling efficient immune presentation [83]. To improve the accuracy of bionanostructures, Zhao et al*.* engineered a virus-mimetic nanosystem, ZM@TD (Mn-doped ZIF-90), which mimicked viral nucleocapsids to protect DNAzyme. Erythrocyte membranes facilitated immune evasion, while RGD/HA2 peptides replicated herpesvirus glycoproteins, triggering antigen release and sustained activation of innate immunity. This design resulted in a 68% primary tumor regression through enhanced immunotherapy efficacy [84]. Therefore, adopting a holistic, systemic approach—rather than simply incorporating a single viral signature into nanomaterials—may hold greater potential for the successful clinical translation of virus-inspired nanomaterials.

#### Computational Nanomaterials

Smart nanomaterials are ideally designed for dynamic regulation and precision therapy in pathological environments. However, in practice, the fate of nanomaterials in living organisms remains unknown and challenging, leading to a significant reduction in their effectiveness and potentially causing uncertain toxic side effects. Additionally, the variety of ligands used to modify nanomedicines or adjuvants applied to nanosystems increases the cost of trial and error, as they are often selected based on literature research or subjective guesses, which contradicts the concept of “intelligent” nanomaterial design. To address this, computational technologies with powerful data processing and analysis capabilities have been increasingly applied to the intelligent design of nanomaterials. These technologies, such as high-throughput methods or machine learning (ML), can efficiently summarize a large body of existing research, elucidating the interaction patterns between nanomaterials and biological systems, as well as their connections with biomolecular ligands. This provides advanced technical support for the rational and efficient design of disease-specific intelligent nanomedicines, while also addressing the challenge of characterizing the physicochemical properties and biological effects of nanomaterials. We define the design of nanomaterials enhanced by computational technology as “Computational Nanomaterials”. This approach utilizes computational technology to begin with the biological characteristics of diseases, identify the optimal nanomaterials through material-biological interactions, material-property relationships, and appropriate ligands or adjuvants, simulate the construction of ideal nanomedicines, and accurately analyze their physicochemical properties. This outlines the general design process of computational nanomaterials. Consequently, computational nanomaterials can significantly reduce the human, financial, and time costs associated with traditional “verified guessing” methods, thereby advancing the field of nanomedicine. This aligns perfectly with the vision of “smart materials”.

**Scheme 3:**
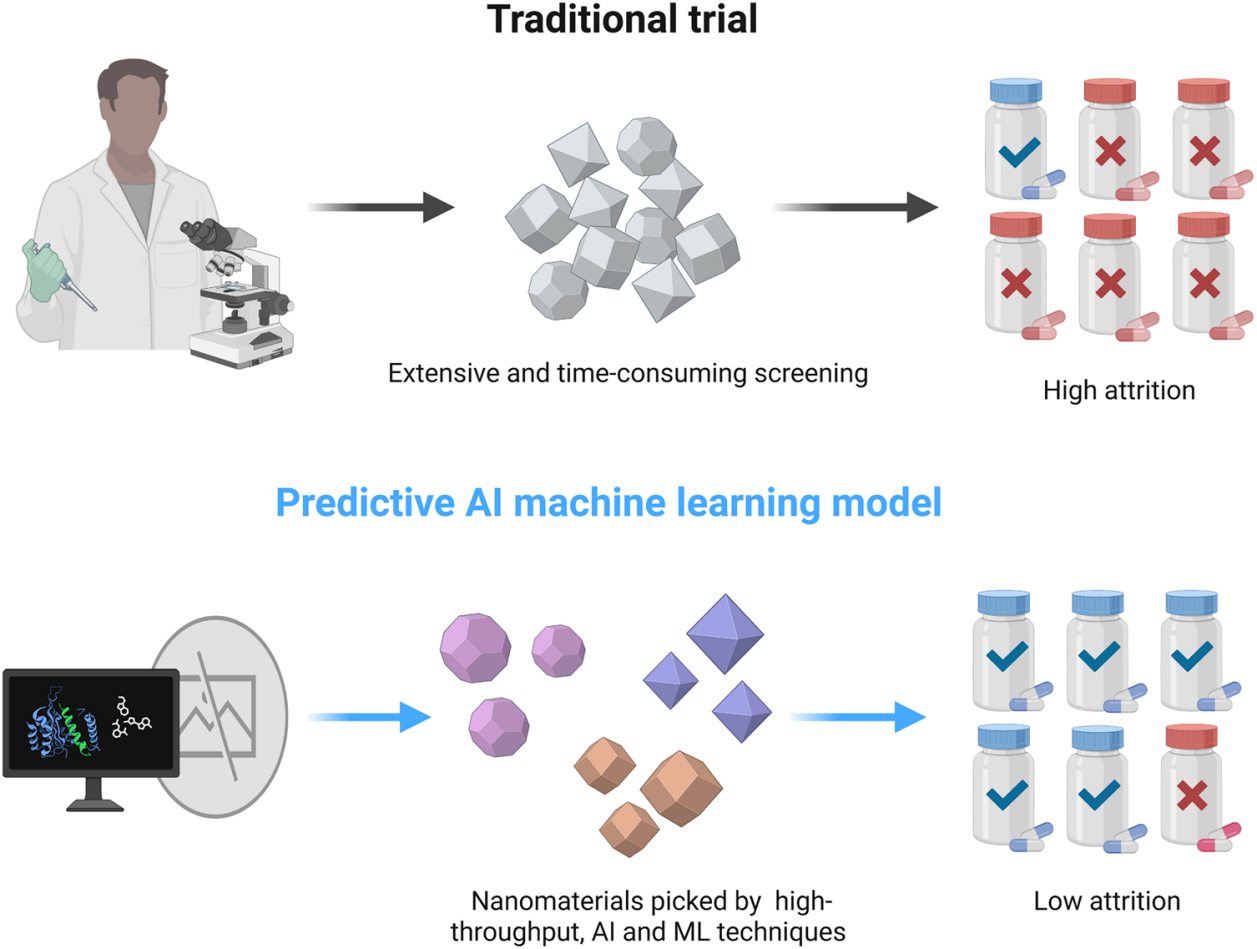
Schematic representation of computational nanomaterials. This approach involves predicting the physicochemical properties of various nanomaterial types and their interactions with living organisms using high-throughput, AI, and ML techniques. The goal is to infer the distribution, bioactivity, and toxicity of nanosystems in vivo, ultimately guiding the selection of suitable nanomaterials. Created with BioRender.com

Currently, computational technologies and algorithms for nanomaterial design have been increasingly applied to solid materials, including high-throughput, AI, and ML technologies. These tools can predict the physicochemical properties of various nanomaterials and their interactions with living organisms, enabling the prediction of nanosystem distribution, bioactivity, and toxicity, and ultimately facilitating the screening of suitable nanomaterials (Scheme 3). However, regardless of the technique, designing computational nanomaterials requires extensive data collection, processing, and analysis from existing studies. In a data-driven paradigm, HTS enhances data sample load and processing efficiency while enabling automated data generation, storage, and analysis. Wang et al*.* proposed the energy level principle and the adsorption energy principle, experimentally verifying their ability to predict the superoxide dismutase (SOD)-like activity of metal-organic frameworks (MOFs). These principles can facilitate the HTS of nanozymes with specific mimetic enzyme activities [85].

Additionally, high-throughput approaches can be used to screen nanocarriers, adjuvants, ligands, and other components in large databases to identify optimal nanomedicine formulations. Qi et al*.* proposed a new model for high-throughput calculation of the binding free energy of solid-binding peptides (SBPs) to material surfaces. This model estimates the binding of the entire peptide to nanomaterials based on the free energy contributions of individual residues, enabling the screening of SBPs that bind strongly to materials with targeted affinity or selectivity [86]. Winter et al*.* employed a HTS method, nanoPRISM, to investigate the relationship between nanomaterials and cellular internalization. They found that the core composition of nanomaterials is the key determinant of their uptake into cells. Using a library of cell lines with different DNA sequence barcodes, along with 35 fluorescently labeled NPs featuring varying core compositions, surface chemistries, and sizes, they identified the cells through high-throughput genome sequencing to pinpoint the key characteristics of cells that internalize nanomaterials [87]. Additionally, high-throughput technology can serve as an efficacy evaluation tool for nanomaterials in reverse screening. INSIDIA 2.0, a high-throughput image analysis software developed by Perini et al*.*, quantifies tumor cell death by analyzing parameters related to the destruction of cancer spheroids. This allows for a morphological evaluation of the effect of graphene quantum dot PTT on glioblastoma (U87) and pancreatic adenocarcinoma tumors [88]. High-throughput techniques are essential computational methods that can be integrated with other intelligent approaches, such as active learning, to efficiently optimize a large number of candidate materials while minimizing human cognitive overload and bias. This integration helps overcome the limitations of traditional Edison-style and model system approaches [89].

Similarly, AI is a powerful computational technology capable of processing large data sets. Among its various approaches, ML is a key paradigm that enables the development of algorithms for mathematical modeling based on existing data. ML mimics the human ability to recognize patterns and process information, making it a valuable tool for understanding and predicting the material properties, pharmacological parameters, and biological effects of nanomedicines. This approach offers a promising means to accelerate the development of desired NPs [90]. Depending on the type of data being modeled, ML models include unsupervised, supervised, semi-supervised, and reinforcement learning, among others. These mathematical algorithms, when applied to data, mimic human learning and uncover data-driven patterns, ultimately enabling accurate predictions of the corresponding outcomes [91]. Saeedimasine et al*.* calculated the adsorption free energies of 33 small biomolecules on nanomaterials using a molecular dynamics-meta-dynamics approach. They then applied various unsupervised learning algorithms, along with supervised linear and nonlinear regression algorithms, to construct a predictive model for extrapolating the adsorption free energies of other biomolecules on nanomaterials. Due to its accurate predictive performance, this ML model offers a method for classifying nanomaterials based on their interactions with biomolecules [92]. Fahmy et al*.* developed multivariate regression algorithms to predict the performance and trapping efficiency of specific types of NPs by analyzing early research data and applying supervised ML. Among the models, the one using the CatBoost algorithm for estimating the trapping efficiency of nanomaterials demonstrated the best performance. It also identified the drug-to-lipid ratio and lipid-to-surfactant molar ratio as key factors influencing trapping efficiency. Therefore, supervised ML proves to be an effective tool in assisting the design of nanomaterials to enhance nanodrug trapping rates and simplify experimental procedures [93].

By learning from past experiences and continuously optimizing its processes, ML develops “intelligence” over time, enabling it to solve tasks involving high-dimensional data, particularly clustering, classification, and regression. This capability allows ML to reveal data-driven insights and make accurate predictions. As a result, AI-assisted synthesis of nanomaterials is becoming increasingly “intelligent,” meeting key criteria such as good physicochemical properties, minimal biotoxicity, and a stable pharmacokinetic profile, ultimately leading to enhanced efficacy [94, 95]. Nuhn et al*.* identified vascular permeability heterogeneity among different tumor types through single-vessel analysis using AI, providing a foundation for the rational design of protein nanoparticle-based drug delivery systems to enhance nanomaterial permeability in tumors [96]. Additionally, the structure of chemical molecules in nanomaterials dictates the effectiveness of nanomedicines. Upon entering the body, the absorption, distribution, metabolism, and excretion (ADME) processes of nanomedicines are influenced by material–tissue interactions, which present uncertainties. However, the pharmacokinetics of nanomedicines is crucial in determining their biological effects. Therefore, constructing quantitative structure-activity relationship (QSAR) models combined with physiologically based pharmacokinetic (PBPK) models using AI techniques offers an intelligent approach to nanomaterial design. Lin et al*.*’s research team employed a ML approach to generate PBPK models that predict the ADME properties and toxicity of nanomaterials in tumors. They also used the models to infer the tumor delivery efficiency of different NPs based on the physicochemical properties of the materials and the cancer type [97]. Additionally, they developed an AI-based QSAR model using ML and deep neural network algorithms, which was integrated with a physiologically based PBPK model to simulate and calculate the tumor-targeting delivery efficiencies and biodistribution of various NPs [98].

In summary, these intelligent computational techniques are pivotal for designing smart materials. The algorithmic screening of nanomaterials, along with their adjuvants or ligands, supported by large datasets, and the experimental validation of model materials closest to the expected therapeutic efficacy, help minimize biases caused by human cognition. This approach narrows the potential parameter space and reduces the costs associated with trial and error. Therefore, computational nanomaterials hold great potential for guiding the design of “smart” nanomaterials in the future, paving the way for new advancements in nanomedicine.

### Smart Synthesis Methods

To prepare “smart” nanomaterials, the progressive advancement of synthesis technologies is a crucial step toward realizing smart nanomedicine. Traditional bottom-up and top-down synthesis methods are classified as chemical and physical approaches, respectively. The use of chemical reagents in chemical synthesis presents safety concerns, which limit its application, while physical synthesis methods struggle to achieve precise control over nanomaterials, thereby failing to meet the diverse requirements of “smart” synthesis. In recent years, novel concepts for nanomaterial synthesis, such as biosynthesis, biomimetic self-assembly, and biomimetic mineralization, have been experimentally validated and employed in the synthesis of complex nanosystems. For instance, inorganic nanomaterials are synthesized using natural components derived from plant extracts or microorganisms, such as bacteria [99]. Additionally, the self-assembly of materials can be initiated using biomolecular templates (proteins, DNA). These synthesis methods eliminate the need for large quantities of chemical stabilizers and the use of extreme synthesis conditions. Furthermore, these straightforward, safe, energy-efficient, and mild approaches present the potential for large-scale production of nanomaterials. Additionally, intelligent synthesis based on biological and biomacromolecular components can precisely regulate the structure, properties, and functions of materials. Moreover, it enables autonomous and responsive synthesis of materials.

#### Biosynthesis

**Scheme 4:**
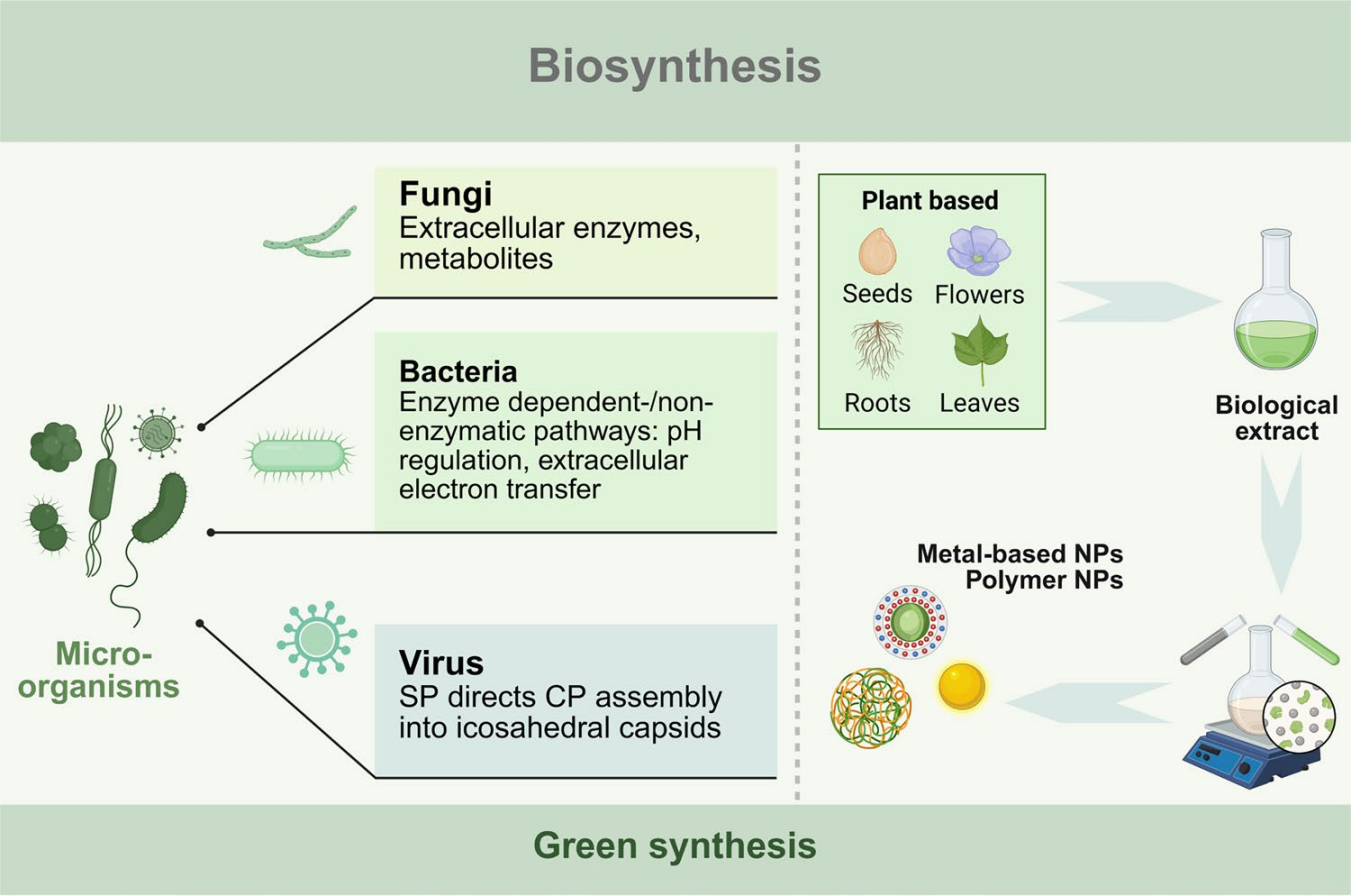
Schematic diagram of biosynthesis methods for nanomaterials. This process involves the synthesis of nanoscale functional materials using plant extracts and microorganisms, including fungi, bacteria, and viruses. Created with BioRender.com

The biosynthesis method of nanomaterials involves utilizing plant extracts, algae, fungi, bacteria, and viruses to produce nano-sized functional materials [100]. Unlike traditional chemical and physical synthesis methods, biosynthesis offers a green synthesis pathway that effectively utilizes natural biological resources to synthesize or assemble nanomaterials with specific functionalities within living organisms, using biological components as raw materials (Scheme 4). Microorganisms, including bacteria, fungi, viruses, and other biological entities, possess nanoscale components that carry out a variety of processes, such as the ingestion of external targets, energy production, and metabolite synthesis. Under complex survival conditions, these behaviors serve as self-protective mechanisms initiated by microorganisms. After absorbing essential nutrients from the environment, microorganisms expel toxic substances, such as transition metal ions, via exocytosis or use bioisolation to convert nonessential or even toxic substances into harmless forms. Additionally, a variety of metabolites and biomolecules produced by microorganisms may possess biological activities that facilitate the transformation of other substances. Interestingly, by strategically leveraging the biological functions of microorganisms, these organisms can, under appropriate conditions, use environmental substances to construct corresponding nanomaterials, which are categorized as extracellular and intracellular based on their site of synthesis [101]. In this process, toxic chemicals and artificially manipulated synthesis parameters are eliminated, making the nanomaterial synthesis both convenient and environmentally friendly. Consequently, the biosynthesis of nanomaterials, or green synthesis, is increasingly used in constructing sustainable nanostructures [102].

Fungi produce a wide range of biomolecules during their life processes, including various extracellular enzymes and metabolites with heterogeneous properties, which have been extensively shown to reduce metal ions into metal-based NPs. The biosynthesis of NPs by fungi is energy-efficient, occurring under mild temperature and pressure conditions, making it far superior to chemical synthesis. Additionally, fungal biosynthesis enables precise control over the NPs’ crystallinity, shape, and size, overcoming the limitations of traditional physical synthesis methods. Moreover, the metabolic biomolecules produced by fungi can regulate the synthesized NPs, providing unique surface properties and enhanced bioactivities. For example, antibiotics produced by fungi can synergize with metal-based NPs to exhibit antimicrobial effects [103]. Vahabi et al*.* exposed Trichoderma reesei, a fungus, to a silver nitrate solution, prompting the fungus to produce extracellular enzymes and metabolites that catalytically reduced the silver ions in the solution to form solid metallic AgNPs [104]. The abundant extracellular enzymes of *Trichoderma reesei* enable high-yield, high-rate synthesis of AgNPs, offering superior scalability compared to traditional physicochemical methods. Fungi have demonstrated the ability to biosynthesize a wide range of metals, metal oxides, metal sulfides, and other metal-based sulfur oxide NPs. However, the specific mechanisms underlying their synthesis still require further investigation to establish a theoretical framework for large-scale biological production of nanomaterials. Similarly, bacteria can utilize various enzyme-mediated catalytic reactions to reduce metal ions, such as nicotinamide adenine dinucleotide (NADH)- or nicotinamide adenine dinucleotide phosphate (NADPH)-dependent reductases, peroxidases (POD), and terminal oxidases (OXD). In addition, bacteria can reduce metal ions, sulfur protoxide anions, or other elements through non-enzymatic pathways, such as extracellular electron transfer [105], or by regulating pH changes that dissociate intracellular protons, creating negatively charged sites for metal ion absorption [106].

Additionally, mammalian cells have been shown to possess the ability to synthesize nanomaterials through a similar mechanism, wherein endogenous cellular components mediate the formation of new substances. Duval et al*.* demonstrated the synthesis of Au-NPs in a single step using NADH, with sodium bicarbonate buffer employed to mimic the cellular growth environment and preserve biomolecule integrity. Notably, the synthesis of Au-NPs relies entirely on endogenous biomass, eliminating the need for heating, pH adjustments, or molecular modifications. In the previous section, we discussed the biomimetic design approach involving viruses with sophisticated and precise features. Moreover, it can be also found that viruses can serve as synthetic templates, actively participating in the precise structural editing and functional construction of nanosystems [107]. Wang et al*.* developed a virus-like particle (VLP) material by using Salmonella typhimurium bacteriophage P22 as a template for nanomaterial synthesis. In this process, scaffold protein (SP) facilitated the self-assembly of coat protein (CP) subunits into icosahedral capsids. The interaction between CP and SP enabled the encapsulation and controlled delivery of protein cargo within the P22 VLPs by adjusting the composition and quantity of the protein cargo. Furthermore, they incorporated biocatalysts, such as enzymes, into the P22 VLPs, imparting new properties, including enhanced catalytic efficiency, substrate selectivity, and environmentally responsive behavior [108]. Thus, VLPs synthesized using viruses as templates offer a novel approach to the precise assembly of functional materials. These microbial synthesis methods, being biological in nature, minimize the need for artificial modification of inorganic or organic chemicals. They also mimic the inherent properties of living organisms, enhancing the biocompatibility of the materials and minimizing potential biotoxicity.

Microbial extracellular and intracellular synthesis of nanomaterials faces challenges in purification and yield, such as the accumulation or adsorption of metal NPs on the cell wall, which can hinder efficient isolation and purification. To address these issues, biosynthesis using plant extracts has emerged as a promising approach, offering advantages like simple synthesis, low purification costs, energy efficiency, high yield, sustainability, and enhanced biocompatibility. Plant extracts are a natural source rich in diverse bioactive substances, including sugars, glycyrrhetinic acid, thiols, lignin, phenolic compounds, and isoflavones [109], which play an active role in the nanomaterial synthesis process and can serve as green raw materials for producing functional nanomaterials [110]. Nishanthi et al*.* synthesized Ag, Au, and Pt NPs using the aqueous rind extract of *Garcinia mangostana* fruit, where the three metal NPs can combine with antibiotics to synergistically enhance antimicrobial effects [111]. In addition to metal-based NPs, polymeric NPs can also be synthesized through green chemistry principles. Tomato, which is rich in polyphenols, alkaloids, and ascorbic acid, was used by Abdallah et al*.* to produce CS NPs and zinc oxide (ZnO) NPs. These green-synthesized CS NPs and ZnO NPs demonstrated significant bacteriostatic activity against the rice pathogen *Xanthomonas oryzae pv. oryzae* [112].

#### Self-assembly Synthesis

**Scheme 5:**
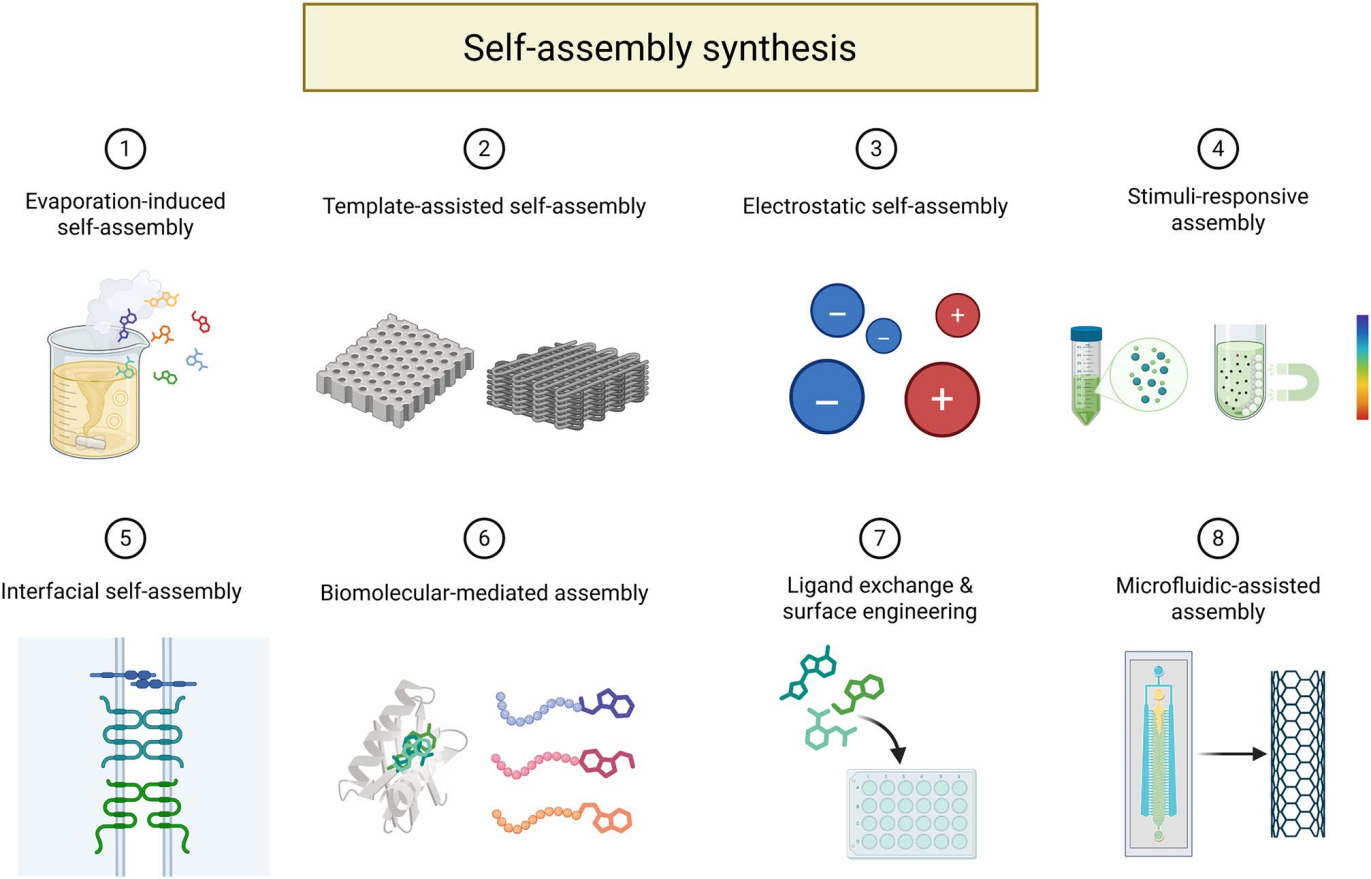
Schematic diagram of the self-assembly synthesis of nanomaterials. This process mimics the autonomous assembly behavior of biomolecules, leading to the formation of stable aggregation systems with specific structures and functions. The assembly is driven by either endogenous or exogenous forces, utilizing assembly components and biological templates. Created with BioRender.com

Self-assembly technology is a crucial tool in the “bottom-up” approach, where small structural units are orderly assembled into a coordinated nanomaterial from a limited number of components. In nature, many biological components are built step by step through self-assembly, such as proteins, nucleic acids, and polysaccharides—the three major biological macromolecules that are polymerized from simple constituent units like amino acids, nucleotides, and monosaccharides, respectively. Therefore, the self-assembly synthesis of nanomaterials follows a biomimetic approach, mimicking the autonomous construction behavior of biomolecules. This process efficiently utilizes structural units to spontaneously form stable aggregated systems with specific structures and functions. In this context, we will introduce the fundamentals of self-assembly in three parts: the components, the templates, and the assembly drivers (Scheme 5).

Currently, structural units commonly used for self-assembled nanomaterials include organic molecules (e.g., DNA, peptides, polysaccharides), inorganic compound molecules, and NPs, which are primarily bonded through non-covalent interactions to form various nanostructures in different morphologies such as 0D NPs, 1D nanotubes, nanofibers, and nanorods, 2D nanosheets, and 3D gels. These nanosystems, with their diverse morphologies, hold significant potential for biological and medical applications [113–117]. Nanomedicines assembled from natural molecules offer superior biocompatibility, sensitivity to microenvironmental changes, and structural versatility compared to other structural units. DNA, amino acids, proteins, peptides, and polysaccharides are commonly used for self-assembly into nanomedicines, owing to their intrinsic bioactivities such as antimicrobial properties and cell penetrability. These substances can also be self-assembled into nanocarriers capable of delivering various active ingredients, including genes, proteins, and small molecule drugs. The biologically derived components of these carriers can significantly enhance the bioavailability of the nanomaterials.

In recent years, with the continuous advancement of biomaterials, biomimetic mineralization technology has become increasingly mature. This technology synthesizes bio-inorganic nanomaterials by simulating the natural biomineralization process. A common approach involves using biological macromolecules or nanostructures self-assembled from biomolecules as templates. Positively charged inorganic mineralizer ions, molecules, or NPs spontaneously adsorb onto the surface of these templates. The reaction kinetics during inorganic nucleation and crystallization are carefully controlled through the template effect, facilitating the formation of biomimetic materials that mimic both the structure and function of natural biomolecules [118]. Self-assembled DNA nanostructures, with diverse morphologies ranging from 2 to 3D forms, can act as templates to guide the formation of structurally complex mineralizations, making them highly attractive for biomimetic mineralization [119]. Notably, DNA origami plays a key role in constructing various inorganic mineralizations, largely due to its structural flexibility, which enables precise modulation of inorganic substance binding sites to achieve specific nanostructure configurations. For instance, Shang et al*.* demonstrated that positively charged prehydrolyzing products (PP) exhibit a stronger electrostatic affinity for protruding double-stranded DNA (dsDNA) than for the surface of DNA origami. This results in preferential adsorption of PP onto the protruding dsDNA, and the synthesized silica nanostructures precisely replicate the dsDNA pattern. As a result, site-specific siliconization of DNA nanostructures enhances the potential for precise synthesis of inorganic nonmetallic nanomaterials [120]. Additionally, peptide templates with a high affinity for inorganic substances not only facilitate the self-assembly of nanomaterials but also serve as functional elements to regulate the biological activity of nanosystems, among other applications [121]. In this context, the use of natural peptides or proteins, such as ferritin and BSA, has garnered significant attention. However, artificially designed peptide templates offer greater flexibility to meet diverse assembly requirements [122]. Wilson et al*.* designed a bimodal catalytic peptide (SurSi) with two functional modules: a surface-active peptide sequence to stabilize oil emulsions in water; a biomineralizing peptide sequence to regulate the formation of a mineralized silica shell. The silica nanocapsule, encapsulating magnetic iron oxide NPs (IONPs), was synthesized through SurSi-induced biosilicification of the nanoemulsion. This approach protects the encapsulated particles from degradation and prevents premature release, offering an efficient method for encapsulating hydrophobic particles [123].

Using vapreotide acetate (Vap), an antitumor peptide, as a template, Yin et al*.* synthesized Vap-Au nanoflowers (Vap-AuNFs) through a bionanomorphic mineralization method. The resulting nanoflowers exhibit an anisotropic structure with a large absorption cross section, significantly enhancing the photothermal conversion efficiency of the nanocomplexes under NIR light irradiation and improving the antitumor efficacy of PTT. Additionally, Vap, a synthetic peptide, mitigates the biotoxicity of metal-based nanomaterials, providing a beneficial feature contributed by the synthesized template [124]. Template molecules play a critical role in the self-assembly of nanostructures, significantly influencing the structure and properties of the entire nanosystem. Biomimetic mineralization utilizes inorganic mineralizers derived from organisms to form nanomaterials through internal mineralization within subcellular structures. This approach aligns with the principles of green synthesis, as it minimizes the use of excessive chemical reagents and simplifies the labor-intensive steps of manual operation, making it an “intelligent” synthesis strategy.

The process of self-assembling nanostructures, composed of various structural components, depends on different driving forces and factors that influence assembly efficiency. These factors include the physicochemical properties of the component molecules, ionic concentration in the synthesizing environment, and biomolecular composition, among others. We categorize these factors into exogenous and endogenous factors that promote nanomedicine self-assembly. Endogenous factors refer to the intrinsic properties of the molecules themselves. The characteristics of small molecules used as assembly components can fundamentally affect the physicochemical properties and biological effects of the self-assembled system. For instance, DNA molecules with a double-helix structure and peptides with different amino acid configurations exhibit chiral properties, which can influence the properties of the assembled nanomedicines in two ways. As an example, Xie et al*.* designed a self-assembled heterochiral antimicrobial peptide (AMP) containing D-/L-amino acids. This nanostructured antimicrobial drug exhibited optimal activity against both Gram-negative and Gram-positive bacteria [125].

Additionally, the hydrophobicity of the assembled component molecules plays a key role in promoting self-assembly. When hydrophilic molecules are converted into hydrophobic substances through specific treatments, their ordered aggregation rate increases, thereby enhancing self-assembly efficiency. As a result, hydrophobic molecules such as aromatic amino acids, long-chain alkyl amino acids, and certain lipids act as natural self-assembling substances. For example, typical hydrophobic amino acids are important motifs in the design of self-assembling peptides [126]. Wang et al*.* designed and synthesized an aggregation-induced emission luminescence gene (AIEgen) conjugated self-assembling peptide (TPA-FFG-LA) targeting the epidermal growth factor receptor (EGFR). In this design, the aromatic amino acid sequence Phe-PheGly (FFG) serves as a hydrophobic molecule, which drives the self-assembly of this nanodrug. Upon light irradiation, the aggregated AIEgens generate large amounts of ROS, which mediate lysosomal membrane permeabilization and trigger immunogenic cell death. This process effectively kills EGFR-negative tumor cells and inhibits the growth of triple-negative breast cancer [127]. When the structural unit itself lacks the ability to self-aggregate, appropriate modification becomes a viable strategy for synthesizing nano-assembled structures. Cholesterol, a typical lipid molecule, can be used to modify carboxymethyl CS by incorporating histidine-cholesterol esters. This modification creates a hydrophobic structural domain that reduces the critical micelle concentration, promoting the curling of long-chain carboxymethyl CS polymers and the formation of encapsulated NPs. This approach improves nanostructural self-assembly and enhances cytoplasmic transport [128].

Additionally, the β-sheet conformation facilitates peptide assembly through electrostatic and hydrophobic interactions [129]. A peptide known as β tail can serve as a tool molecule to enable the precise assembly of target molecules to which it is attached. This is based on the principle that the interactions between the peptides are strengthened when the β tail transitions from an α-helix structure to a β-sheet conformation, thereby enhancing the driving force for self-assembly [130]. Although some structural units inherently possess a certain degree of assembly ability, appropriate modifications can significantly improve assembly efficiency and drug release properties. In summary, the construction of nano-assembled structures depends not only on the inherent nature of the structural units but also on the use of peptides and other tool molecules, as well as the optimization of synthesis conditions. These factors are crucial for enhancing the assembly efficiency, drug release properties, and biological effects of the entire system.

“Intelligent” self-assembly refers to a process where, under specific physiological conditions, stimuli-responsive nanocomponents autonomously form regular connections or undergo morphological transformations, resulting in highly efficient assembly of nanosystems. Unlike the “stimulus-responsive nanomaterials” discussed in the previous section, the stimulus responsiveness described here is embedded in the synthesis process of the nanomaterials, rather than their functional action, making it a more front-end form of nanomaterial intelligence. However, the stimuli driving both types of response are similar, including factors such as pH, temperature, magnetic fields, ionic concentration, and biological composition [131, 132]. As assembly elements in the self-assembly of nanomaterials, biomolecules can not only arrange and combine based on their own interactions, but also undergo stimulus-responsive dynamic rearrangement under complex environmental conditions. In other words, intelligent synthesis occurs when exogenous self-assembly driving forces influence the system. For example, DNA and peptides can undergo conformational and chemical changes in response to physical, chemical, and biological factors, which in turn affect their assembly state [113, 133]. Biomolecules play a crucial role in the multifunctional synthesis of nanostructures. Nanosystems that use biomolecules as assembly elements, synthetic templates, or responsive synthetic components are highly intelligent, with a synthesis process that is both convenient and environmentally friendly. For example, Zhan et al*.* employed an enzyme-dependent self-assembly technique to enable the dephosphorylation of adamantane-peptide coupling (Nap-FYp-Ada) by alkaline phosphatase expressed on *Staphylococcus aureus* (*S. aureus*), which triggered the in situ self-assembly of nanostructures. The resulting nanodrugs targeted the cytoplasmic lipids of *S. aureus*, leading to the depolarization and permeabilization of its cytoplasmic membranes, ultimately helping to resist *S. aureus* infection [134]. In a hydrophilic long-chain polymer, tertiary and quaternary amines are randomly arranged to respond to variations in pH, temperature, and ionic composition in the environment. These changes trigger intramolecular self-folding and intermolecular self-assembly, ultimately resulting in the formation of self-assembled nanostructures, with the polymers acting as the building blocks [135].

## Toxicity

**Scheme 6:**
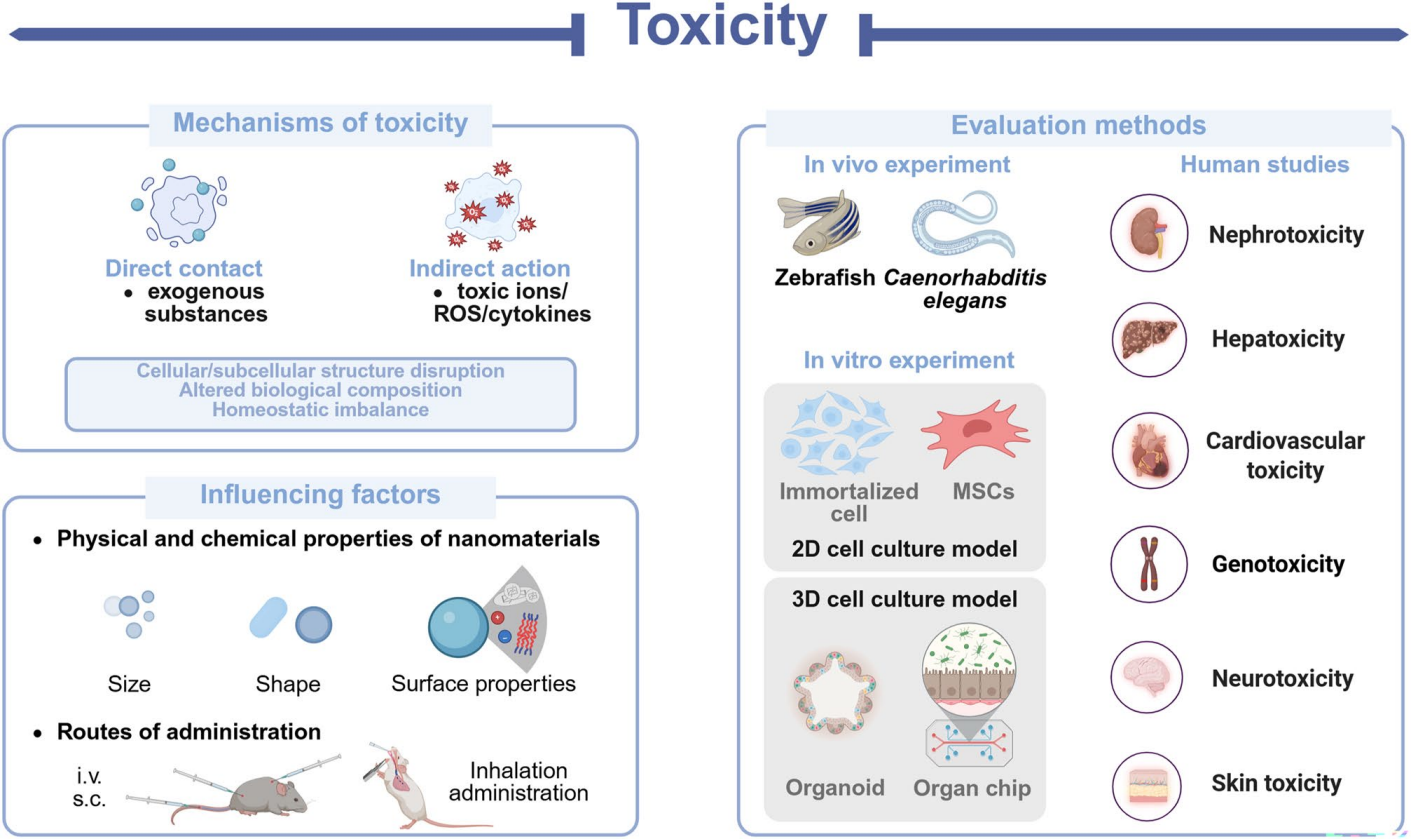
Schematic diagram of toxicity problems of nanomaterials. This diagram illustrates the intrinsic mechanisms of toxicity, both direct and indirect factors, and the methods used for their accurate assessment. Created with BioRender.com

Nanomaterials have attracted significant attention in the fields of food safety and biomedicine. However, as research progresses, concerns regarding their potential adverse effects have arisen, presenting new challenges for researchers. When nanomaterials interact with organisms, they can trigger a range of well-documented or yet-to-be-understood toxicity mechanisms. These mechanisms may lead to toxic side effects at various levels (organ, cellular, and subcellular) including cardiotoxicity, hepatorenal toxicity, gastrointestinal toxicity, developmental and reproductive toxicity, as well as pulmonary fibrosis [136]. The range of materials that have demonstrated toxic side effects is broad, including metal-based NPs, carbon-based nanomaterials, and silica-based nanomaterials, among others. This highlights the potential limitation of nanomaterial toxicity in the applications of nanomedicine. However, the toxicity of nanomaterials is not an insurmountable challenge. Both direct and indirect factors can contribute to or exacerbate toxicity, but these adverse effects can often be mitigated by controlling key variables. Therefore, accurately assessing and understanding the toxicity and underlying mechanisms of nanomaterials is crucial for guiding the design and synthesis of novel, safe, and effective nanomaterials. This review focuses on the mechanisms of toxicity of nanomaterials, their influencing factors, and their assessment methods (Scheme 6).

In this context, the emerging field of nanotoxicology investigates the toxicological impacts of nanomaterials and nanocomposites on living systems, providing valuable insights and technical guidance for the rational design of safer nanomaterials [137]. Nanotoxicology not only investigates the mechanisms of toxicity that impact human health but also explores the toxicological effects on other biological species. This broad spectrum of species enhances the modeling of toxicological effects, thereby improving the accuracy and robustness of toxicity evaluation systems. As a result, it helps reduce the adverse effects of nanomaterials and broadens their potential applications.

### Mechanisms of Toxicity

These mechanisms play a crucial role in the multisystem toxicity observed in organisms. For example, in cardiotoxicity, a well-established mechanism involves the accumulation of nanomaterials in the heart, which triggers oxidative stress and inflammation. This leads to mitochondrial damage at the subcellular level, resulting in adverse effects such as apoptosis and, ultimately, cardiac damage in animal models [138]. The primary mechanisms of action of carbon-based nanomaterials at sub-cytotoxic concentrations include DNA damage, oxidative stress, and protein stress [139].

Additionally, ROS play a central role in the toxicity induced by nanomaterials. An increase in ROS levels not only rapidly triggers oxidative stress but also acts as a toxicant, mediating various forms of damage, including DNA damage and mitochondrial stress. Research has demonstrated that MSNs can induce oxidative stress by generating ROS through the NADPH OXD and MAPK signaling pathways, leading to significant alterations in the intestinal microbiota of mice [140]. In addition to the overproduction of ROS, the release of toxic ions by nanomaterials can act as an indirect mechanism of toxicity, particularly in metal-based nanomaterials. Once inside the organism, metal-based NPs can undergo structural breakdown in the in vivo environment, releasing metal ions from their core structure and triggering a cascade of adverse reactions. Furthermore, some of these ions can bind to proteins and enzymes, inhibiting their normal functioning [141, 142]. Nanomaterial-mediated biotoxicity is often manifested as damage to genetic material in organisms, primarily in the form of DNA damage. This, in turn, activates the DNA damage response network, leading to cell cycle arrest and apoptosis. For instance, quantum dots can damage DNA structure either through their nanomorphology or by releasing metal ions, which can disrupt the DNA repair process and prevent proper damage repair [143].

At the level of other biomolecules, the strong affinity of nanomaterials for various components of the organism enables direct interactions that can affect the function of biomolecules. For example, dendrimers (dendritic molecules) exhibit a strong binding affinity for vitamins, amphiphilic lipids, bile acids, and proteins, which may contribute to their toxicity [144]. Additionally, based on the blood biochemical results of organisms, nanomaterials have been shown to induce electrolyte and metabolic disturbances. In rats treated with varying concentrations of Fe_3_O_4_-TiO_2_ NPs, electrolyte profiles revealed a significant decrease in potassium levels [145]. Cytotoxicity resulting from the dysregulation of cellular calcium homeostasis is a significant concern for nanomaterials, given the crucial role of Ca^2+^ in intercellular signaling. In neurons exposed to quantum dots, intracellular calcium levels were elevated, leading to neuronal apoptosis. This may be due to the direct or indirect interaction between quantum dots and cell membranes, which affects ion channels, causing an influx of extracellular calcium and the release of intracellular calcium, ultimately disrupting cellular calcium homeostasis [146]. In terms of metabolism, prolonged exposure to graphene oxide (GO) triggers changes in the tricarboxylic acid cycle (TCA) cycle and in the key substances involved in TCA, leading the body to develop problems common to tumor cells [147]. Therefore, the toxicity of nanomaterials can be assessed through metabolomics approaches to evaluate their potential impact.

The direct damage caused by nanomaterials upon initial contact with an organism is a significant source of toxicity. For instance, direct interaction between cells and exogenous substances can lead to cell membrane damage and morphological changes, which may also affect tissues and organs. Generally, NPs with a large surface area adhere to the cell membrane or wall, causing lipid peroxidation of membrane lipids. This process gradually disrupts the outer cell structure and ultimately compromises the integrity of the cell [148]. Since the liquid-ordered structural domains of the cell membrane are essential for signaling in both prokaryotic and eukaryotic cells, nanomaterials may disrupt these domains, impairing the phospholipid bilayer and interfering with normal cellular functions [142]. Studies have shown that nanomaterials can induce atherosclerosis (AS)-like lesions under certain conditions through a complex mechanism involving both direct and indirect effects. Direct contact with blood vessels disrupts the endothelial layer of the vascular wall, leading to endothelial dysfunction via leakage and pro-inflammatory activation. Additionally, once the integrity of the endothelium is compromised, nanomaterials can penetrate the subendothelial space, triggering phenotypic switching, proliferation, and migration of vascular smooth muscle cells, thereby influencing plaque development [149]. In addition, Ti_3_C_2_T_x_, a type of MXene, has been shown in in vitro studies to disrupt erythrocyte morphology, causing sustained extrusion and cell deformation. Ti_3_C_2_T_x_ also alters the secondary structure and conformation of BSA, γ-globulin, and fibrinogen, leading to significant complement and platelet activation [150]. Therefore, nanomaterials can directly damage both cells and biological components.

While the toxicity mechanisms of nanomaterials can cause multisystem damage in target organisms, they also offer effective therapeutic strategies in antibacterial and anticancer treatments [151, 152]. Thus, the toxic effects of nanomaterials act as a double-edged sword, necessitating careful consideration under various application conditions to optimize their beneficial biological activities.

### Influencing Factors of Toxicity

The mechanisms underlying the toxicity of nanomaterials are still under investigation, which is crucial for expanding their application potential. Although the toxicity mechanisms are not fully understood, several contributing factors have been identified, offering potential solutions to mitigate the adverse effects and guiding future research into these mechanisms. We have summarized the primary factors influencing toxicity, particularly the physicochemical properties of nanomaterials. For example, the role of nanoparticle size (<100 nm) in toxicity is well established, as smaller nanomaterials can easily penetrate cells and tissues, disrupting cellular structures and potentially causing erythrocyte membrane rupture and hemolysis [150, 153]. However, larger particles (>100 nm) are primarily located on cell surfaces [141] and are more likely to cause blockages in the vascular system due to their size [141, 154]. When nanomaterials are internalized by cells or tissues, they can elevate ROS levels, thereby decreasing cellular activity and inducing cytotoxicity [140, 155].

In addition to size, the shape and surface properties of nanomaterials are well-established factors influencing toxicity. Rod-shaped materials, for example, often exhibit higher biotoxicity. The rutile form of TiO_2_ NPs, for instance, has demonstrated potential cytotoxicity and can impair the normal function of primary rat hepatocytes [156]. This may be due to differences in metabolic processing rates across various shapes, with rod-shaped nanomaterials being more prone to accumulation in the body, leading to a range of unintended biological effects. In contrast, globular materials are metabolized more quickly but can exhibit more lethal toxicity under certain conditions [154, 157].

Additionally, surface functional groups and coatings can influence the biotoxicity of a material. For example, microspherical BiOCl shows weaker particle–membrane interactions compared to nanosheet BiOCl. However, the hydroxyl groups on the surface of microspherical BiOCl strengthen its interaction with cell membranes, causing membrane damage and significantly increasing the toxicity [158]. Although surface modification of nanomaterials is commonly used to enhance biocompatibility, it can occasionally introduce toxicity. For instance, when Prussian Blue (PB) NPs are surface-modified with PEI, the resulting positively charged NPs upregulate POD activity, negatively impacting cell viability as well as liver and kidney functions [159]. Generally, positively charged NPs tend to accumulate more readily in vivo than negatively charged ones. However, the surface charge of quantum dots affects their biodistribution following intravenous administration in mice: positively charged quantum dots primarily accumulate in the lungs, while negatively charged quantum dots preferentially target the liver, potentially leading to hepatotoxicity [156]. Similarly, positively charged monodisperse silica NPs (+34 mV) are rapidly excreted from the liver into the gastrointestinal tract, whereas negatively charged NPs (-18 mV) tend to remain in the liver [157]. While it is well established that the toxicity of nanomaterials is influenced by their physical and chemical properties, the specific relationship between each property and its corresponding toxicity effect has not yet been accurately quantified. As a result, the linear relationship between material properties and toxicity remains poorly understood. Consequently, regulating toxicity based on these properties is imprecise, as it does not allow for precise control over toxicity levels, which often fluctuate within a certain range. Addressing this challenge could be a key focus for future research, as overcoming nanomaterial toxicity is crucial for their successful application in the biomedical field.

In addition to the intrinsic properties of nanomaterials, their toxicity is significantly influenced by environmental factors, including both intracellular and extracellular conditions such as temperature, light, and pH [160, 161]. These factors regulate the release, physicochemical transformations, and metabolic processes of nanomaterials, thereby amplifying or mitigating their toxic effects. For example, Zhang et al*.* demonstrated that pH and temperature synergistically enhance the toxicity of commercial TiO_2_ NPs to algae through mechanisms such as physical adsorption, oxidative stress, and toxin release [162]. Importantly, this environmentally responsive toxicity fundamentally differs from the behavior of engineered stimuli-responsive materials. The latter are designed for controlled responses, whereas the former results from passive interactions with environmental variables, potentially leading to unintended adverse outcomes.

Shifting the focus from environmental interactions to biological exposure dynamics, the interaction between nanomaterials and biological systems—referred to as the exposure process—serves as the critical entry point for nanomaterial uptake by organisms and can significantly influence toxicity outcomes. The administration routes, as active exposure strategies, play a crucial role in determining the intensity and target-specific toxicity of nanomaterials. For example, silicon-based nanomaterials exhibit route-dependent toxicity. MSNs induce acute immunotoxicity and inflammation when administered intravenously or intraperitoneally, but have negligible effects when administered subcutaneously [163]. Similarly, intravenous administration of mSiO_2_ NPs triggers hepatotoxicity through ROS overproduction and activation of the NLRP3 inflammasome, whereas inhalation or oral exposure avoids these effects [156]. These findings highlight the pivotal role of administration routes in shaping toxicity profiles. Moreover, both dose and duration are key determinants of nanotoxicity. Dose-dependent biphasic effects are often observed, where low concentrations may provide essential trace elements that promote cellular proliferation, while exceeding threshold levels can induce oxidative damage to membranes and metabolic dysfunction [164]. Temporally, nanomaterial toxicity can manifest as either acute effects (primarily oxidative stress) or chronic effects (such as multiorgan dysfunction and cumulative damage) [165].

Crucially, variability in experimental conditions and assessment models significantly impacts the accuracy of nanomaterial toxicity evaluations, which is a critical consideration for mechanistic studies and risk assessment. Specifically, the cytotoxicity of NPs is highly dependent on the evaluation system. Cancer cell lines with faster proliferation rates exhibit heightened sensitivity to nanomaterials compared to normal cells, leading to a systematic overestimation of toxic effects [166]. Simplified monoculture models, such as Caco-2 monolayers, which lack physiological components like mucus barriers or immune cell interactions, may overestimate toxicity due to direct exposure mechanisms. In contrast, complex models that incorporate multicellular systems or organoids better mimic the dynamic homeostasis of in vivo microenvironments, providing toxicity data more consistent with animal studies [167]. Furthermore, interspecies and interindividual differences in metabolic rates and immune responses can significantly alter the toxicokinetic profiles of nanomaterials, even under identical exposure conditions [168]. Therefore, a tiered strategy that integrates cross-species models and personalized assessment approaches is recommended for accurate toxicity evaluation.

### Toxicity Evaluation Methods

Accurate toxicity evaluation is crucial for defining the applications of nanomaterials [169]. Toxicity depends on both the intrinsic properties of nanomaterials and their biological targets, but its intensity can vary across different methods, highlighting the need for standardized assessments. Lower organisms, such as eukaryotes and vertebrates, provide practical in vivo models that replicate physiological conditions, offering realistic toxicity profiling. These models strike a balance between experimental feasibility and biological relevance, bridging lab findings with real-world interactions and ensuring reliable safety evaluations for clinical applications. Caenorhabditis elegans, a soil-dwelling nematode and lower eukaryote, possesses vertebrate-like nervous, digestive, immune, and reproductive systems. Its rapid cultivation, genetic tractability, and transparent body make it ideal for comprehensive systemic, cellular, and molecular toxicity assessments of nanomaterials [170, 171]. Zebrafish, a higher vertebrate model, offer biological relevance due to their rapid breeding, translucent embryos, and conserved developmental pathways. Their external fertilization enables studies on embryogenesis, disease modeling, and drug screening, making them essential for investigating nanomaterial toxicity mechanisms across different levels of biological complexity [172]. In summary, model organisms provide a cost-effective approach for nanomaterial toxicity screening, reducing the reliance on mammals and accelerating preclinical testing. However, interspecies physiological differences (e.g., the absence of circulatory/respiratory systems) limit their predictive accuracy for biodistribution and systemic effects, hindering their translation to mammalian outcomes despite ethical and operational advantages. Furthermore, the inability of lower organisms to fully replicate the coordinated inter-organ interactions and complex homeostatic mechanisms seen in higher animals underscores the continuous need for human-relevant toxicity models to thoroughly evaluate the clinical translational potential of nanomaterials.

Human studies are the most effective way to assess nanomaterial toxicity through multiorgan/system analyses, but they face cost and risk barriers that limit direct exposure data. Contemporary research predominantly centers on observational epidemiology, including cohort and case-control studies, to examine exposure-disease associations, with comparatively fewer experimental trials designed to elucidate mechanistic insights. Cohort studies, especially prospective and retrospective designs, strengthen causal inference by linking nanomaterial exposure to health outcomes over time. This hybrid approach balances ethical constraints while advancing biocompatibility evaluations, which are crucial for assessing the clinical safety of nanomaterials [173]. Squillacioti et al*.*’s cohort study involving 136 workers exposed to nanomaterials demonstrated that 10 years of exposure was associated with pulmonary decline. The IL-10/TNF-α ratio mediated the association between exposure and FEV₁/FVC decline through an anti-/pro-inflammatory imbalance [174]. Moreover, experimental epidemiological studies, regarded as the gold standard for causal inference, employ controlled interventions, randomization, as well as intergroup, dose–response, and longitudinal analyses to determine safety thresholds and delineate nanomaterial toxicity pathways, thus laying a foundation for risk assessment [175].

In vitro cell-based experiments precede in vivo studies by predicting the required number of animals and screening appropriate nanomaterial doses. NR8383 macrophages model immunotoxicity by linking immune responses, while bivalve hemocytes, with their innate immunity and specialized endolysosomal systems, uniquely model nano-immunotoxicity through the internalization of nanomaterials. These models bridge cellular mechanisms to systemic toxicity assessments [176]. Human cells (e.g., lung/colorectal cancer, bronchial epithelial, monocytes) outperform cross-species models in nanotoxicity studies by expressing human-specific genes and metabolic pathways, reducing preclinical bias. These clinically relevant systems allow for multiorgan toxicity assessments, bridging molecular mechanisms to human disease pathology [170, 171].

Stem cell models are highly effective in nanotoxicity studies owing to their increased nanoparticle uptake and heightened sensitivity. Their self-renewal and differentiation capacities facilitate multiorgan assessments, including neurodevelopmental and cardiotoxic effects via embryonic stem cells, as well as adipogenic or osteogenic impacts via MSCs. These models provide ethically favorable and human-relevant toxicity profiling [177]. Stem cell models enable rapid in vitro nanotoxicity screening across tissues. As seed cells, they generate organoids—3D self-organized micro-organs in ECM scaffolds—that replicate physiological structures and multitissue interactions, overcoming the limitations of 2D models for more accurate safety assessments [178, 179]. Emerging technologies, such as organoids and organ-on-a-chip systems, have advanced the in vitro replication of cellular interactions, secretory profiles, and microenvironmental dynamics. These innovations show promise in replacing animal testing, providing reliable alternatives for toxicological research [180, 181].

Despite rapid advances in in vitro and in vivo nanomaterial toxicity models, their correlation remains limited. In vitro studies can examine controlled interactions but lack the ability to extrapolate to animal models, as they do not provide direct comparability or causal evidence, thus hindering translational validity [169]. Nanomaterial toxicity exhibits model-dependent variability with unresolved mechanisms, complicating toxicological studies. Integrating smart technologies—such as computer simulations, HTS, and ML—enables efficient multi-indicator analysis across systems, reducing time and cost while addressing experimental variability, ensuring robust safety assessments. Joossens et al*.* combined HTS with high-content imaging (HCI) to simultaneously capture multiple toxicity indicators in cell-based assays, surpassing conventional single-readout methods. HCI allows the concurrent analysis of large cell populations using multiplexed fluorescent probes, generating quantitative data from microscopic images. This approach helps elucidate complex cellular mechanisms by tracking parameters like cell survival, membrane permeability, apoptosis, mitochondrial potential, and steatosis in nanomaterial-exposed HepaRG cells. The resulting dataset integrates in vitro toxicity results with the physicochemical properties of nanomaterials, advancing quantitative property–activity relationships (QPARs) for comprehensive safety evaluation [182]. Similarly, the integration of HTS and high-content analysis techniques facilitates the investigation of cellular uptake and cell-to-cell transfer through automated imaging and image analysis. This approach further facilitates the prediction of dose-dependent and time-dependent toxic effects of nanomaterials [183].

ML, a subset of AI, analyzes multivariable correlations within large datasets to build predictive computational models. This data-driven approach mimics human learning while processing high-dimensional data at a much faster rate [184]. By correlating nanomaterial properties with toxicity, ML builds sophisticated models to predict nanotoxicology, offering efficient and scalable solutions for safety assessment. Zhou et al*.* developed ML-PEMST, a regression model that predicts the ecotoxicity of metal-based nanomaterials by integrating physicochemical properties, environmental factors, and cross-species interactions. It processes heterogeneous datasets, enabling innovative toxicity assessments [185].

## Application

Innovative nanomaterials address a wide range of medical challenges by regulating ROS, enabling targeted delivery, and offering multifunctionality. These materials are applied in the treatment of infections, wound healing, and disorders related to neurodegeneration, cardiovascular health, immunity, and the hepatorenal system [186–188]. In wound repair, nanomaterials regulate ROS by scavenging excess levels while maintaining moderate amounts, dynamically inhibiting infections and promoting regeneration [189]. For neurodegenerative diseases, sub-5 nm particles with engineered surfaces improve brain distribution and inhibit protein aggregation [190], while integrated antioxidants disrupt oxidative stress cycles by neutralizing mitochondrial ROS [191, 192]. Cardiovascular therapies utilize ROS-responsive NPs to neutralize oxLDL and reduce monocyte infiltration [193], along with targeted delivery to minimize systemic side effects. In immune disorder management, synovium-targeted drug retention inhibits NF-κB, while light-controlled platforms enable spatiotemporal modulation of ROS [194]. In inflammatory bowel disease (IBD), pH/enzyme-responsive carriers localize drug delivery to gut lesions, while integrated NPs scavenge ROS, inhibit NF-κB, and reduce colitis relapse [195, 196]. Hepatorenal injury therapies leverage antioxidant–anti-inflammatory synergy to repair damage and prevent fibrosis, coupled with size-dependent renal excretion to avoid accumulation [197, 198]. This review categorizes soft and hard nanomaterials and their applications in refractory inflammatory diseases, fostering new therapeutic paradigms.

### Hard Nanomaterials

Hard nanomaterials (e.g., metal NPs, carbon-based materials, MSNs) excel in biomedical applications due to their mechanical strength, stability, and surface properties. They outperform soft materials in electromagnetic response, drug loading, and shear resistance, effectively addressing challenges in cardiovascular diseases and the blood-brain barrier (BBB). Despite challenges related to biodegradability, surface functionalization and composites facilitate the delivery of high-stiffness, precision medicine solutions.

#### Metallic NPs

**Fig. 1:**
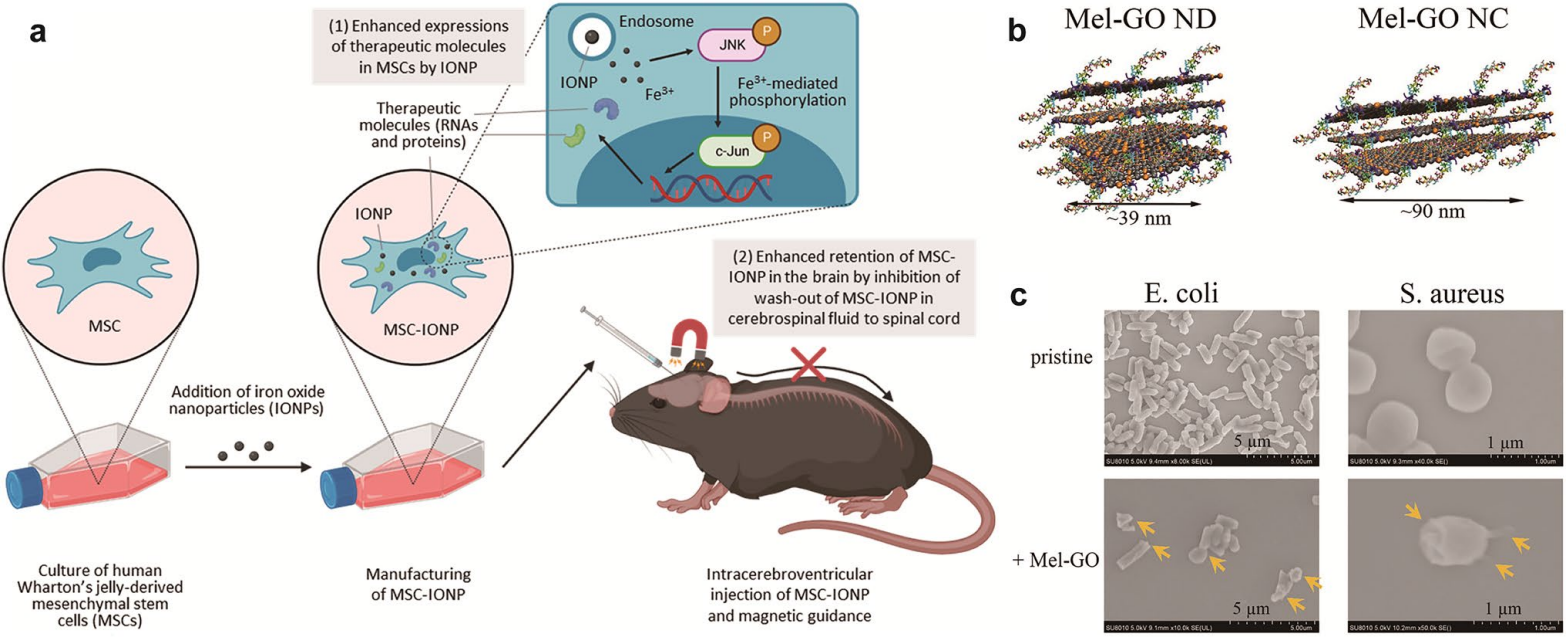
**a** Schematic illustration depicting the fabrication of MSC-IONP and their application in the treatment of AD [204]. Copyright 2023, American Chemical Society. **b** Diagram illustrating the Mel-GO ND or NC complex with varying sizes and numbers of nanosheet layers. **c** SEM images of *E. coli* and *S. aureus* bacteria, both prior to and following treatment with Mel-GO NDs. Arrows indicate membrane lesions and collapses in the bacterial cells [206]. Copyright 2019, John Wiley and Sons

Metal-based nanomaterials, including metallic and metal oxide NPs, exhibit unique biological activities and engineering potential in various medical applications. In anti-infective therapy, metal oxide NPs (e.g., cerium dioxide) disrupt bacterial membranes through Ce^3+^/Ce^4+^ redox cycling [188], while synergistic combinations with antibiotics or AMPs, such as ZnO@PEP-MPA nanoprobes, enhance bactericidal efficiency [199]. In wound healing, Ag-ZnO NPs synthesized with *Azadirachta indica* leaf extract [200] and ZnO NPs-coated sutures prepared using *Acacia modesta* gum [201] show exceptional wound repair capabilities. For neurological disorders, Au NPs exhibit therapeutic potential through β-amyloid inhibition [202] and enhanced BBB penetration [190], with plant-synthesized variants further reducing neuroinflammation [203]. Magnetic IONPs facilitate targeted drug delivery, as demonstrated by MSC-guided magnetic navigation systems, which improve Alzheimer’s disease (AD) therapy (Fig. 1a) [204]. In immune disease intervention, iron–quercetin coordination NPs (Fe-Qur NCNs) alleviate RA by suppressing the NF-κB pathway [194], while light-controlled nanosystems, such as folate receptor-targeted Au-DEN-MTX@IR780, enhance ROS generation under NIR irradiation to improve therapeutic efficacy [205].

#### Carbon-based Nanomaterials

Carbon-based nanomaterials, including graphene derivatives, CNTs, and carbon quantum dots, exhibit potent antimicrobial activity through ROS generation via photodynamic, photothermal, and photocatalytic mechanisms [207, 208]. These materials disrupt bacterial membranes and induce oxidative damage, with graphene coatings particularly inhibiting pathogens such as *Streptococcus mutans* while also promoting tissue regeneration [209]. Synergistic strategies enhance efficacy: hybrid composites, such as CeO_2_-GO, boost ROS production through redox cycling and charge separation [208], while AMP-functionalized graphene nanodots achieve membrane disruption with reduced cytotoxicity (Fig. 1b, c) [206]. Multifunctional nanohybrids integrating magnetic components, such as Fe_3_O_4_, with bioactive agents like nisin or Ag NPs, further enable targeted and sustained antibacterial action [210].

**Fig. 2:**
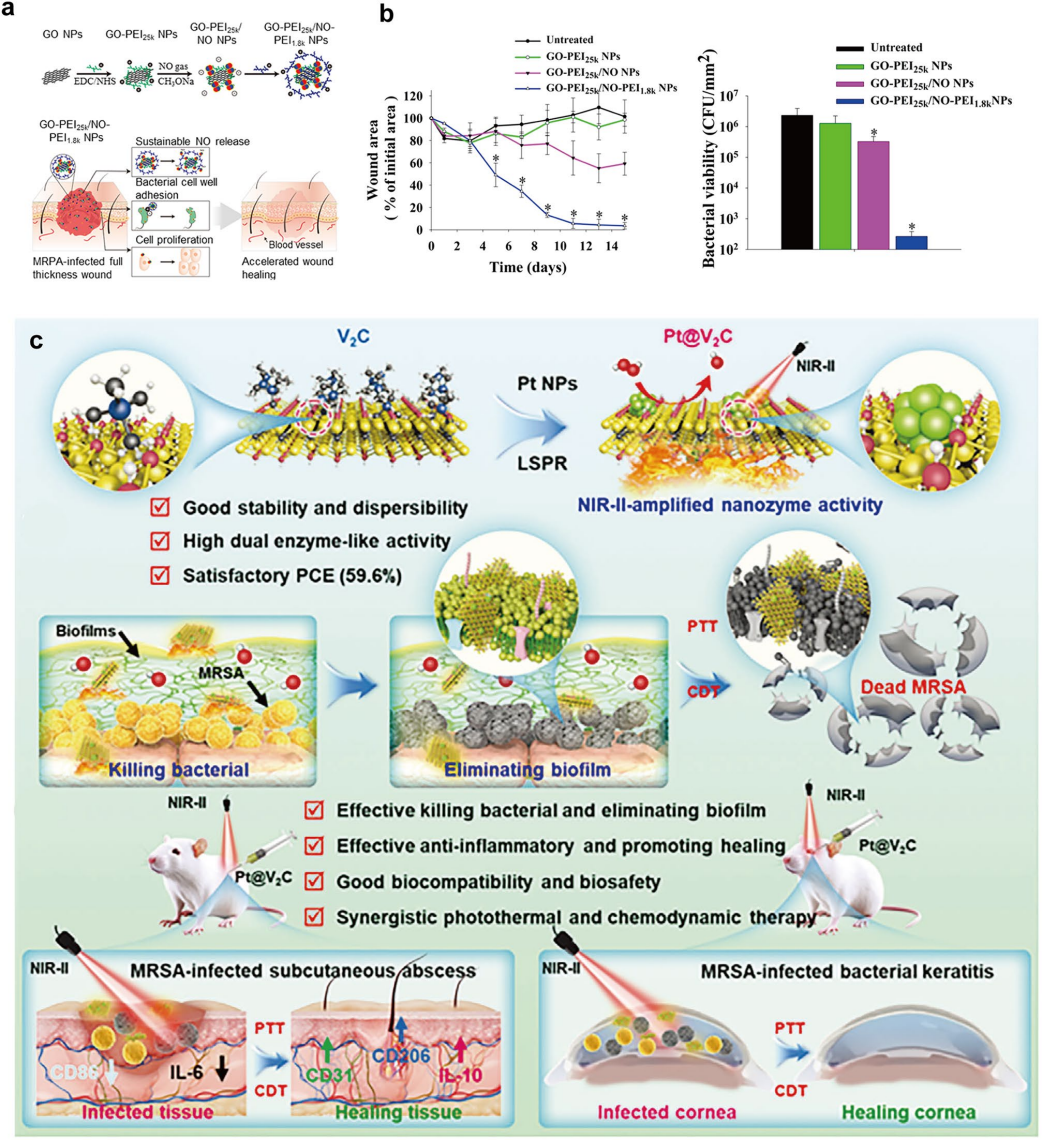
**a** Diagrammatic representation of the fabrication process and mechanism of action of GO-PEI25k/NO-PEI1.8 k NPs. **b** Healing effect and antimicrobial activity of GO-PEI25k NPs, GO-PEI25k/NO NPs and GO-PEI25k/NO-PEI1.8 k NPs in MRPA-infected wound in mice [211]. Copyright 2022, American Chemical Society. **c** Schematic illustration of the construction, antibacterial properties, and anti-infective therapy of Pt@V_2_C nanoplatforms utilizing photothermal and chemodynamic therapies [212]. Copyright 2024, John Wiley and Sons

In wound healing, GO-based hydrogels and nanocomposites promote healing by modulating oxidative stress and bacterial adhesion. For example, NO-releasing GO NPs target multidrug-resistant bacteria through electrostatic interactions while preserving biocompatibility (Fig. 2a, b) [211].

#### Nanozymes

Nanozymes mimic the catalytic activity of natural enzymes (e.g., OXD, POD) through metal or carbon-based nanomaterials. Surface engineering enhances their enzyme-like properties, despite compositional similarities to conventional counterparts, by combining nanomaterial stability with enzyme efficiency. These low-cost, multifunctional catalysts offer superior activity, size control, and ease of synthesis, with metal-based systems leading in medical applications. For example, in antibacterial therapy, nanozymes with POD-like activity can catalyze low concentrations of H_2_O_2_ (< 1 mM) into highly toxic •OH to eradicate bacteria [213]. OXD-like nanozymes can catalyze O_2_ into H_2_O_2_ and highly reactive O_2_•^−^/^1^O_2_, which exhibit strong antibacterial properties [214]. The Pt@V_2_C composite developed by He et al*.* exhibits dual-enzyme activities (POD and OXD), enabling synergistic NIR-II photothermal/chemodynamic therapy to combat *Methicillin-resistant Staphylococcus aureus* (MRSA) infections while modulating the immune microenvironment (Fig. 2c) [212].

**Fig. 3:**
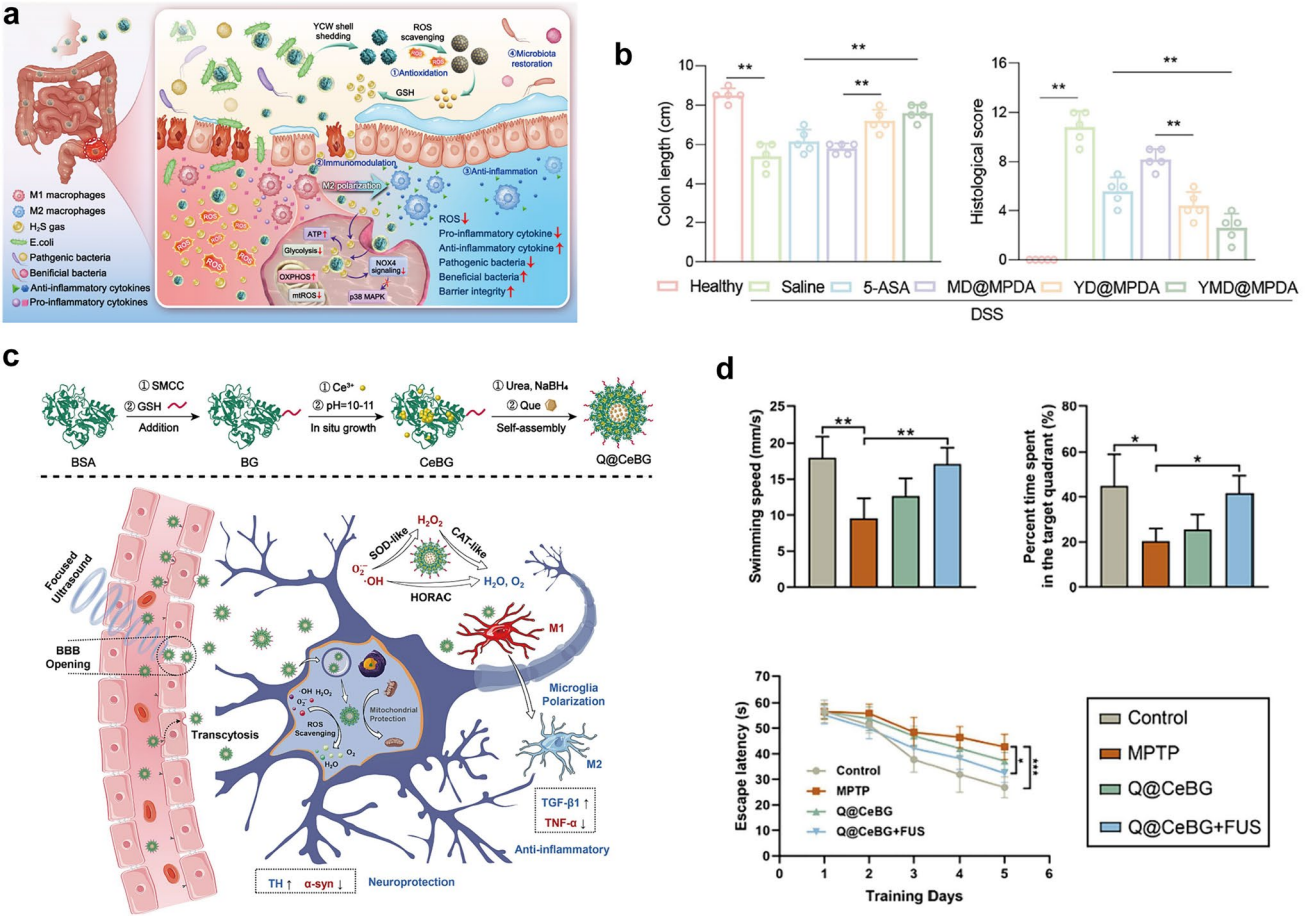
**a** Schematic illustration of orally administrated YMD@MPDA for targeted IBD therapy, involving ROS scavenging, anti-inflammatory, and immunomodulatory effects. **b** In vivo efficacy of YMD@MPDA in IBD: Quantification of colon length and histological scoring based on microscopic analysis of tissue morphology [217]. Copyright 2025, American Chemical Society. **c** Schematic representation of the preparation process for Q@CeBG nanoreactors and their therapeutic mechanisms in PD, focusing on neuroprotection and modulation of the brain microenvironment. **d** Behavioral assessment of Q@CeBG combined with FUS for the treatment of PD in mice. Representative data includes swimming speed, percent time spent in the target quadrant, and escape latency from the Morris water maze test [218]. Copyright 2024, Elsevier

The predominance of metal-based nanozymes arises from the unique advantages of metal active centers (e.g., Fe^3+^, Mn^2+^, Cu^2+^) in biomimetic catalysis, including tunable redox potentials, high electron mobility, and the precise engineering of enzyme-mimicking active sites. This review classifies metal-based nanozymes based on these active centers and explores their diverse medical applications. Manganese-based nanozymes with catalase (CAT) and SOD-mimicking activities serve as free radical scavengers in neurodegenerative diseases, reducing oxidative damage and neuroinflammation by converting •OH, O_2_•^−^ and H_2_O_2_ into non-toxic H_2_O and O_2_ [215]. Adhikari et al*.* developed citrate-functionalized Mn_3_O_4_ nanozymes (C-Mn_3_O_4_ NPs) for ROS and protecting mitochondria from oxidative damage, offering a novel strategy for managing Huntington’s disease [216]. For the treatment of IBD, nanozymes can outperform first-line medications, such as aminosalicylates, at equivalent doses. The oral yeast-derived YMD@MPDA nanocomposite delivers MnO_2_ nanozymes/H_2_S prodrugs to inflamed colons via β-glucan. It alleviates IBD by scavenging ROS, promoting M2 polarization, remodeling the microbiota, restoring barriers, and inhibiting the NOX4/p38 MAPK pathway, thereby synergizing antioxidant and gas therapies (Fig. 3a, b) [217].

**Fig. 4:**
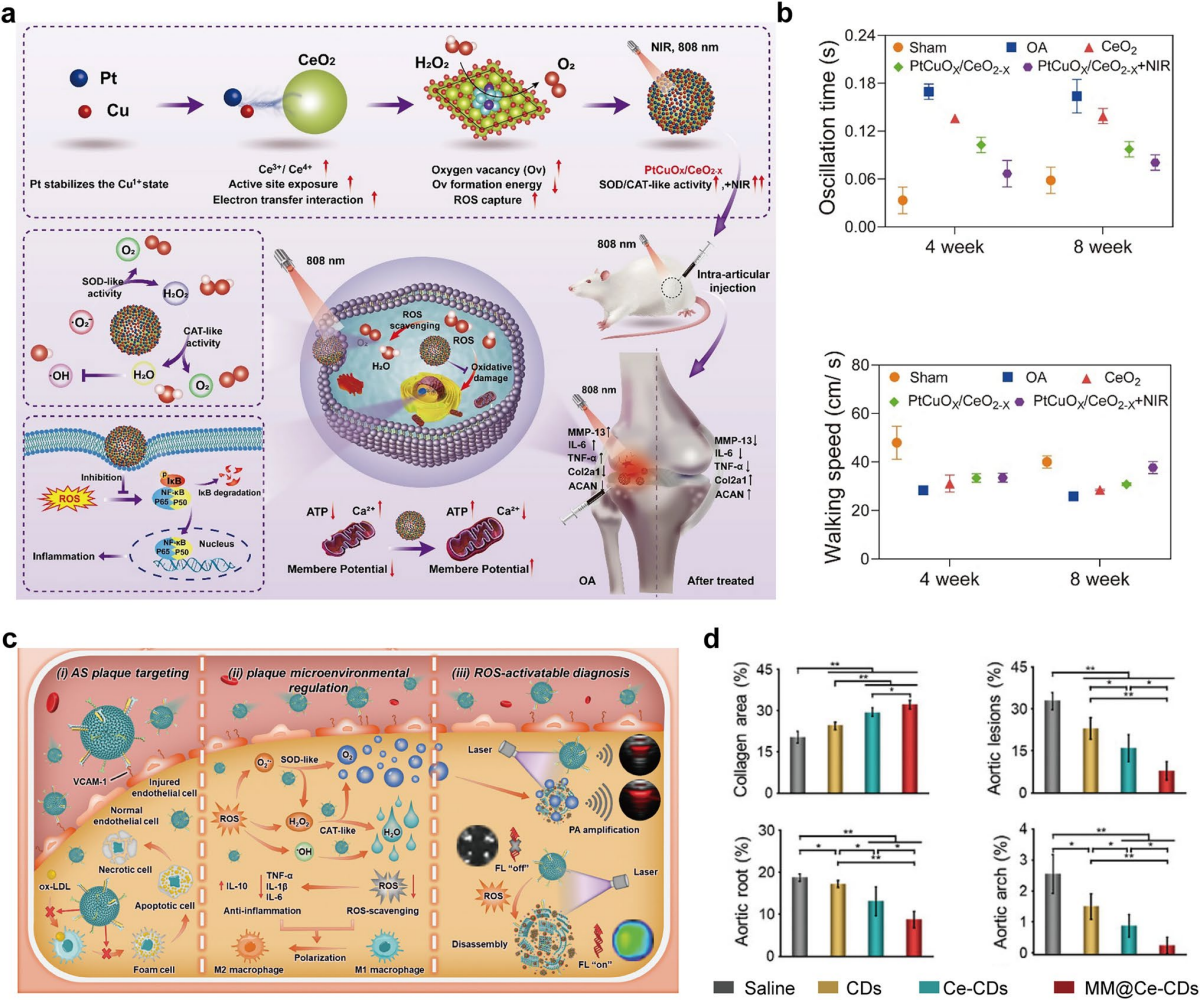
**a** Schematic illustration of PtCuO_X_/CeO_2-X_ nanozymes for the treatment of OA. **b** In vivo treatment with PtCuO_X_/CeO_2-X_ nanozymes to attenuate OA: left hind paw swing time and walking speed were measured via gait analysis after 4 and 8 weeks of treatment [220]. Copyright 2024, Springer Nature. **c** Schematic illustration of the synthesis of MM@Ce-CDs NPs and their role in targeted ROS-activated theranostics and regulation of the plaque microenvironment in AS. **d** In vivo synergistic therapeutic efficiency of MM@Ce-CDs NPs for the treatment of AS: quantitative analysis of relative lesion area of aortas, relative plaque area in cryosections from the aortic root, aortic arch, and the plaque collagen area [221]. Copyright 2025, John Wiley and Sons

**Fig. 5:**
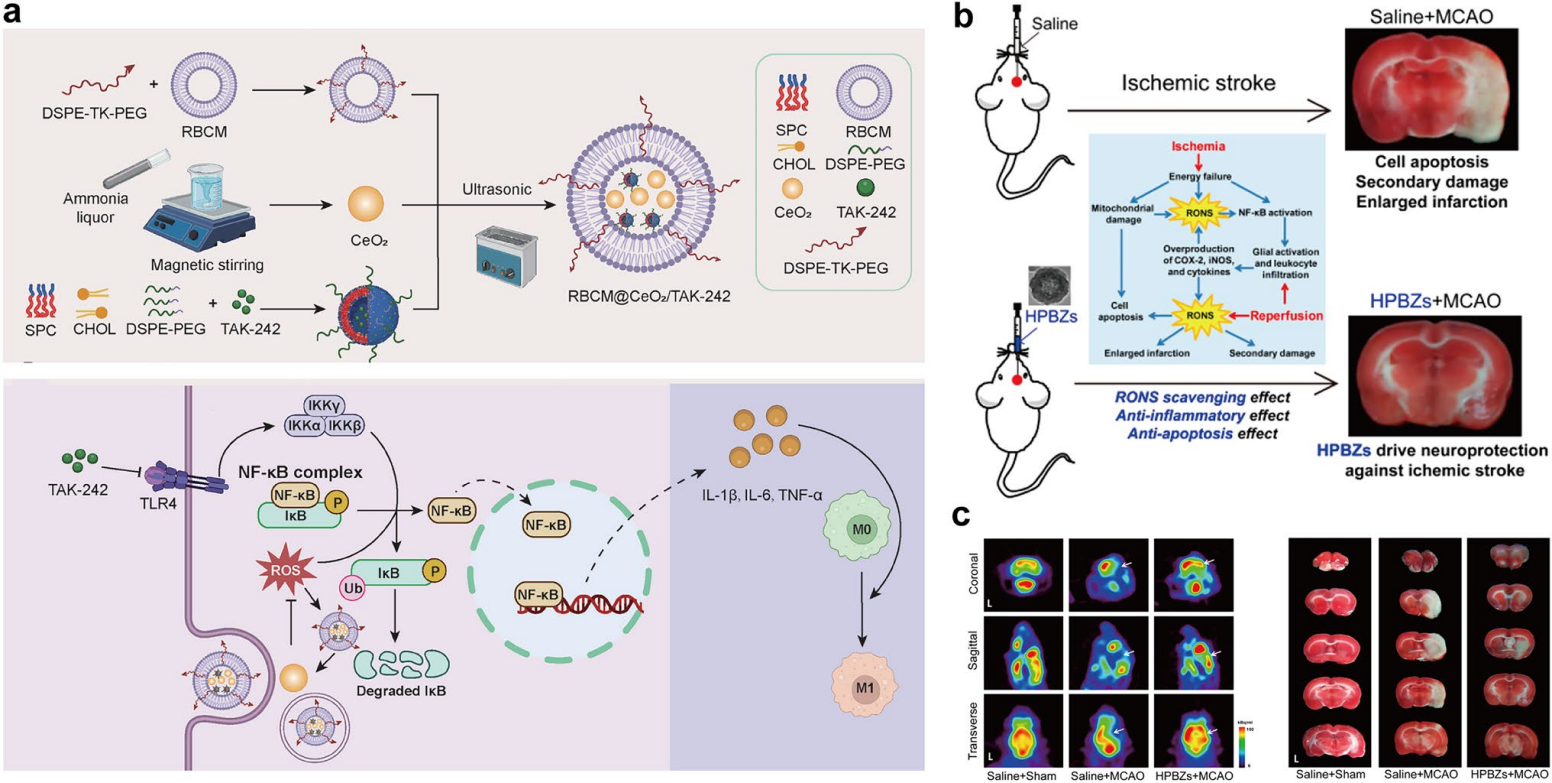
**a** Design, fabrication, and therapeutic mechanism of RBCM@CeO_2_/TAK-242 [222]. Copyright 2024, John Wiley and Sons. **b** Schematic diagram of HPBZs for the treatment of ischemia/reperfusion injury. **c** In vivo efficacy of RBCM@CeO_2_/TAK-242 for the treatment of ischemia/reperfusion injury: representative cerebral ^18^F-FDG PET images and photographs of TTC-stained coronal brain slices [223]. Copyright 2019, American Chemical Society

Cerium-based nanozymes, a classic metal-based catalytic material, exhibit enzyme-like activity through the valence transition of cerium ions (Ce^3+^/Ce^4+^). In nervous system diseases, Q@CeBG nano-reactor (CeO_2_/quercetin-loaded, GSH-modified BSA) with FUS-enhanced BBB penetration ability and ROS scavenge capacity could alleviate neuronal stress and promotes anti-inflammatory M2 microglial polarization, offering a multitarget therapeutic strategy for Parkinson’s disease (PD) (Fig. 3c, d) [218]. Due to the high levels of ROS associated with arthritis, nanozymes are poised to become highly effective antiarthritic therapeutics [219]. Cu/Pt-doped CeO_2_ nanozymes (PtCuO_X_/CeO_2-X_) enhance SOD/CAT-like activity and achieve 55.41% photothermal efficiency through oxygen vacancies. These nanozymes scavenge ROS/reactive nitrogen species (RNS), protect mitochondria, and inhibit ROS/Rac-1/NF-κB-mediated inflammation, demonstrating safe alleviation of osteoarthritis (OA) in vivo (Fig. 4a, b) [220]. To enhance the therapeutic efficiency of nanodrugs, targeting strategies have been strategically incorporated into the design of classical nanozymes. In cardiovascular diseases, a macrophage membrane-coated nanozyme (MM@Ce-CDs NPs) enables ROS-responsive targeted theranostics and regulation of the plaque microenvironment in AS, reducing inflammation/ROS and inhibiting foam cell formation via biomimetic delivery (Fig. 4c, d) [221]. Additionally, RBCM@CeO_2_/TAK-242 NPs (RBC membrane-engineered, TAK-242-loaded) target injured kidneys, suppressing CaOx crystals and renal injury through antioxidative and anti-inflammatory actions, as well as TLR4/NF-κB-mediated macrophage reprogramming. This dual efficacy and biosafety are validated in glyoxylate-induced mice (Fig. 5a) [222].

Iron-based nanozymes, exemplified by classic PB nanozymes, exhibit catalytic activity through the redox cycling of iron ions (Fe^2+^/Fe^3+^). These materials demonstrate multifunctional enzyme-mimicking behaviors, including POD, CAT, and SOD-like activities. In the treatment of neurological disorders, hollow-structured PB nanozymes (HPBZs) show enhanced ROS/RNS scavenging, suppress apoptosis and inflammation in vivo and in vitro, and improve ischemic brain tolerance with minimal side effects (Fig. 5b, c) [223]. In cardiovascular diseases, BSA@PB/Cur nanozyme can alleviate symptoms by scavenging ROS, inhibiting TNF-α/IL-1β, enhancing cholesterol efflux, and reducing plaque lipid and MMP levels in vivo [224]. For arthritis treatment, Cho et al*.* coated PB nanozymes with Pluronic to enhance their stability and uptake rate, thereby improving the therapeutic efficacy [225]. For liver damage, manganese PB nanozymes (MPBZs) with Mn^2+^ synergy scavenge ROS, modulate the Nrf2/NF-κB pathways, and prevent APAP-induced liver injury through nano-detoxification [226].

Single-atom nanozymes (SAzymes), with maximum atomic utilization efficiency, can generate ROS with superior selectivity and enzyme-mimetic activity to manage bacteria-infected wounds [227]. ROS can be generated by POD-like activity under acidic conditions and eliminated by CAT-like activity under neutral or alkaline conditions [228], promoting skin closure due to the amphoteric nature. Cu-g-C_3_N_4_ single-atom nanozyme (copper-decorated g-C_3_N_4_) exhibits glucose OXD/POD-like activities, generating ROS through cascades to eradicate drug-resistant bacteria [229].

**Fig. 6:**
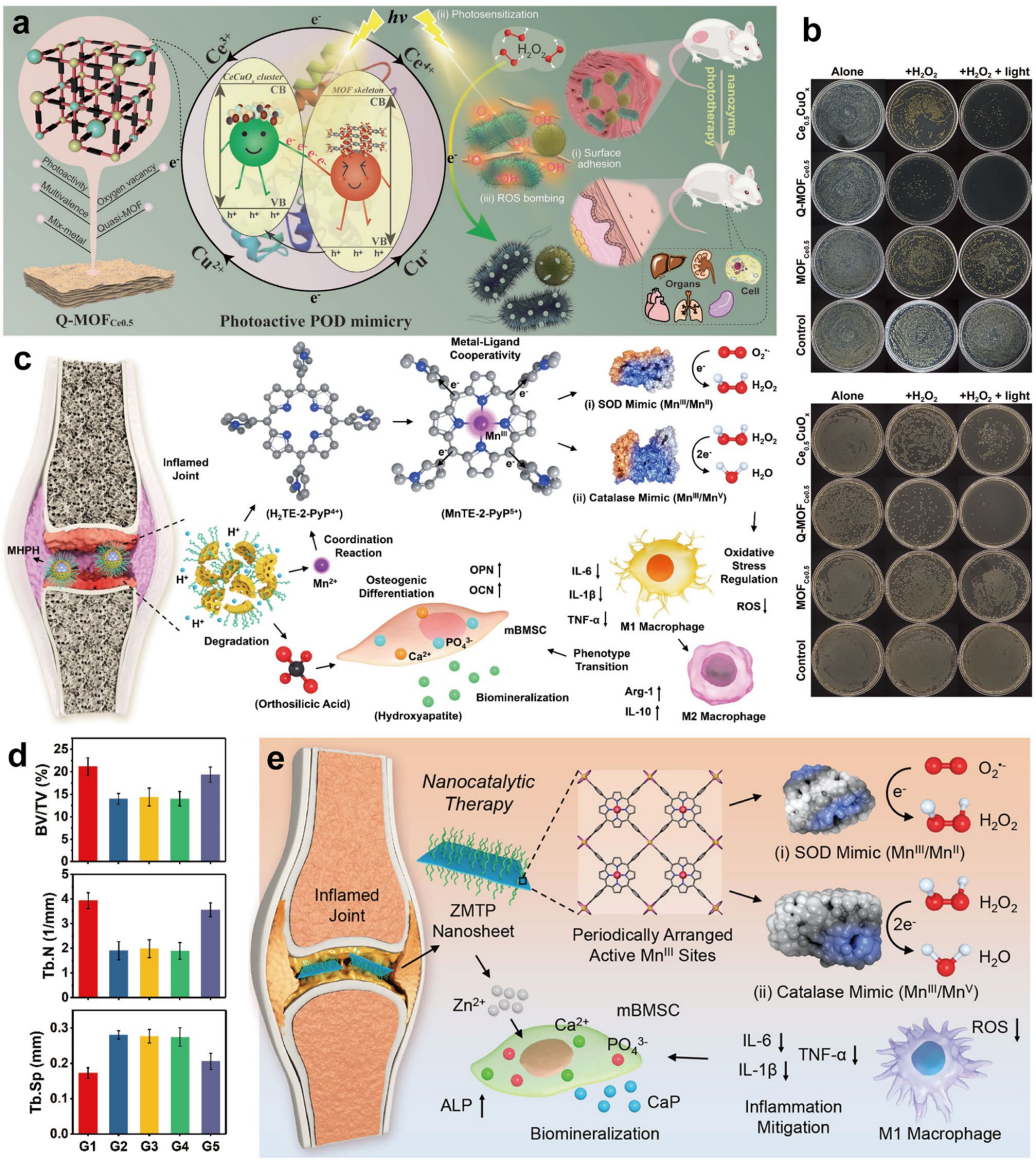
**a** Catalytic disinfection mechanism proposed for the 2D bimetallic quasi-MOF_Ce0.5_ nanozyme. **b** Plate count results showing the antibacterial effects of various nanozyme formulations against *E. coli* O157: H7 and *S. aureus* [231]. Copyright 2022, John Wiley and Sons. **c** Schematic illustration for the therapeutic concept of MHPH nanomedicine for catalytic anti-inflammatory treatments. **d** In vivo antiarthritic efficacy of MHPH: histomorphometric micro-CT analysis of fundamental parameters of bone microstructure (BV/TV, Tb.N, and Tb.Sp) [232]. Copyright 2022, American Chemical Society. **e** Therapeutic mechanism of ZMTP nanosheet for nanocatalytic RA treatment [233]. Copyright 2022, Springer Nature

MOF nanozymes, composed of organic linkers and metal clusters, demonstrate enzyme-mimicking catalysis through metal nodes, ligands, or their synergistic effects, enhancing substrate adsorption and mass transfer beyond conventional surface-dependent nanozymes. Essentially, MOFs can catalyze the POD substrate to produce toxic •OH, effectively killing bacteria [230]. For instance, the bimetallic Q-MOF_Ce0.5_ utilizes oxygen vacancies, multivalent redox cycles, and photoactive band structures to continuously generate ROS under visible light, effectively eliminating surface-adhered bacteria (Fig. 6a, b) [231]. In the treatment of RA, a Mn-engineered silica nanocarrier (MHPH) acid responsively releases porphyrins and manganese, forming SOD-/CAT-mimetic Mn porphyrin. This promotes the polarization of M1 macrophages to M2 macrophages and enhances biomineralization through silicon oligomers, as demonstrated in arthritis models (Fig. 6c, d) [232]. The 2D MOF nanosheet (ZMTP) mimics natural antioxidases (Mn-SOD/CAT) through manganese porphyrin coordination and zinc-modulated redox, exhibiting dual-enzyme activities, anti-inflammatory and pro-biomineralization effects, as well as antiarthritic efficacy in cellular and in vivo models of nanocatalytic therapy (Fig. 6e) [233]. However, although MOF nanozymes outperform conventional nanozymes in terms of structural designability and multifunctional integration, they still face challenges related to stability and scalability of production.

**Fig. 7:**
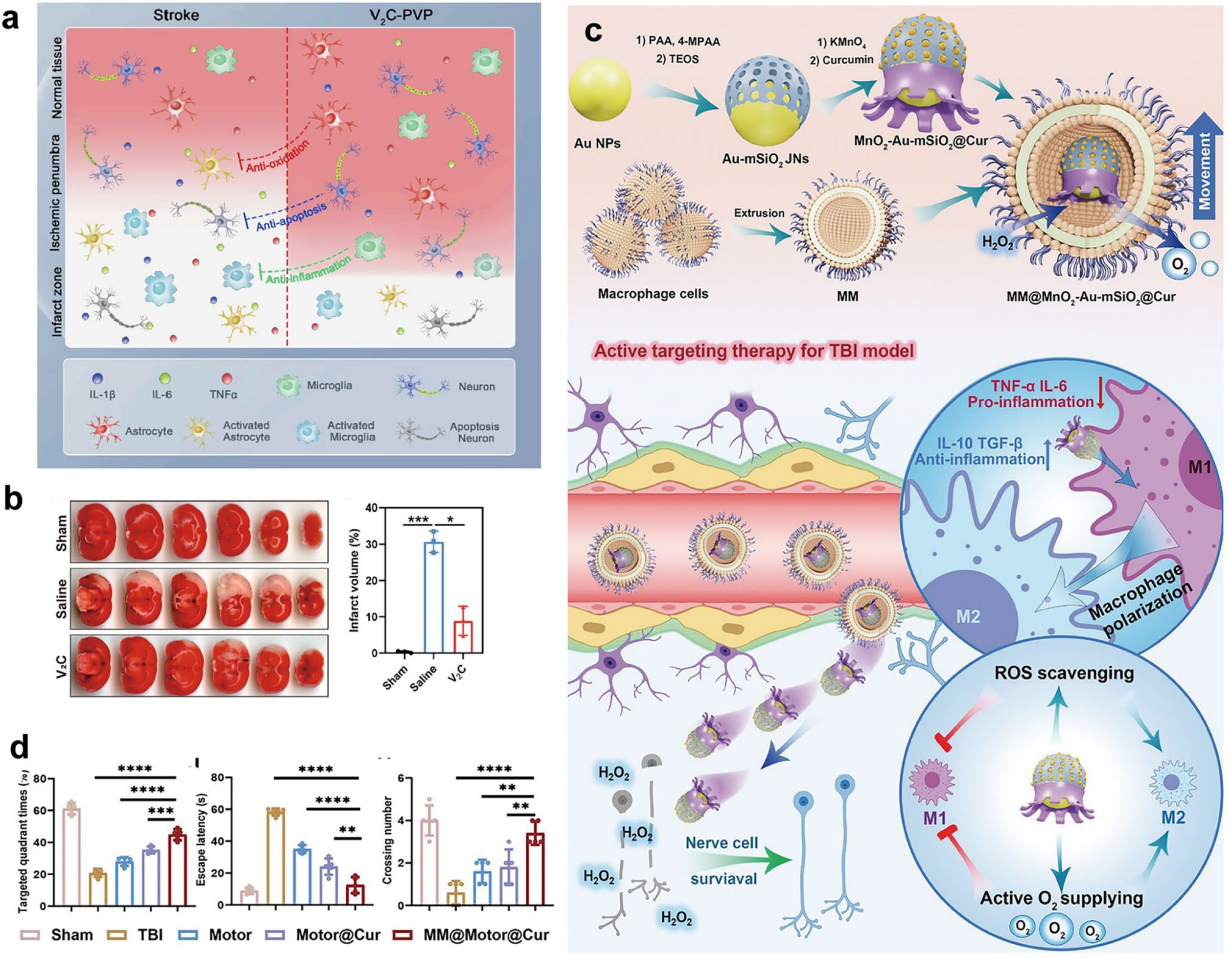
**a** Schematic illustration of 2D V_2_C MXene-based nanozyme with intrinsic multiple enzyme-like activities as a theranostic nanoplatform for ischemic stroke treatment, alleviating oxidative stress, suppressing cell apoptosis, and counteracting inflammation. **b** In vivo efficacy of V_2_C MXene for the treatment of ischemic stroke: representative images of TTC-stained coronal brain slides and quantitative calculation of the infarct volume [235]. Copyright 2022, Elsevier. **c** Schematic illustration of the fabrication process of MM@MnO_2_-Au-mSiO_2_@Cur and cascade-targeting anti-inflammatory therapy for TBI. **d** In vivo efficacy of MM@MnO_2_-Au-mSiO_2_@Cur for the treatment of TBI in the Morris water maze, including the time spent in the target quadrant, escape latency and the number of platform crossings [242]. Copyright 2024, John Wiley and Sons

Notably, MXenes exhibiting multienzyme mimicry (SOD/CAT/POD/GPx) use their negatively charged surfaces to attract metal ions, thereby facilitating the therapeutic targeting of oxidative stress and metal ion accumulation in neurological disorders [234]. 2D V_2_C MXenes scavenge ROS and reduce apoptosis and inflammation by mitigating oxidative stress, thereby preventing ischemic stroke (Fig. 7a, b) [235].

Nonmetallic nanozymes surpass metal-based counterparts in terms of biocompatibility and safety, rendering them effective for treating infections, neural disorders, and IBD [236–238]. Carbon-based variants provide theranostic integration and scalable production for the treatment of chronic and microbiome-related diseases, although their catalytic efficiency and biosafety require optimization for successful clinical translation [239].

#### Targeted NPs

Nanomaterials facilitate precise treatment of inflammatory diseases through intelligent design. Focusing on neurological and cardiovascular disorders that require precision, targeted strategies can bypass the BBB to concentrate drugs at lesions, enhancing efficacy while reducing toxicity—crucial for slowing neurological diseases with fewer side effects. Silanol-rich MSNs enable customized functionalization, improving drug loading, BBB penetration, and controlled release for biomedical applications [240, 241]. Ye et al*.* developed a macrophage membrane-modified SiO_2_/MnO_2_ nanomotor (MM@MnO_2_-Au-mSiO_2_@Cur) capable of H_2_O_2_-driven propulsion to facilitate BBB penetration and target inflammation. MnO_2_ catalyzes the conversion of H_2_O_2_ to O_2_, which works synergistically with curcumin-loaded SiO_2_ to promote the polarization of M1 macrophages toward M2 macrophages, thereby achieving neuroprotective and anti-inflammatory effects in neurotherapy applications (Fig. 7c, d) [242].

**Fig. 8:**
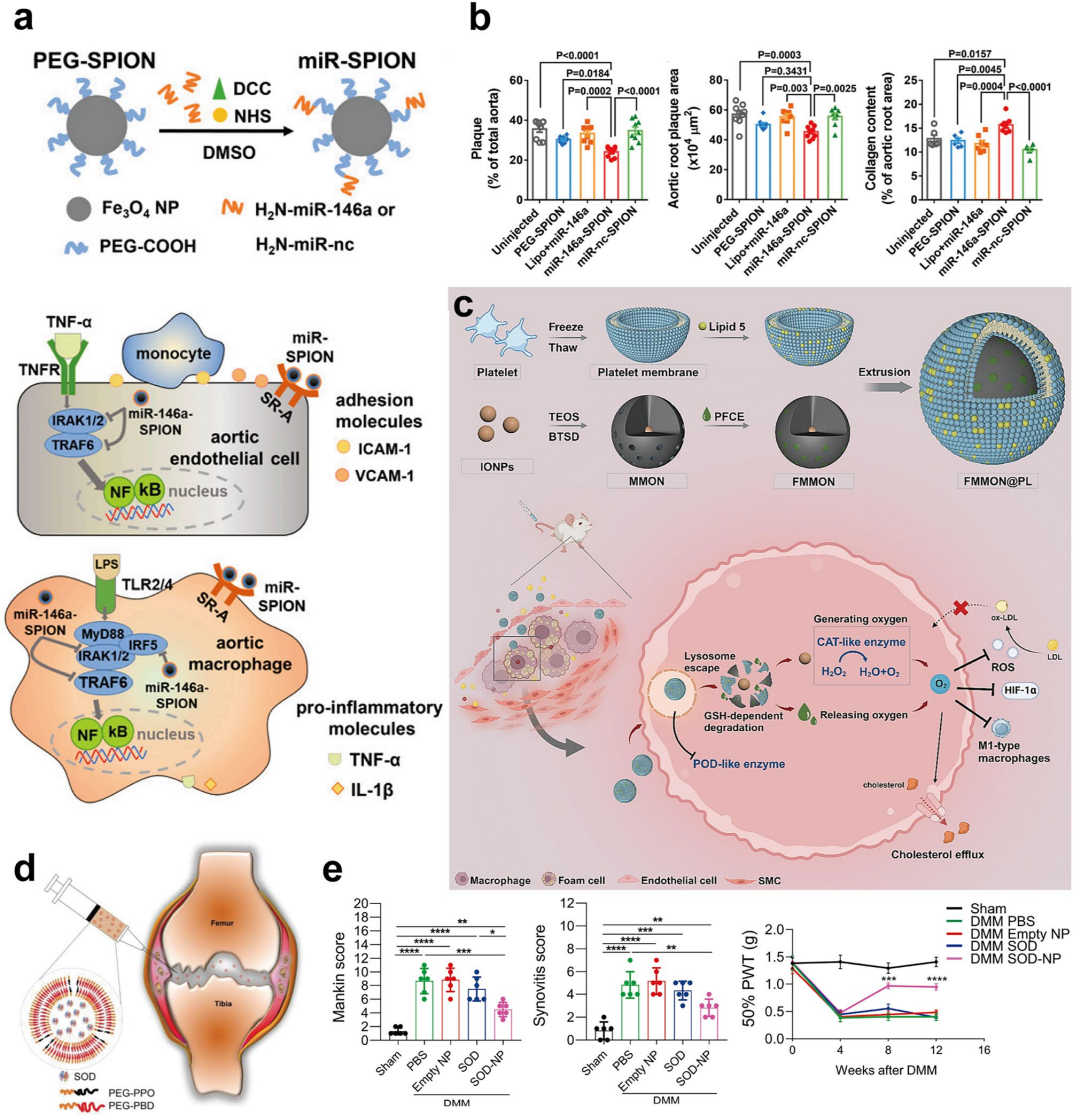
**a** Schematic illustration of the fabrication process and the mechanism of miR-146a-SPIONs. **b** In vivo efficacy of miR-146a-SPIONs for the treatment of AS: fractional plaque area in total aorta, plaque area and collagen content in aortic root [246]. Copyright 2022, National Academy of Sciences. **c** Schematic illustration of the fabrication of FMMON@PL and its use for the therapy of AS [247]. Copyright 2025, Elsevier. **d** Schematic diagram of SOD-loaded polymersomes with high membrane permeability for intra-articular joint injection. **e** Evaluation of therapeutic efficacy of SOD-NP for the treatment of OA: the OA severity of knee joints measured by Mankin score, Synovitis score, and von Frey assay [248]. Copyright 2022, Elsevier

Targeted therapy for AS specifically targets lesion sites, thereby reducing systemic side effects, enhancing efficacy, delaying plaque progression, and lowering cardiovascular risks. Inflammatory endothelial cells and macrophages overexpress VCAM-1/IL-1R, which enables dual targeting via VCAM-1-binding NPs that restore TIMP3 and IL-1R-antagonist systems that promote M2 polarization through rapamycin, resulting in synergistic plaque stabilization and immunomodulation [243–245]. In addition, scavenger receptors (SR-A/CD36) contribute to AS by mediating LDL uptake and foam cell formation. Bai et al*.* developed SR-A-targeted nucleic acid NPs loaded with miR-146a, achieving plaque-specific delivery through scavenger receptor binding. This approach suppresses NF-κB-driven inflammation, leading to the stabilization of AS plaques (Fig. 8a, b) [246]. To address hypoxia in AS, hypoxia-targeted FMMON@PL NPs composed of PFCE/IONPs were designed to deliver oxygen, reduce HIF-1α expression and oxidative stress, and inhibit foam cell/M1 polarization in plaque macrophages. This approach alleviates hypoxia and suppresses AS progression (Fig. 8c) [247].

### Soft Nanomaterials

Soft nanomaterials, including polymeric NPs, nanocellulose-based materials, nanomicelles, liposomes, and exosomes, exhibit excellent biocompatibility and tunable mechanical properties. Compared to rigid materials, soft nanomaterials offer superior biomimetic structural compatibility and reduced mechanical invasiveness, providing efficient, low-toxicity, and intelligent solutions for drug delivery and tissue engineering.

#### Polymeric Materials

**Fig. 9:**
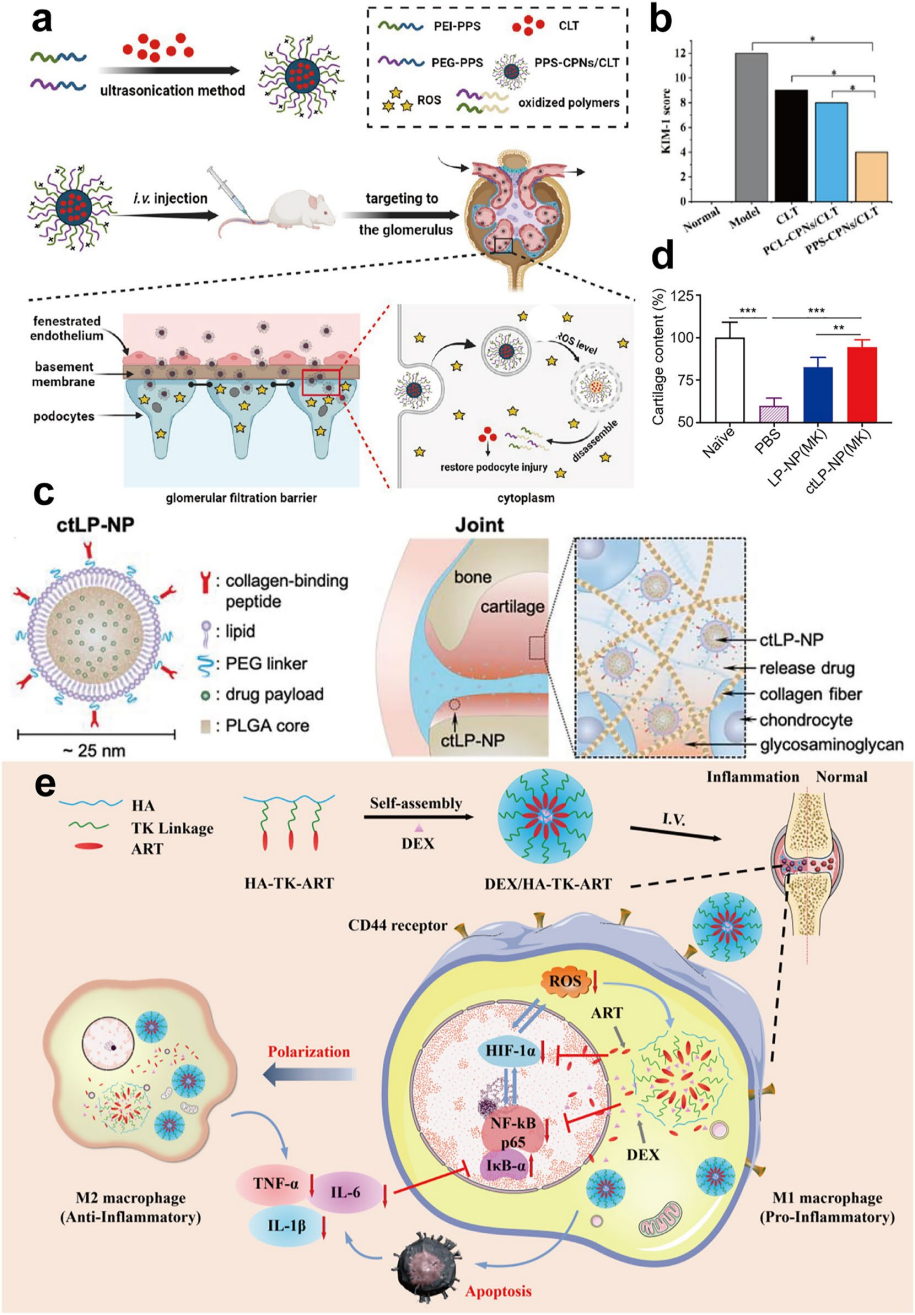
**a** Schematic illustration of the fabrication of ROS-responsive PPS-CPNs/CLT and their glomerulus-targeted delivery fate. **b** In vivo efficacy of PPS-CPNs/CLT for the treatment of membranous nephropathy: semiquantitative scoring of KIM-1 levels, conducted based on immunohistochemical results [250]. Copyright 2024, American Chemical Society. **c** Schematic design of ctLP-NPs containing a core made from PLGA and a shell made from PEG-conjugated lipid. **d** Therapeutic efficacy of MK-8722-loaded ctLP-NPs for repairing cartilage damage in collagenase-induced OA mice: quantification of cartilage content from safranin-O-stained sections (red) [253]. Copyright 2020, John Wiley and Sons. **e** Schematic diagram of ROS-responsive HTA prodrug micelles for co-delivering DEX, inhibiting the HIF-1α/NF-κB cascade to regulate ROS scavenging and macrophage repolarization in RA therapy [262]. Copyright 2022, Elsevier

Polymeric nanomaterials, synthesized through polymerization or self-assembly (e.g., NPs/nanomicelles), provide stable and controlled drug delivery by adjusting molecular weight and modifications. These materials, widely used in medicine, comprise both synthetic polymers (e.g., PLGA, PEG) and natural polymers (e.g., CS), serving as versatile carriers [249]. In the context of immune disease intervention, Gui et al. designed PEG-PPO-doped SOD NPs targeting the synovium. The permeable membrane of these nanoparticles protects SOD while enabling ROS scavenging, inhibiting catabolic enzymes, and alleviating osteoarthritis (OA) (Fig. 8d, e) [248]. Furthermore, the biocompatibility and controlled release properties of polymeric NPs enhance kidney disease treatment. ROS-responsive polymeric NPs (PPS-CPNs/CLT) deliver celastrol to the glomeruli, penetrating endothelial barriers for ROS-triggered drug release in podocytes. This alleviates membranous nephropathy pathologies, such as immune deposits and foot process loss, while reducing toxicity (Fig. 9a, b) [250].

Polymeric NPs act as drug carriers, while natural polymers (e.g., CS, inulin, SF) provide intrinsic anti-inflammatory and antioxidant effects. These natural polymers regulate the gut microbiota, repair mucosal barriers, and directly scavenge ROS, disrupt bacterial DNA, and mitigate inflammation [251]. Their dual role as carriers and bioactive agents advances therapies for IBD, wound healing, and antimicrobial applications, highlighting the versatility of nanomedicine [252]. MCC/CS NPs (mono-carboxyl corrole/CS) self-assemble to exhibit dual antibacterial action, combining electrostatic capture and NIR photothermal effects, thereby accelerating diabetic wound healing [241]. Enhancing drug bioavailability requires functionalized nanomaterials for targeted delivery. In the context of active targeting strategies, Ai et al*.* constructed ultrasmall lipid NPs (LNPs) (ctLP-NPs) with a particle size of less than 30 nm to penetrate the cartilage, where a collagen-binding peptide further facilitated the efficient action of MK-8722 in regulating energy metabolism in chondrocytes and alleviating OA (Fig. 9c, d) [253]. In passive targeting, kidney-targeted mesoscale NPs deliver the ROS scavenger edaravone via the enhanced permeability and retention (EPR) effect, reducing renal cell damage and death, improving kidney function, and preventing chemotherapy-induced kidney injury [254].

Nanocellulose, derived from natural cellulose, offers high strength and biodegradability. Its antibacterial properties enhance infected wound healing in regenerative medicine, as seen with bacterial cellulose (BC) and nanofibers [255, 256]. Li et al*.* introduced a nanocomposite composed of BC and small molecule-decorated Au NPs (BC-Au-DAPT nanocomposites), which inhibited energy metabolism and disrupted bacterial membranes, ultimately combating multidrug-resistant (MDR) Gram-negative bacteria [257]. These effects result from the intrinsic bioactivity of nanocellulose (e.g., natural nanofibers) or from loaded agents (NPs, AMPs). Polysaccharide nanofibers—low toxicity, biocompatibility, and degradability—enable versatile wound healing applications. [258]. For example, cellulose/HA-based nanofibrous dressings mimic the ECM, enhancing cell adhesion, proliferation, and differentiation to promote improved healing [259].

Nanomicelles, similar to liposomes, self-assemble from amphiphilic molecules into normal or reverse structures, encapsulating hydrophobic or hydrophilic drugs. They enhance drug encapsulation, reduce interactions with the body, improve bioavailability, and minimize adverse effects [260]. Vyawahare et al*.* conjugated 9-aminoacridine and caffeic acid to mPEG-PCL nanomicelles (9AA-NMs) to effectively treat RA by inhibiting NF-κB and HIF-1α, mitigating the inflammatory cascade, and ultimately preventing cartilage erosion, swelling, and joint damage [261]. Building on this, the use of micelles in medicine will be further advanced through the incorporation of targeting strategies. DEX/HA-TK-ART micelles co-deliver artesunate and DEX to the joints, synergistically inhibiting HIF-1α/NF-κB and scavenging ROS. The HA targeting and ROS-responsive TK linker help minimize off-target effects (Fig. 9e) [262].

#### Lipid-based Nanomaterials

Lipid-based nanomaterials, including synthetic types like liposomes and solid lipid NPs (SLNs), as well as natural exosomes, feature phospholipid bilayers with aqueous cores for encapsulating antibiotics and anti-inflammatory agents [263]. However, liposomes are susceptible to stability issues from oxidation and hydrolysis, limiting their effectiveness. Surface modifications can improve stability and targeting, thereby enhancing drug delivery to specific sites. For instance, citicoline liposomes coated with chitosan (CT-CS-LPs) promoted diabetic wound healing via sustained release, improved stability, enhanced cellular uptake, and antibacterial effects in rats [264]. SLNs, another type of lipid-based nanosystem, protect drugs from degradation, enhance solubility and bioavailability, enable targeted delivery, and control drug release, making them valuable for the diagnosis and treatment of various diseases [265, 266]. Abudurexiti et al*.* developed cationic SLNs co-loaded with TGF-β1 siRNA and curcumin (siRNA/CUR@SLN) for nasal brain-targeted delivery. This formulation demonstrated anti-inflammatory effects in cerebral hemorrhage models, offering a novel therapy for brain inflammation [267].

**Fig. 10:**
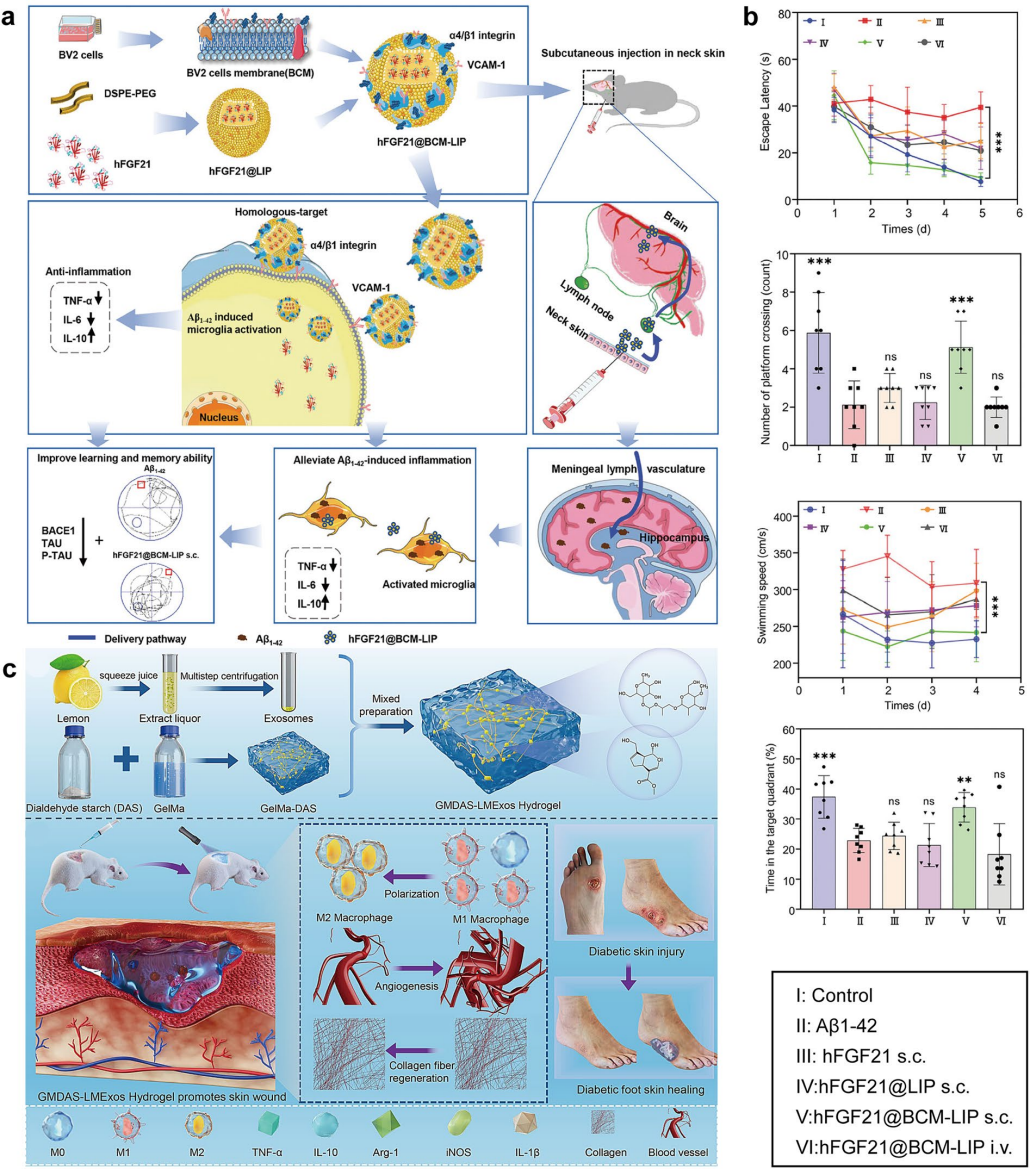
**a** Schematic illustration of biomimetic hFGF21@BCM-LIP for targeted modulation of brain inflammation via the lymphatic system for the treatment of AD. **b** In vivo efficacy of hFGF21@BCM-LIP for the treatment of AD in the Morris water maze, including escape latency, the number of platform crossings, swimming speed and the time spent in the target quadrant [269]. Copyright 2024, John Wiley and Sons. **c** Schematic illustration of the preparation and mechanisms of GelMA/DAS/Exo hydrogel [273]. Copyright 2025, Springer Nature

Regarding targeting design, the strategy aligns with the previously discussed approach, employing stimuli-responsive molecular modifications for targeted functionalization. The lipase-responsive VCM-AS-SLNs target bacterial lipase, reducing MRSA biofilm by five times and eradicating bacteria within 12 h [268]. Unlike conventional approaches, stealth polymers (e.g., PEG) form liposomes that evade phagocytic clearance by macrophages, creating long-circulating systems. These liposomes achieve passive targeting through the EPR effect, enabling specific accumulation at pathological sites. PEGylated liposomes (hFGF21@BCM-LIP) improve the stability of bioactive molecules and promote lymphatic accumulation, targeting activated microglia via VCAM-1/α4β1 to alleviate Aβ-induced cognitive deficits, tau pathology, and neuroinflammation (Fig. 10a, b) [269].

Exosomes, natural lipid nanovesicles with phospholipid/cholesterol bilayers, differ from synthetic carriers (e.g., liposomes) in terms of cellular origin and bioactive cargo (proteins, RNAs). Their biocompatible structure enables intercellular communication through membrane fusion or endocytosis, regulating biological processes [270]. Bio-derived exosomes positively modulate pathologies, e.g., M2b macrophage-derived ones alleviate colitis via CCL1-CCR8 binding and immune activation [271]. Exosomes derived from various MSCs, including human umbilical cord-derived and bone marrow-derived MSCs, can exhibit a protective role against IBD [272]. Plant-derived exosomes, such as those derived from lemon, promote diabetic wound healing by reprogramming macrophages and enhancing cell proliferation. Encapsulated in a GelMA/DAS hydrogel patch (GelMA/DAS/Exo), they enable sustained release and improved penetration, providing a clinical solution for chronic wound repair (Fig. 10c, d) [273]. Furthermore, exosomes can collaborate with active molecules, such as miRNA, to protect genetic information by encapsulating various molecules within a sealed phospholipid bilayer membrane [274, 275].

## Conclusions and Prospects

**Scheme 7:**
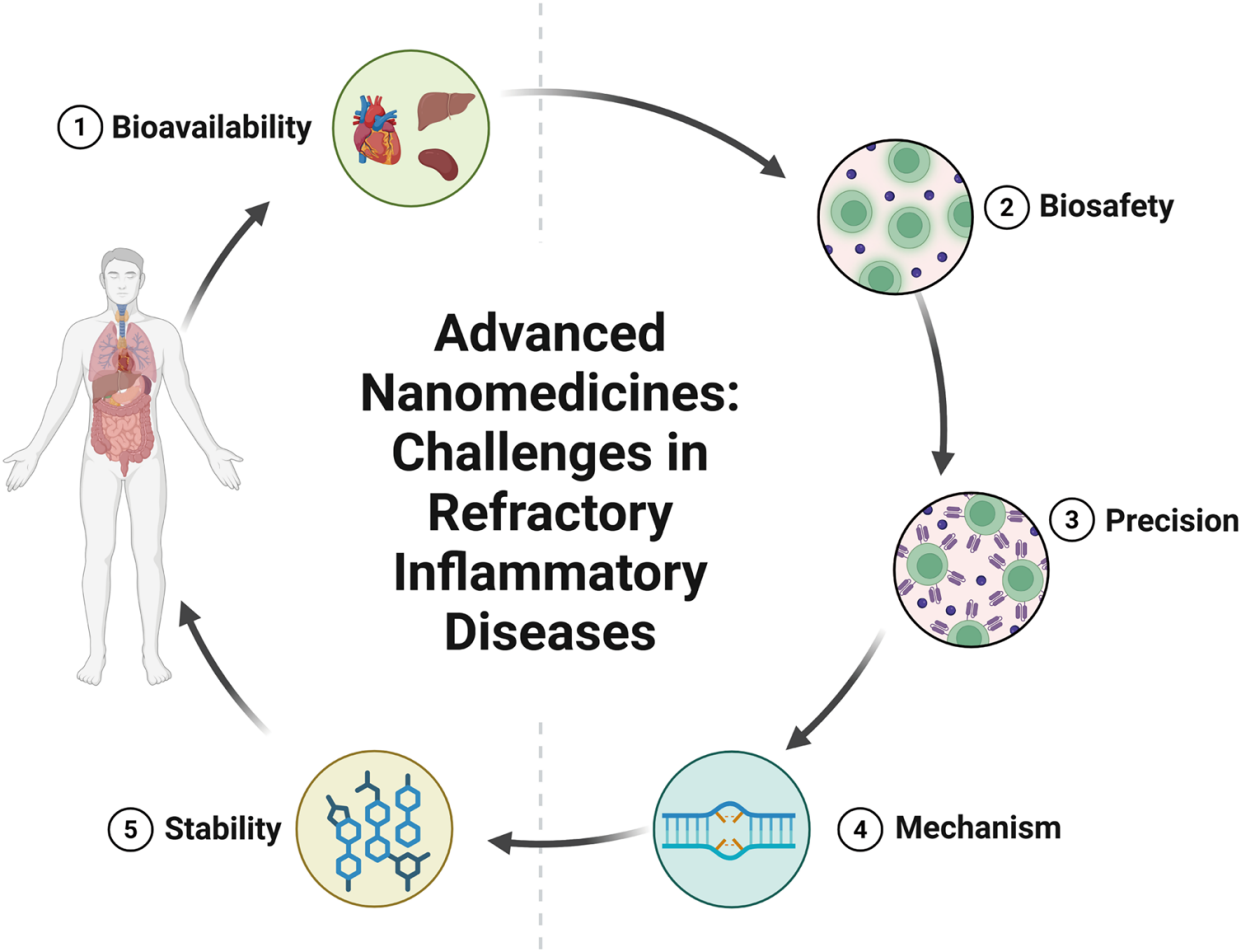
Schematic illustration of the development and challenges of nanomedicine. This diagram encompasses the innovation of delivery routes and drug loading of nanomaterials, the exploration of mechanisms and types of nanozymes, the biosafety of nanomedicine, the clinical application of nanomaterials, and the quest for stable targeting ligands. Created with BioRender.com

This review summarizes the applications of various nanomaterials, including NPs, nanozymes, liposomes, nanofibers, exosomes, and micelles, in treating inflammation-related diseases (Table 1), such as infections, wound healing, liver and kidney injuries, cardiovascular and cerebrovascular diseases, neurological disorders, intestinal inflammation, and rheumatic disorders. These nanomaterials exhibit superior efficacy over traditional small molecule drugs, due to their intrinsic properties or ability to serve as drug carriers, enhancing drug loading capacity and solubility. Specifically, controlling ROS can alleviate inflammation-related oxidative stress through ROS removal. Additionally, this review explores various targeting strategies involving nanomaterials, such as the use of ligands, ROS-responsive materials, or biomimetic biofilms, to modify them. Such modifications can markedly enhance drug bioavailability and boost the therapeutic efficacy of nanomedicines through active targeting (Table 2). The diverse applications discussed offer valuable insights for developing novel nanomedicines and present new opportunities for exploring innovative solutions to prevent and treat diseases. While nanomedicine shows great promise, it is crucial to acknowledge that substantial progress is still required, and the pace of clinical translation remains slow. To expedite the implementation of nanomedicine in clinical settings, the following aspects should be addressed (Scheme 7):

1. Preparation methods are pivotal for the successful clinical translation of nanomedicine. Streamlining the preparation and synthesis processes can save valuable time, reduce costs during the research phase, and ultimately enhance research efficiency. While biosynthesis and self-assembly techniques, as discussed in this paper, enable semi-intelligent synthesis of nanomaterials, these methods still face challenges such as poor stability, high production costs, and difficulties in controlling the synthesis process—issues that impede large-scale production. Therefore, developing efficient, environmentally sustainable synthesis methods that reduce production costs, enhance mass production efficiency, and minimize toxic side effects will be crucial for advancing the clinical application of nanomaterials.
2. Modifying nanomaterials is crucial for enhancing their properties, making them more compatible with the complex physiological environment of the human body. These modifications ensure stability amidst the intricate interactions within the organism and enable nanomedicines to replicate the remarkable therapeutic effects seen in animal studies in vivo. By building upon the original material, nanomaterial modification fosters the creation of diverse, innovative nanomedicines with unique properties, eliminating the need to develop entirely new raw materials. This strategy not only accelerates research but also significantly reduces costs.
3. Expanding the variety and scope of nanomaterial types and applications is crucial for their successful integration into the medical field. The mechanism of action of nanomedicines dictates the extent of their efficacy. For example, nanozymes facilitate the generation or elimination of ROS through mimetic enzyme activities, but the current selection is limited to oxidoreductases, leaving other enzyme types, such as isomerases and cleaving enzymes, largely unexplored. Enzymes differ widely in their catalytic mechanisms, efficiency, and substrate specificity. This variation allows them to be effective under various physiological conditions, thereby expanding the potential applications of nanomedicine. Consequently, continuous exploration of the mechanisms and medical uses of nanomaterials is crucial for offering a broader range of options in nanomedicine.
4. Targeting strategies serve as the “finishing touch” for nanomaterials. The goal of incorporating targeting properties into nanomaterials is to increase their accumulation at the desired site, enhance bioavailability, and ultimately improve efficacy. Conventional passive and active targeting methods, however, encounter several challenges, including uncertainties, lack of repeatability, and safety concerns. For instance, the preparation of biological cell membrane-encapsulated nanoparticles differs significantly from chemical synthesis, and the inherent complexity of biological membranes makes it challenging to ensure that nanodrugs synthesized in different batches consistently contain the same quantities of membrane proteins and targeting properties. Furthermore, the stability of cell membrane-derived nanosystems in vivo poses challenges in assessment and could result in potential toxic side effects. As such, targeting strategies should prioritize minimizing toxicity and ensuring stable performance to enable durable and effective precision therapy with nanomaterials.
5. Choosing the appropriate route of administration is a critical factor in the successful translation of nanomedicine. The significant differences in efficacy between in vitro and in vivo studies can be attributed to anatomical variations between experimental animals and humans. Therefore, it is essential to employ different drug delivery methods that address these differences during research to bridge this gap. At the research stage, comparing the efficacy of various dosage forms and routes is crucial for enhancing the stability, bioavailability, and patient compliance of nanomaterials in the body. Consequently, innovative dosage forms and routes of nanomedicine administration may facilitate their clinical translation.

**Table 1:** Summary of representative nanomaterials for the treatment of inflammatory-related diseases

Disease	Nanomaterials	Strain/Animal model	Main results	References
Infection	Ag NPs; Au NPs; Ag–Au NPs	*K. pneumonia*; *B. subtilis*; *E. coli*; *S. aureus*; *P. aeruginosa*; *Leishmania* major promastigotes	Au-Ag NPs show higher antibacterial activity against *Bacillus subtilis* and *Pseudomonas aeruginosa* than Au NPs and Ag NPs alone	[187]
	CeO_2_ NPs	*E. coli*; *S. aureus*	CeO_2_ NPs undergo a reversible conversion between Ce (III) and Ce (IV), generating ROS that destroy intracellular components and ultimately kill bacteria	[188]
	ZnO@BSA-PEP-MPA	*S. aureus*/*B. subtilis*-infected mouse models	The activity of AMP and ZnO NPs were fully utilized to exert significant targeted antimicrobial effects	[199]
	CeO_2_-GO hybrid nanocomposites	*S. aureus; P. aeruginosin*	The photocatalytic activity exhibits a fivefold enhancement, with synergistic antibacterial effects	[208]
	AMP-Gra (/GO) complex	*E. coli; S. aureus*	Enhanced antibacterial activity 20-fold via synergistic nanosheet interactions and structural optimization	[206]
	Ag-Fe_3_O_4_- SWCNTs	*E. coli; B. megaterium*	Retained dual catalytic/antibacterial activity	[210]
	Pt@V_2_C	S. aureus; MRSA-Induced Cutaneous Abscess Model	59.6% NIR-II photothermal conversion efficiency, synergizing dual enzyme-like PTT/CDT to eradicate MRSA	[212]
	Cu-g-C_3_N_4_/PCL nanofibers	*S. aureus Xen36* multidrug-resistant ESKAPE strains	Eradicates drug-resistant bacteria via dual-enzyme ROS	[229]
	Q-MOF_Ce0.5_	*S. aureus* suspension-infected Kunming mice	Generates sustained ROS under visible light to eradicate surface-adherent bacteria	[231]
	P-CDs	*S. aureus; E. coli*	Generates light-driven ^1^O_2_, eradicating pathogens	[236]
	VCM-AS-SLNs	*S. aureus; *MRSA	Enzyme-responsive; Boost vancomycin’s MRSA efficacy via 100% bacterial clearance	[268]
Wound Healing	Ag-ZnO NPs	Male albino rats with full-thickness skin excision wounds	Antibacterial/antidiabetic activity and CS-enhanced wound healing	[200]
	ZnO NPs	Sprague Dawley (SD) rats with incision wound model	Good bacteriostatic potential against *E. coli* and MRSA, and promotes proliferation of collagen fibers and fibroblasts, reduction of inflammatory cells, and accelerated angiogenesis	[201]
	GO-PEI25k/NO-PEI1.8 k NPs	Male imprinting control region (ICR) mice	NO released by NPs can effectively penetrate the bacterial membrane, and the bactericidal effect is remarkable	[211]
	MCC/CS NPs	MRSA-infected diabetic mice	Selectively targets bacteria through electrostatic interactions; Potent photothermal and inherent antimicrobial synergy against *E. coli* and MRSA; Accelerates wound healing and angiogenesis	[241]
	BC-Au-DAPT	*E. coli/P. aeruginosa-*infected full-thickness wound model in Wistar rats	Combats MDR bacterial infections, accelerates wound healing with superior antibiotic efficacy	[257]
	CT-CS-LPs	Diabetic Wistar rats	Promoting rapid healing of skin wounds in diabetic rats by reducing inflammation, accelerating re-epithelialization, angiogenesis, fibroblast proliferation and connective tissue remodeling	[264]
	GelMA/DAS/Exo hydrogel	SD rats with wound on the back	Promotes diabetic wound healing via macrophage regulation and sustained regeneration	[273]
IBD	YMD@MPDA nanocomposite	Male C57BL/6 mice with DSS-induced colitis	Alleviates IBD via ROS scavenging, immunomodulation, barrier restoration	[217]
	M2b macrophage exosomes	Male BALB/c mice with DSS-induced colitis	The number of regulatory T (Treg) cells and IL-4 levels were increased in the spleens of colitis mice; IL-1β, IL-6 and IL-17A were significantly suppressed; Protection against DSS-induced colitis mediated through the CCL1/CCR8 axis	[271]
	melanin nanozymes	Male C57 BL/6 mice with DSS-induced IBD	Targeted therapeutic effects on IBD are achieved by alleviating oxidative stress, endoplasmic reticulum stress, apoptosis, inflammation, intestinal barrier disruption and intestinal ecological dysregulation	[238]
	M2b exosomes	Mice with DSS-induced colitis	Alleviate colitis via CCL1/CCR8, modulating immunity for IBD therapy	[271]
Rheumatism	iron-quercetin natural coordination NPs (Fe-Qur NCNs)	Female c57/bl/6 mice; Collagen-induced arthritis (CIA) mice; Female DBA mice;	Inhibits activation of NF-κB pathway, scavenges excess ROS, increases phenotype of anti-inflammatory macrophages, alleviates joint swelling, reduces bone erosion and significantly increases bone mass in mice	[194]
	Au-DEN NPs	LPS activated RAW264.7 cells	Targeting; Photothermal properties that enhance ROS production under NIR and improve anti-RA efficacy	[205]
	PtCuOX/CeO_2-X_	Male SD rats with anterior cruciate ligament transection (ACLT)-induced OA	Suppress OA via ROS scavenging, photothermal-enhanced NF-κB pathway inhibition	[220]
	PPBzymes	DMM surgery-induced C57BL/6 male mice OA model	Alleviate OA via JNK phosphorylation blockade and cartilage matrix protection	[225]
	Mn-engineered hollow MSN (MHPH)	Female BALB/c mice with AIA	SOD- and CAT-mimetic enzyme activities; Promotes anti-inflammatory phenotypic differentiation of macrophages; Promotion of biomineralization of bone marrow MCSs	[232]
	MOF nanosheet (ZMTP)	Female Balb/c mice with AIA	Possesses SOD- and CAT-like enzymatic activities; Exerts anti-RA efficacy through anti-inflammatory and pro-biomineralization properties	[233]
	PEG-PPO doped porous polymersomes	C57BL/6 male mice with DMM surgery on the right knee and sham surgery on the left knee	Reduced ROS production and catabolic protease synthesis in articular cartilage and synovium	[248]
	ctLP-NPs	Collagen-induced arthritis male C57BL/6 mice	Delivers AMPK activator, reducing OA degeneration via scalable design	[253]
	9AA-NMs	CIA model in female Wistar rats	Inhibit inflammation, prevent cartilage erosion via NR4A1 in arthritis rat	[261]
	DEX/HA-TK-ART micelles	Male SD rats with ALA	Highly efficient delivery of ART and DEX synergistically inhibited the HIF-1α/NF-κB cascade, scavenging ROS and inducing macrophage repolarization, significantly attenuating joint inflammatory cell infiltration and repairing articular cartilage damage	[262]
Neurological disorders	TAT-CS@Au NPs	–	Good transmembrane ability to inhibit the accumulation of Aβ1-40, antagonize oxidative stress, reduce aberrant tau protein phosphorylation, and inhibit the expression of inflammatory factors	[190]
	L- and D-GSH stabilized gold NPs (L3.3/D3.3)	AD mice	Inhibit Aβ42, cross BBB; D3.3 surpasses L3.3 in AD therapy	[202]
	PM-Au NPs	Parkinson-induced C57BL/6 mice	Alleviate neuroinflammation and enhance motor coordination in Parkinson’s mice	[203]
	MSC-IONPs	5xFAD mice	Promoting the expression of therapeutic molecules in MSCs; Magnetic responsiveness, higher retention efficiency in the brain and enhanced therapeutic effect on AD	[204]
	PEG-MnO_2_ NPs	Male Lewis rats	Penetrate cartilage, scavenge ROS, sustain joint retention, enabling OA chondroprotection	[215]
	CA-Mn_3_O_4_ nanozymes	C57BL/6j mice with 3-nitro propionic acid (3-NPA)-induced Huntington’s disease (HD)	GSH-dependent GPx activity, treating HD via ROS scavenging	[216]
	Q@CeBG nanoreactor	Male C57BL/6 J mice with MPTP-induced PD	Scavenges ROS, reduces neuronal stress, polarizes microglia, enabling PD neuroprotection	[218]
	V_2_C MXenes	SD rats with transient middle cerebral artery occlusion/reperfusion (tMCAO/R)	Alleviates ischemic stroke via ROS scavenging and neuroprotection	[235]
	Synthesis-optimized PEG-cOAC nanozymes	Craniectomy-induced rat traumatic brain injury (TBI) model	Enhance antioxidant activity and restore cerebral perfusion in mild traumatic brain injury models	[237]
	MM@MnO_2_-Au-mSiO_2_@Cur	Controlled Cortical Impact-induced TBI mice model	Target CNS inflammation via BBB penetration, ROS scavenging, and microglia polarization	[242]
	siRNA/CUR@SLN	Type IV collagenase-induced mice intracerebral hemorrhage (ICH)	Enables noninvasive brain targeting, reducing inflammation and enhancing ICH therapy	[267]
	hFGF21@BCM-LIP	Aβ1-42-induced AD mouse model	Targets brain via lymphatics, alleviating AD neuroinflammation and cognitive deficits	[269]
AS	MM@Ce-CDs NPs	Male ApoE^-/-^ mice with fat-rich diets	Enable ROS-targeted theranostics and microenvironment regulation in AS management	[221]
	BSA@PB/Cur nanozyme	Male ApoE^-/-^ mice with fat-rich diets	Alleviate AS via ROS scavenging, inflammation inhibition, and cholesterol efflux	[224]
	V_2_C-PVP Mxene	Male SD rats with MCAO/R	SOD-, POD-, CAT- and GPx-mimetic enzymatic properties; Significantly reduces the size of cerebral infarcts by reducing oxidative stress, inhibiting apoptosis and counteracting inflammatory responses	[235]
	pBAE NPs	Male C57BL/6 mice with left carotid artery ligation	Targets endothelial cells, specifically delivers active ingredient anti-miR-712 to sites of inflammation, reduces high miR-712 expression, and prevents loss of tissue inhibitor of metalloproteinase 3 (TIMP3) in inflamed endothelium	[245]
	miR-146a-SPIONs	ApoE^-/-^ mice with fat- cholesterol diet	Target plaques via SR-A, inhibit NF-κB inflammation, and reduce AS without toxicity	[246]
	FMMON@PL	ApoE^-/-^ mice with fat-rich diets	Alleviates plaque hypoxia, reduces ROS/HIF-1α, inhibits inflammation, and suppresses AS progression	[247]
Liver and Kidney injuries	RBCM@CeO_2_/TAK-242 NPs	C57BL/6J mice with glyoxylate-induced CaOx renal nephrocalcinosis	Suppress oxidative stress, macrophage polarization, targeting kidneys via TLR4/NF-κB pathway	[222]
	MPBZs	Male C57BL/6J mice with APAP-induced liver injury	Alleviate APAP-induced hepatotoxicity via ROS scavenging, Nrf2 activation, and apoptosis inhibition	[226]
	PPS-PEG/PEI NPs	Male SD rats with passive Heymann nephritis	Enhance nephroprotection via glomerular targeting, ensuring efficacy and safety	[250]
	Eda-MNPs	Male C57BL/6 J mice with cisplatin-induced AKI	Reduce renal damage via kidney-selective delivery, enhancing cisplatin therapy	[254]

**Table 2:** Summary of representative nanomaterials applying different targeting strategies

Nanomaterials	Targeting method	Application	References
Au-DEN NPs	Photothermal-guided; MTX	Treatment of RA	[205]
GO-PEI25k/NO-PEI1.8 k NPs	Electrostatic interactions	Antimicrobial therapy	[211]
MM@Ce-CDs NPs	Inflammatory endothelial cell membrane coating	Treatment of AS	[221]
RBCM@CeO_2_/TAK-242 NPs	RBC membrane-coating	Treatment of kidney injury	[222]
MM@MnO_2_-Au-mSiO_2_@Cur	Macrophage membrane-modified	Neuroprotective anti-inflammatory	[242]
pBAE NPs	VHPK	Targeting endothelial cells	[245]
PPS-CPNs/CLT	Polyethylenimine (electrostatic interaction); PEG (stealth functionalization); hydrophobic sulfide moieties (ROS-responsive)	Membranous nephropathy therapy	[250]
PLGA NPs	EPR	Treatment of kidney injury	[254]
Polymeric micelles	HA; Thioketal linker	Treatment of RA	[262]
VCM-AS-SLNs	Ascorbyl stearate (Lipase-responsive)	Antimicrobial therapy	[268]
Liposomes	PEGylated; BV2 membrane-coated	Treatment of AD	[269]

Considering the factors outlined above, it is essential to make significant advancements in nanotechnology and expedite the development of nanomedicine. This progress will ultimately facilitate the successful translation of this emerging field into clinical applications for anti-inflammatory treatment.

## Abbreviations

ROS: Reactive oxygen species
O_2_·^−^: Superoxide anion
H_2_O_2_: Hydrogen peroxide
·OH: Hydroxyl radical
^1^O_2_: Singlet oxygen
AS: Atherosclerosis
PD: Parkinson’s disease
AD: Alzheimer’s disease
IBD: Inflammatory bowel disease
NPs: Nanoparticles
HTS: High-throughput screening
SDT: Sonodynamic therapy
US: Ultrasound
PDT: Photodynamic therapy
PTT: Photothermal therapy
GSH: Glutathione
NIR: Near-infrared
BSA: Bovine serum albumin
*E. coli*: *Escherichia coli*
*S. aureus*: *Staphylococcus aureus*
MRSA: Methicillin-resistant *Staphylococcus aureus*
MSCs: Mesenchymal stem cells
ECM: Extracellular matrix
SF: Silk fibroin
CNTs: Carbon nanotubes
EVs: Extracellular vesicles
ML: Machine learning
AI: Artificial intelligence
SOD: Superoxide dismutase
POD: Peroxidase
OXD: Oxidase
CAT: Catalase
GPx: Glutathione peroxidase
SAzymes: Single-atom nanozymes
AMP: Antimicrobial peptide
MSNs: Mesoporous silica nanoparticles
PB: Prussian blue
ZnO: Zinc oxide nanoparticles
BBB: Blood-brain barrier
IONPs: Iron oxide nanoparticles
GO: Graphene oxide
OA: Osteoarthritis
RA: Rheumatoid arthritis
MOFs: Metal-organic frameworks
CS: Chitosan
PLGA: Poly (lactic-co-glycolic acid)
PEG: Polyethylene glycol
HA: Hyaluronic acid
EPR: Enhanced permeability and retention
BC: Bacterial cellulose
SLNs: Solid lipid nanoparticles

## References

[1] P.D. Kilmer, Nanomedicine. N. Engl. J. Med. **363**, 2434–2443 (2010). 10.1177/146144481036502021158659

[2] C. Zhang, L. Yan, X. Wang, S. Zhu, C. Chen et al., Progress, challenges, and future of nanomedicine. Nano Today **35**, 101008 (2020). 10.1016/j.nantod.2020.101008

[3] D. Nie, C. Liu, M. Yu, X. Jiang, N. Wang et al., Elasticity regulates nanomaterial transport as delivery vehicles: design, characterization, mechanisms and state of the art. Biomaterials **291**, 121879 (2022). 10.1016/j.biomaterials.2022.121879 36343607 10.1016/j.biomaterials.2022.121879

[4] N. Joudeh, D. Linke, Nanoparticle classification, physicochemical properties, characterization, and applications: a comprehensive review for biologists. J. Nanobiotechnology **20**(1), 262 (2022). 10.1186/s12951-022-01477-8 35672712 10.1186/s12951-022-01477-8 PMC9171489

[5] R. Brusini, M. Varna, P. Couvreur, Advanced nanomedicines for the treatment of inflammatory diseases. Adv. Drug Deliv. Rev. **157**, 161–178 (2020). 10.1016/j.addr.2020.07.010 32697950 10.1016/j.addr.2020.07.010 PMC7369016

[6] C.T. Taylor, G. Doherty, P.G. Fallon, E.P. Cummins, The relationship between hypoxia and inflammation. J. Clin. Invest. **126**, 3716–3724 (2016). 10.1172/JCI84433.Cellular 27454299 10.1172/JCI84433 PMC5096820

[7] C. Nathan, A. Ding, Nonresolving inflammation. Cell **140**(6), 871–882 (2010). 10.1016/j.cell.2010.02.029 20303877 10.1016/j.cell.2010.02.029

[8] R. Medzhitov, The spectrum of inflammatory responses. Science **374**(6571), 1070–1075 (2021). 10.1126/science.abi5200 34822279 10.1126/science.abi5200

[9] A.G. Stewart, P.M. Beart, Inflammation: maladies, models, mechanisms and molecules. Br. J. Pharmacol. **173**(4), 631–634 (2016). 10.1111/bph.13389 26847725 10.1111/bph.13389 PMC4742297

[10] Y. Nosaka, A.Y. Nosaka, Generation and detection of reactive oxygen species in photocatalysis. Chem. Rev. **117**(17), 11302–11336 (2017). 10.1021/acs.chemrev.7b00161 28777548 10.1021/acs.chemrev.7b00161

[11] K.M. Holmström, T. Finkel, Cellular mechanisms and physiological consequences of redox-dependent signalling. Nat. Rev. Mol. Cell Biol. **15**(6), 411–421 (2014). 10.1038/nrm3801 24854789 10.1038/nrm3801

[12] K. Dasuri, L. Zhang, J.N. Keller, Oxidative stress, neurodegeneration, and the balance of protein degradation and protein synthesis. Free Radic. Biol. Med. **62**, 170–185 (2013). 10.1016/j.freeradbiomed.2012.09.016 23000246 10.1016/j.freeradbiomed.2012.09.016

[13] A. Görlach, K. Bertram, S. Hudecova, O. Krizanova, Calcium and ROS: a mutual interplay. Redox Biol. **6**, 260–271 (2015). 10.1016/j.redox.2015.08.010 26296072 10.1016/j.redox.2015.08.010 PMC4556774

[14] K. Zhang, R.J. Kaufman, From endoplasmic-reticulum stress to the inflammatory response. Nature **454**(7203), 455–462 (2008). 10.1038/nature07203 18650916 10.1038/nature07203 PMC2727659

[15] C. Martinelli, C. Pucci, M. Battaglini, A. Marino, G. Ciofani, Antioxidants and nanotechnology: promises and limits of potentially disruptive approaches in the treatment of central nervous system diseases. Adv. Healthc. Mater. **9**(3), e1901589 (2020). 10.1002/adhm.201901589 31854132 10.1002/adhm.201901589

[16] C. Kunsch, R.M. Medford, Oxidative stress as a regulator of gene expression in the vasculature. Circ. Res. **85**(8), 753–766 (1999). 10.1161/01.res.85.8.753 10521248 10.1161/01.res.85.8.753

[17] H. Wang, K. Wan, X. Shi, Recent advances in nanozyme research. Adv. Mater. **31**(45), 1805368 (2019). 10.1002/adma.201805368 10.1002/adma.201805368 30589120

[18] M.J. Mitchell, M.M. Billingsley, R.M. Haley, M.E. Wechsler, N.A. Peppas et al., Engineering precision nanoparticles for drug delivery. Nat. Rev. Drug Discov. **20**(2), 101–124 (2021). 10.1038/s41573-020-0090-8 33277608 10.1038/s41573-020-0090-8 PMC7717100

[19] M. Yang, Y. Zhang, Y. Ma, X. Yan, L. Gong et al., Nanoparticle-based therapeutics of inflammatory bowel diseases: a narrative review of the current state and prospects. J. Bio X Res. **3**(4), 157–173 (2020). 10.1097/jbr.0000000000000078

[20] R. Böttger, G. Pauli, P.H. Chao, N. Al Fayez, L. Hohenwarter et al., Lipid-based nanoparticle technologies for liver targeting. Adv. Drug Deliv. Rev. **154–155**, 79–101 (2020). 10.1016/j.addr.2020.06.017 10.1016/j.addr.2020.06.017 32574575

[21] S.S. Liew, X. Qin, J. Zhou, L. Li, W. Huang et al., Smart design of nanomaterials for mitochondria-targeted nanotherapeutics. Angew. Chem. Int. Ed. **60**(5), 2232–2256 (2021). 10.1002/anie.201915826 10.1002/anie.201915826 32128948

[22] W. Poon, B.R. Kingston, B. Ouyang, W. Ngo, W.C.W. Chan, A framework for designing delivery systems. Nat. Nanotechnol. **15**(10), 819–829 (2020). 10.1038/s41565-020-0759-5 32895522 10.1038/s41565-020-0759-5

[23] D.B. Diaz, A.K. Yudin, The versatility of boron in biological target engagement. Nat. Chem. **9**(8), 731–742 (2017). 10.1038/nchem.2814 28754930 10.1038/nchem.2814

[24] W.L.A. Brooks, B.S. Sumerlin, Synthesis and applications of boronic acid-containing polymers: from materials to medicine. Chem. Rev. **116**(3), 1375–1397 (2016). 10.1021/acs.chemrev.5b00300 26367140 10.1021/acs.chemrev.5b00300

[25] Z. Liu, H. He, Synthesis and applications of boronate affinity materials: from class selectivity to biomimetic specificity. Acc. Chem. Res. **50**(9), 2185–2193 (2017). 10.1021/acs.accounts.7b00179 28849912 10.1021/acs.accounts.7b00179

[26] W. Chen, C. Liu, X. Ji, J. Joseph, Z. Tang et al., Stanene-based nanosheets for β-elemene delivery and ultrasound-mediated combination cancer therapy. Angew. Chem. Int. Ed. **60**(13), 7155–7164 (2021). 10.1002/anie.202016330 10.1002/anie.202016330 33434327

[27] Y. Zhang, X. Zhang, H. Yang, L. Yu, Y. Xu et al., Advanced biotechnology-assisted precise sonodynamic therapy. Chem. Soc. Rev. **50**(20), 11227–11248 (2021). 10.1039/d1cs00403d 34661214 10.1039/d1cs00403d

[28] L. Zong, Y. Yu, J. Wang, P. Liu, W. Feng et al., Oxygen-vacancy-rich molybdenum carbide MXene nanonetworks for ultrasound-triggered and capturing-enhanced sonocatalytic bacteria eradication. Biomaterials **296**, 122074 (2023). 10.1016/j.biomaterials.2023.122074 36889145 10.1016/j.biomaterials.2023.122074

[29] D. Cabrera, A. Coene, J. Leliaert, E.J. Artés-Ibáñez, L. Dupré et al., Dynamical magnetic response of iron oxide nanoparticles inside live cells. ACS Nano **12**(3), 2741–2752 (2018). 10.1021/acsnano.7b08995 29508990 10.1021/acsnano.7b08995

[30] H. Van Le, V. Dulong, L. Picton, D. Le Cerf, Thermoresponsive nanogels based on polyelectrolyte complexes between polycations and functionalized hyaluronic acid. Carbohydr. Polym. **292**, 119711 (2022). 10.1016/j.carbpol.2022.119711 35725187 10.1016/j.carbpol.2022.119711

[31] M. He, F. Chen, D. Shao, P. Weis, Z. Wei et al., Photoresponsive metallopolymer nanoparticles for cancer theranostics. Biomaterials **275**, 120915 (2021). 10.1016/j.biomaterials.2021.120915 34102525 10.1016/j.biomaterials.2021.120915

[32] H. Lu, L. Niu, L. Yu, K. Jin, J. Zhang et al., Cancer phototherapy with nano-bacteria biohybrids. J. Control. Release **360**, 133–148 (2023). 10.1016/j.jconrel.2023.06.009 37315693 10.1016/j.jconrel.2023.06.009

[33] R. Guo, S. Wang, L. Zhao, Q. Zong, T. Li et al., Engineered nanomaterials for synergistic photo-immunotherapy. Biomaterials **282**, 121425 (2022). 10.1016/j.biomaterials.2022.121425 35217344 10.1016/j.biomaterials.2022.121425

[34] X. Han, J. Huang, X. Jing, D. Yang, H. Lin et al., Oxygen-deficient black titania for synergistic/enhanced sonodynamic and photoinduced cancer therapy at near infrared-II biowindow. ACS Nano **12**(5), 4545–4555 (2018). 10.1021/acsnano.8b00899 29697960 10.1021/acsnano.8b00899

[35] A. Li, J. Yang, Y. He, J. Wen, X. Jiang, Advancing piezoelectric 2D nanomaterials for applications in drug delivery systems and therapeutic approaches. Nanoscale Horiz. **9**(3), 365–383 (2024). 10.1039/D3NH00578J 38230559 10.1039/d3nh00578j

[36] L. Xia, J. Chen, Y. Xie, S. Zhang, W. Xia et al., Photo-/ piezo-activated ultrathin molybdenum disulfide nanomedicine for synergistic tumor therapy. J. Mater. Chem. B **11**(13), 2895–2903 (2023). 10.1039/d3tb00209h 36919643 10.1039/d3tb00209h

[37] Z. Deng, Y. Qian, Y. Yu, G. Liu, J. Hu et al., Engineering intracellular delivery nanocarriers and nanoreactors from oxidation-responsive polymersomes *via* synchronized bilayer cross-linking and permeabilizing inside live cells. J. Am. Chem. Soc. **138**(33), 10452–10466 (2016). 10.1021/jacs.6b04115 27485779 10.1021/jacs.6b04115

[38] M. Wang, S. Su, X. Zhong, D. Kong, B. Li et al., Enhanced photocatalytic hydrogen production activity by constructing a robust organic-inorganic hybrid material based fulvalene and TiO_2_. Nanomaterials **12**(11), 1918 (2022). 10.3390/nano12111918 35683773 10.3390/nano12111918 PMC9182102

[39] L. Chang, H. Huang, W. Feng, H. Fu, F. Qi et al., Programmed self-assembly of enzyme activity-inhibited nanomedicine for augmenting chemodynamic tumor nanotherapy. Nanoscale **14**(16), 6171–6183 (2022). 10.1039/d2nr00165a 35389406 10.1039/d2nr00165a

[40] F. Wang, H. Duan, R. Zhang, H. Guo, H. Lin et al., Potentiated cytosolic drug delivery and photonic hyperthermia by 2D free-standing silicene nanosheets for tumor nanomedicine. Nanoscale **12**(34), 17931–17946 (2020). 10.1039/D0NR05214K 32845945 10.1039/d0nr05214k

[41] X. Han, J. Huang, H. Lin, Z. Wang, P. Li et al., 2D ultrathin MXene-based drug-delivery nanoplatform for synergistic photothermal ablation and chemotherapy of cancer. Adv. Healthc. Mater. **7**(9), e1701394 (2018). 10.1002/adhm.201701394 29405649 10.1002/adhm.201701394

[42] S. Qiu, X. Wu, Z. Li, X. Xu, J. Wang et al., A smart nanoreactor based on an O_2_-economized dual energy inhibition strategy armed with dual multi-stimuli-responsive doorkeepers for enhanced CDT/PTT of rheumatoid arthritis. ACS Nano **16**(10), 17062–17079 (2022). 10.1021/acsnano.2c07338 36153988 10.1021/acsnano.2c07338

[43] X. Gao, P. Zhu, L. Yu, L. Yang, Y. Chen, Ultrasound/acidity-triggered and nanoparticle-enabled analgesia. Adv. Healthc. Mater. **8**(9), e1801350 (2019). 10.1002/adhm.201801350 30901164 10.1002/adhm.201801350

[44] B.D. Cardoso, V.F. Cardoso, S. Lanceros-Méndez, E.M.S. Castanheira, Solid magnetoliposomes as multi-stimuli-responsive systems for controlled release of doxorubicin: assessment of lipid formulations. Biomedicines **10**(5), 1207 (2022). 10.3390/biomedicines10051207 35625942 10.3390/biomedicines10051207 PMC9138220

[45] R. Zhang, J. Gao, G. Zhao, L. Zhou, F. Kong et al., Tetrazine bioorthogonal chemistry makes nanotechnology a powerful toolbox for biological applications. Nanoscale **15**(2), 461–469 (2023). 10.1039/D2NR06056F 36533721 10.1039/d2nr06056f

[46] C. Wu, J. Xie, Q. Yao, Y. Song, G. Yang et al., Intrahippocampal supramolecular assemblies directed bioorthogonal liberation of neurotransmitters to suppress seizures in freely moving mice. Adv. Mater. **36**(27), 2314310 (2024). 10.1002/adma.202314310 10.1002/adma.202314310 38655719

[47] A.S. Braegelman, M.J. Webber, Integrating stimuli-responsive properties in host-guest supramolecular drug delivery systems. Theranostics **9**(11), 3017–3040 (2019). 10.7150/thno.31913 31244940 10.7150/thno.31913 PMC6567965

[48] Y. Ni, D. Zhang, Y. Wang, X. He, J. He et al., Host-guest interaction-mediated photo/temperature dual-controlled antibacterial surfaces. ACS Appl. Mater. Interfaces **13**(12), 14543–14551 (2021). 10.1021/acsami.0c21626 33733728 10.1021/acsami.0c21626

[49] E. Sameiyan, E. Bagheri, S. Dehghani, M. Ramezani, M. Alibolandi et al., Aptamer-based ATP-responsive delivery systems for cancer diagnosis and treatment. Acta Biomater. **123**, 110–122 (2021). 10.1016/j.actbio.2020.12.057 33453405 10.1016/j.actbio.2020.12.057

[50] E. Esawi, W. Alshaer, I.S. Mahmoud, D.A. Alqudah, B. Azab et al., Aptamer-aptamer *Chimera* for targeted delivery and ATP-responsive release of doxorubicin into cancer cells. Int. J. Mol. Sci. **22**(23), 12940 (2021). 10.3390/ijms222312940 34884745 10.3390/ijms222312940 PMC8657665

[51] Y. Xu, X. Luan, P. He, D. Zhu, R. Mu et al., Fabrication and functional regulation of biomimetic interfaces and their antifouling and antibacterial applications: a review. Small **20**(21), 2308091 (2024). 10.1002/smll.202308091 10.1002/smll.202308091 38088535

[52] K. Tang, J. Xue, Y. Zhu, C. Wu, Design and synthesis of bioinspired nanomaterials for biomedical application. Wires Nanomed. Nanobiotechnol. **16**(1), e1914 (2024). 10.1002/wnan.1914 10.1002/wnan.1914 37394619

[53] M. Imran, V. Gowd, P. Saha, S. Rashid, A. Ahmad Chaudhary et al., Biologically inspired stealth–Camouflaged strategies in nanotechnology for the improved therapies in various diseases. Int. J. Pharm. **631**, 122407 (2023). 10.1016/j.ijpharm.2022.122407 36402290 10.1016/j.ijpharm.2022.122407

[54] G. Zan, Q. Wu, Biomimetic and bioinspired synthesis of nanomaterials/nanostructures. Adv. Mater. **28**(11), 2099–2147 (2016). 10.1002/adma.201503215 26729639 10.1002/adma.201503215

[55] F. Tian, M. Li, S. Wu, L. Li, H. Hu, A hybrid and scalable nanofabrication approach for bio-inspired bactericidal silicon nanospike surfaces. Colloids Surf. B Biointerfaces **222**, 113092 (2023). 10.1016/j.colsurfb.2022.113092 36577343 10.1016/j.colsurfb.2022.113092

[56] S. Wu, F. Zuber, K. Maniura-Weber, J. Brugger, Q. Ren, Nanostructured surface topographies have an effect on bactericidal activity. J. Nanobiotechnology **16**(1), 20 (2018). 10.1186/s12951-018-0347-0 29490703 10.1186/s12951-018-0347-0 PMC5830064

[57] F. Dundar Arisoy, K.W. Kolewe, B. Homyak, I.S. Kurtz, J.D. Schiffman et al., Bioinspired photocatalytic shark-skin surfaces with antibacterial and antifouling activity *via* nanoimprint lithography. ACS Appl. Mater. Interfaces **10**(23), 20055–20063 (2018). 10.1021/acsami.8b05066 29790348 10.1021/acsami.8b05066 PMC6013830

[58] A. Valiei, N. Lin, G. McKay, D. Nguyen, C. Moraes et al., Surface wettability is a key feature in the mechano-bactericidal activity of nanopillars. ACS Appl. Mater. Interfaces **14**(24), 27564–27574 (2022). 10.1021/acsami.2c03258 35670568 10.1021/acsami.2c03258

[59] Y. Chen, J. Gao, J. Ao, J. Zhang, R. Jiang et al., Bioinspired nanoflakes with antifouling and mechano-bactericidal capacity. Colloids Surf. B Biointerfaces **224**, 113229 (2023). 10.1016/j.colsurfb.2023.113229 36863251 10.1016/j.colsurfb.2023.113229

[60] Y. Du, J. Ge, Y. Li, P.X. Ma, B. Lei, Biomimetic elastomeric, conductive and biodegradable polycitrate-based nanocomposites for guiding myogenic differentiation and skeletal muscle regeneration. Biomaterials **157**, 40–50 (2018). 10.1016/j.biomaterials.2017.12.005 29241032 10.1016/j.biomaterials.2017.12.005

[61] P. Shi, N. Zhao, J. Coyne, Y. Wang, DNA-templated synthesis of biomimetic cell wall for nanoencapsulation and protection of mammalian cells. Nat. Commun. **10**(1), 2223 (2019). 10.1038/s41467-019-10231-y 31110174 10.1038/s41467-019-10231-y PMC6527693

[62] Z. Dai, M. Dang, W. Zhang, S. Murugan, S.W. Teh et al., Biomimetic hydroxyapatite/poly xylitol sebacic adibate/vitamin K nanocomposite for enhancing bone regeneration. Artif. Cells Nanomed. Biotechnol. **47**(1), 1898–1907 (2019). 10.1080/21691401.2019.1573183 31066314 10.1080/21691401.2019.1573183

[63] R. Rial, Z. Liu, P. Messina, J.M. Ruso, Role of nanostructured materials in hard tissue engineering. Adv. Colloid Interface Sci. **304**, 102682 (2022). 10.1016/j.cis.2022.102682 35489142 10.1016/j.cis.2022.102682

[64] J.L. Van Eps, J.S. Fernandez-Moure, F.J. Cabrera, F. Taraballi, F. Paradiso et al., Improved posterolateral lumbar spinal fusion using a biomimetic, nanocomposite scaffold augmented by autologous platelet-rich plasma. Front. Bioeng. Biotechnol. **9**, 622099 (2021). 10.3389/fbioe.2021.622099 34485251 10.3389/fbioe.2021.622099 PMC8415153

[65] S. Zhou, J. Xiao, Y. Ji, Y. Feng, S. Yan et al., Natural silk nanofibers as building blocks for biomimetic aerogel scaffolds. Int. J. Biol. Macromol. **237**, 124223 (2023). 10.1016/j.ijbiomac.2023.124223 36996961 10.1016/j.ijbiomac.2023.124223

[66] Y. Wang, X. Yuan, K. Yu, H. Meng, Y. Zheng et al., Fabrication of nanofibrous microcarriers mimicking extracellular matrix for functional microtissue formation and cartilage regeneration. Biomaterials **171**, 118–132 (2018). 10.1016/j.biomaterials.2018.04.033 29684676 10.1016/j.biomaterials.2018.04.033

[67] E. Nazarzadeh Zare, D. Khorsandi, A. Zarepour, H. Yilmaz, T. Agarwal et al., Biomedical applications of engineered heparin-based materials. Bioact. Mater. **31**, 87–118 (2024). 10.1016/j.bioactmat.2023.08.002 37609108 10.1016/j.bioactmat.2023.08.002 PMC10440395

[68] Y. Hao, H. Li, J. Guo, D. Wang, J. Zhang et al., Bio-inspired antioxidant heparin-mimetic peptide hydrogel for radiation-induced skin injury repair. Adv. Healthc. Mater. **12**(20), e2203387 (2023). 10.1002/adhm.202203387 36934301 10.1002/adhm.202203387

[69] T. Tong, W. Tang, S. Xiao, J. Liang, Antiviral effects of heparan sulfate analogue-modified two-dimensional MXene nanocomposites on PRRSV and SARS-CoV-2. Adv. NanoBiomed Res. **2**(10), 2200067 (2022). 10.1002/anbr.202200067 36249178 10.1002/anbr.202200067 PMC9538433

[70] Y. Chen, R. Wang, Y. Wang, W. Zhao, S. Sun et al., Heparin-mimetic polyurethane hydrogels with anticoagulant, tunable mechanical property and controllable drug releasing behavior. Int. J. Biol. Macromol. **98**, 1–11 (2017). 10.1016/j.ijbiomac.2017.01.102 28130129 10.1016/j.ijbiomac.2017.01.102

[71] B. Akgul, C. Gulcan, S. Tornaci, M. Erginer, E. Toksoy Oner et al., Manufacturing radially aligned PCL nanofibers reinforced with sulfated levan and evaluation of its biological activity for healing tympanic membrane perforations. Macromol. Biosci. **25**(1), 2400291 (2025). 10.1002/mabi.202400291 39461894 10.1002/mabi.202400291 PMC11727819

[72] C. Li, M. Zhang, X. Liu, W. Zhao, C. Zhao, Immobilization of heparin-mimetic biomacromolecules on Fe_3_O_4_ nanoparticles as magnetic anticoagulant *via* mussel-inspired coating. Mater. Sci. Eng. C **109**, 110516 (2020). 10.1016/j.msec.2019.110516 10.1016/j.msec.2019.110516 32228930

[73] L. Wang, Y. Wu, T. Hu, P.X. Ma, B. Guo, Aligned conductive core-shell biomimetic scaffolds based on nanofiber yarns/hydrogel for enhanced 3D neurite outgrowth alignment and elongation. Acta Biomater. **96**, 175–187 (2019). 10.1016/j.actbio.2019.06.035 31260823 10.1016/j.actbio.2019.06.035

[74] C. Wu, Y. Sun, X. He, W. Weng, K. Cheng et al., Photothermal extracellular matrix based nanocomposite films and their effect on the osteogenic differentiation of BMSCs. Nanoscale **15**(11), 5379–5390 (2023). 10.1039/D2NR05889H 36825767 10.1039/d2nr05889h

[75] M. Li, Q. Guo, C. Zhong, Z. Zhang, Multifunctional cell membranes-based nano-carriers for targeted therapies: a review of recent trends and future perspective. Drug Deliv. **30**(1), 2288797 (2023). 10.1080/10717544.2023.2288797 38069500 10.1080/10717544.2023.2288797 PMC10987056

[76] P. Dash, A.M. Piras, M. Dash, Cell membrane coated nanocarriers - an efficient biomimetic platform for targeted therapy. J. Control. Release **327**, 546–570 (2020). 10.1016/j.jconrel.2020.09.012 32911013 10.1016/j.jconrel.2020.09.012

[77] Y. Zhao, A. Li, L. Jiang, Y. Gu, J. Liu, Hybrid membrane-coated biomimetic nanoparticles (HM@BNPs): a multifunctional nanomaterial for biomedical applications. Biomacromol **22**(8), 3149–3167 (2021). 10.1021/acs.biomac.1c00440 10.1021/acs.biomac.1c00440 34225451

[78] Q. Tan, L. He, X. Meng, W. Wang, H. Pan et al., Macrophage biomimetic nanocarriers for anti-inflammation and targeted antiviral treatment in COVID-19. J. Nanobiotechnology **19**(1), 173 (2021). 10.1186/s12951-021-00926-0 34112203 10.1186/s12951-021-00926-0 PMC8190731

[79] H. Chen, J. Deng, X. Yao, Y. He, H. Li et al., Bone-targeted erythrocyte-cancer hybrid membrane-camouflaged nanoparticles for enhancing photothermal and hypoxia-activated chemotherapy of bone invasion by OSCC. J. Nanobiotechnology **19**(1), 342 (2021). 10.1186/s12951-021-01088-9 34702291 10.1186/s12951-021-01088-9 PMC8549398

[80] C. Montis, A. Salvatore, F. Valle, L. Paolini, F. Carlà et al., Biogenic supported lipid bilayers as a tool to investigate nano-bio interfaces. J. Colloid Interface Sci. **570**, 340–349 (2020). 10.1016/j.jcis.2020.03.014 32171928 10.1016/j.jcis.2020.03.014

[81] L. Chen, W. Hong, W. Ren, T. Xu, Z. Qian et al., Recent progress in targeted delivery vectors based on biomimetic nanoparticles. Signal Transduct. Target. Ther. **6**(1), 225 (2021). 10.1038/s41392-021-00631-2 34099630 10.1038/s41392-021-00631-2 PMC8182741

[82] Y. Zhang, M. Xiong, X. Ni, J. Wang, H. Rong et al., Virus-mimicking mesoporous silica nanoparticles with an electrically neutral and hydrophilic surface to improve the oral absorption of insulin by breaking through dual barriers of the mucus layer and the intestinal epithelium. ACS Appl. Mater. Interfaces **13**(15), 18077–18088 (2021). 10.1021/acsami.1c00580 33830730 10.1021/acsami.1c00580

[83] Y. Gao, Y. Zhang, H. Xia, Y. Ren, H. Zhang et al., Biomimetic virus-like mesoporous silica nanoparticles improved cellular internalization for co-delivery of antigen and agonist to enhance Tumor immunotherapy. Drug Deliv. **30**(1), 2183814 (2023). 10.1080/10717544.2023.2183814 36843529 10.1080/10717544.2023.2183814 PMC9980018

[84] X. Zhao, Y. Wang, W. Jiang, Q. Wang, J. Li et al., Herpesvirus-mimicking DNAzyme-loaded nanoparticles as a mitochondrial DNA stress inducer to activate innate immunity for tumor therapy. Adv. Mater. **34**(37), 2204585 (2022). 10.1002/adma.202204585 10.1002/adma.202204585 35869026

[85] Z. Wang, J. Wu, J.-J. Zheng, X. Shen, L. Yan et al., Accelerated discovery of superoxide-dismutase nanozymes *via* high-throughput computational screening. Nat. Commun. **12**(1), 6866 (2021). 10.1038/s41467-021-27194-8 34824234 10.1038/s41467-021-27194-8 PMC8616946

[86] X. Qi, J. Pfaendtner, High-throughput computational screening of solid-binding peptides. J. Chem. Theory Comput. **20**(7), 2959–2968 (2024). 10.1021/acs.jctc.3c01286 38499981 10.1021/acs.jctc.3c01286

[87] J.O. Winter, High-throughput tool uncovers links between cell signaling and nanomaterial uptake. Science **377**(6604), 371–372 (2022). 10.1126/science.add3666 35862545 10.1126/science.add3666

[88] G. Perini, E. Rosa, G. Friggeri, L. Di Pietro, M. Barba et al., INSIDIA 20 high-throughput analysis of 3D cancer models: multiparametric quantification of graphene quantum dots photothermal therapy for glioblastoma and pancreatic cancer. Int. J. Mol. Sci. **23**(6), 3217 (2022). 10.3390/ijms23063217 35328638 10.3390/ijms23063217 PMC8948775

[89] J. Peng, D. Schwalbe-Koda, K. Akkiraju, T. Xie, L. Giordano et al., Human- and machine-centred designs of molecules and materials for sustainability and decarbonization. Nat. Rev. Mater. **7**(12), 991–1009 (2022). 10.1038/s41578-022-00466-5

[90] C. Chen, Z. Yaari, E. Apfelbaum, P. Grodzinski, Y. Shamay et al., Merging data curation and machine learning to improve nanomedicines. Adv. Drug Deliv. Rev. **183**, 114172 (2022). 10.1016/j.addr.2022.114172 35189266 10.1016/j.addr.2022.114172 PMC9233944

[91] S. Dhoble, T.-H. Wu, Kenry, Decoding nanomaterial-biosystem interactions through machine learning. Angew. Chem. Int. Ed. **63**(16), e202318380 (2024). 10.1002/anie.202318380 10.1002/anie.202318380 38687554

[92] M. Saeedimasine, R. Rahmani, A.P. Lyubartsev, Biomolecular adsorption on nanomaterials: combining molecular simulations with machine learning. J. Chem. Inf. Model. **64**(9), 3799–3811 (2024). 10.1021/acs.jcim.3c01606 38623916 10.1021/acs.jcim.3c01606 PMC11094735

[93] O.M. Fahmy, R.A. Eissa, H.H. Mohamed, N.G. Eissa, M. Elsabahy, Machine learning algorithms for prediction of entrapment efficiency in nanomaterials. Methods **218**, 133–140 (2023). 10.1016/j.ymeth.2023.08.008 37595853 10.1016/j.ymeth.2023.08.008

[94] N. Serov, V. Vinogradov, Artificial intelligence to bring nanomedicine to life. Adv. Drug Deliv. Rev. **184**, 114194 (2022). 10.1016/j.addr.2022.114194 35283223 10.1016/j.addr.2022.114194

[95] L. Sun, H. Liu, Y. Ye, Y. Lei, R. Islam et al., Smart nanoparticles for cancer therapy. Signal Transduct. Target. Ther. **8**, 418 (2023). 10.1038/s41392-023-01642-x 37919282 10.1038/s41392-023-01642-x PMC10622502

[96] L. Nuhn, Artificial intelligence assists nanoparticles to enter solid tumours. Nat. Nanotechnol. **18**(6), 550–551 (2023). 10.1038/s41565-023-01382-7 37081083 10.1038/s41565-023-01382-7

[97] Z. Lin, W.-C. Chou, Y.-H. Cheng, C. He, N.A. Monteiro-Riviere et al., Predicting nanoparticle delivery to tumors using machine learning and artificial intelligence approaches. Int. J. Nanomed. **17**, 1365–1379 (2022). 10.2147/IJN.S344208 10.2147/IJN.S344208 PMC8961007 35360005

[98] W.-C. Chou, Q. Chen, L. Yuan, Y.-H. Cheng, C. He et al., An artificial intelligence-assisted physiologically-based pharmacokinetic model to predict nanoparticle delivery to tumors in mice. J. Control. Release **361**, 53–63 (2023). 10.1016/j.jconrel.2023.07.040 37499908 10.1016/j.jconrel.2023.07.040 PMC11008607

[99] Y. Yang, G.I.N. Waterhouse, Y. Chen, D. Sun-Waterhouse, D. Li, Microbial-enabled green biosynthesis of nanomaterials: current status and future prospects. Biotechnol. Adv. **55**, 107914 (2022). 10.1016/j.biotechadv.2022.107914 35085761 10.1016/j.biotechadv.2022.107914

[100] M.A. Ali, T. Ahmed, W. Wu, A. Hossain, R. Hafeez et al., Advancements in plant and microbe-based synthesis of metallic nanoparticles and their antimicrobial activity against plant pathogens. Nanomaterials **10**(6), 1146 (2020). 10.3390/nano10061146 32545239 10.3390/nano10061146 PMC7353409

[101] R. Singh, U.U. Shedbalkar, S.A. Wadhwani, B.A. Chopade, Bacteriagenic silver nanoparticles: synthesis, mechanism, and applications. Appl. Microbiol. Biotechnol. **99**(11), 4579–4593 (2015). 10.1007/s00253-015-6622-1 25952110 10.1007/s00253-015-6622-1

[102] D. Gupta, A. Boora, A. Thakur, T.K. Gupta, Green and sustainable synthesis of nanomaterials: recent advancements and limitations. Environ. Res. **231**, 116316 (2023). 10.1016/j.envres.2023.116316 37270084 10.1016/j.envres.2023.116316

[103] M. Šebesta, H. Vojtková, V. Cyprichová, A.P. Ingle, M. Urík et al., Mycosynthesis of metal-containing nanoparticles-synthesis by ascomycetes and basidiomycetes and their application. Int. J. Mol. Sci. **24**(1), 304 (2022). 10.3390/ijms24010304 36613746 10.3390/ijms24010304 PMC9820721

[104] K. Vahabi, G.A. Mansoori, S. Karimi, Biosynthesis of silver nanoparticles by fungus *Trichoderma reesei* (a route for large-scale production of AgNPs). Insciences J. **1**(1), 65–79 (2011). 10.5640/insc.010165

[105] L. Zou, F. Zhu, Z.-E. Long, Y. Huang, Bacterial extracellular electron transfer: a powerful route to the green biosynthesis of inorganic nanomaterials for multifunctional applications. J. Nanobiotechnol. **19**(1), 120 (2021). 10.1186/s12951-021-00868-7 10.1186/s12951-021-00868-7 PMC8077780 33906693

[106] M. Ríos-Silva, M. Pérez, R. Luraschi, E. Vargas, C. Silva-Andrade et al., Anaerobiosis favors biosynthesis of single and multi-element nanostructures. PLoS ONE **17**(10), e0273392 (2022). 10.1371/journal.pone.0273392 36206251 10.1371/journal.pone.0273392 PMC9543976

[107] Y. Yang, K. Yang, J. Wang, D. Cui, M. Zhao, Fabrication and characterization of CdS nanowires templated in tobacco mosaic virus with improved photocatalytic ability. Appl. Microbiol. Biotechnol. **105**(21–22), 8255–8264 (2021). 10.1007/s00253-021-11596-1 34599676 10.1007/s00253-021-11596-1

[108] Y. Wang, T. Douglas, Bioinspired approaches to self-assembly of virus-like particles: from molecules to materials. Acc. Chem. Res. **55**(10), 1349–1359 (2022). 10.1021/acs.accounts.2c00056 35507643 10.1021/acs.accounts.2c00056

[109] H. Zhang, N. Tang, X. Yu, Z. Guo, Z. Liu et al., Natural glycyrrhizic acid-tailored hydrogel with *in situ* gradient reduction of AgNPs layer as high-performance, multi-functional, sustainable flexible sensors. Chem. Eng. J. **430**, 132779 (2022). 10.1016/j.cej.2021.132779

[110] S.M. Reddy, S.B. Karmankar, H.A. Alzahrani, A. Hadap, A. Iqbal et al., Bioinspired synthesis of zinc molybdate nanoparticles: an efficient material for growth inhibition of *Escherichia coli*, *Staphylococcus aureus*, and dye remediation. Bioinorg. Chem. Appl. **2023**, 1287325 (2023). 10.1155/2023/1287325 38623482 10.1155/2023/1287325 PMC11018371

[111] R. Nishanthi, S. Malathi, S. John Paul, P. Palani, Green synthesis and characterization of bioinspired silver, gold and platinum nanoparticles and evaluation of their synergistic antibacterial activity after combining with different classes of antibiotics. Mater. Sci. Eng. C **96**, 693–707 (2019). 10.1016/j.msec.2018.11.050 10.1016/j.msec.2018.11.050 30606583

[112] Y. Abdallah, M. Liu, S.O. Ogunyemi, T. Ahmed, H. Fouad et al., Bioinspired green synthesis of chitosan and zinc oxide nanoparticles with strong antibacterial activity against rice pathogen *Xanthomonas oryzae *pv*. oryzae*. Molecules **25**(20), 4795 (2020). 10.3390/molecules25204795 33086640 10.3390/molecules25204795 PMC7587532

[113] H. Ji, Q. Zhu, Application of intelligent responsive DNA self-assembling nanomaterials in drug delivery. J. Control. Release **361**, 803–818 (2023). 10.1016/j.jconrel.2023.08.036 37597810 10.1016/j.jconrel.2023.08.036

[114] X. Luan, H. Kong, P. He, G. Yang, D. Zhu et al., Self-assembled peptide-based nanodrugs: molecular design, synthesis, functionalization, and targeted tumor bioimaging and biotherapy. Small **19**(3), 2205787 (2023). 10.1002/smll.202205787 10.1002/smll.202205787 36440657

[115] T. Wang, C. Ménard-Moyon, A. Bianco, Self-assembly of amphiphilic amino acid derivatives for biomedical applications. Chem. Soc. Rev. **51**(9), 3535–3560 (2022). 10.1039/d1cs01064f 35412536 10.1039/d1cs01064f

[116] A. Olshefsky, C. Richardson, S.H. Pun, N.P. King, Engineering self-assembling protein nanoparticles for therapeutic delivery. Bioconjug. Chem. **33**(11), 2018–2034 (2022). 10.1021/acs.bioconjchem.2c00030 35487503 10.1021/acs.bioconjchem.2c00030 PMC9673152

[117] M.P. Vincent, J.O. Navidzadeh, S. Bobbala, E.A. Scott, Leveraging self-assembled nanobiomaterials for improved cancer immunotherapy. Cancer Cell **40**(3), 255–276 (2022). 10.1016/j.ccell.2022.01.006 35148814 10.1016/j.ccell.2022.01.006 PMC8930620

[118] B. Li, Y. Cui, X. Wang, R. Tang, Novel nanomaterial–organism hybrids with biomedical potential. Wires Nanomed. Nanobiotechnol. **13**(5), e1706 (2021). 10.1002/wnan.1706 10.1002/wnan.1706 33644977

[119] D. Athanasiadou, K.M.M. Carneiro, DNA nanostructures as templates for biomineralization. Nat. Rev. Chem. **5**(2), 93–108 (2021). 10.1038/s41570-020-00242-5 37117611 10.1038/s41570-020-00242-5

[120] Y. Shang, N. Li, S. Liu, L. Wang, Z.-G. Wang et al., Site-specific synthesis of silica nanostructures on DNA origami templates. Adv. Mater. **32**(21), e2000294 (2020). 10.1002/adma.202000294 32301202 10.1002/adma.202000294

[121] N. Zhao, Z. Zeng, Y. Zu, Self-assembled aptamer-nanomedicine for targeted chemotherapy and gene therapy. Small **14**(4), 201702103 (2018). 10.1002/smll.201702103 10.1002/smll.201702103 PMC5857619 29205808

[122] A.R. Voet, J.R. Tame, Protein-templated synthesis of metal-based nanomaterials. Curr. Opin. Biotechnol. **46**, 14–19 (2017). 10.1016/j.copbio.2016.10.015 28088099 10.1016/j.copbio.2016.10.015

[123] R.J. Wilson, Y. Hui, A.K. Whittaker, C.-X. Zhao, Facile bioinspired synthesis of iron oxide encapsulating silica nanocapsules. J. Colloid Interface Sci. **601**, 78–84 (2021). 10.1016/j.jcis.2021.05.021 34058554 10.1016/j.jcis.2021.05.021

[124] T. Yin, Y. Li, K. Bian, R. Zhu, Z. Liu et al., Self-assembly synthesis of vapreotide-gold hybrid nanoflower for photothermal antitumor activity. Mater. Sci. Eng. C Mater. Biol. Appl. **93**, 716–723 (2018). 10.1016/j.msec.2018.08.017 30274105 10.1016/j.msec.2018.08.017

[125] Y.-Y. Xie, X.-T. Qin, J. Zhang, M.-Y. Sun, F.-P. Wang et al., Self-assembly of peptide nanofibers with chirality-encoded antimicrobial activity. J. Colloid Interface Sci. **622**, 135–146 (2022). 10.1016/j.jcis.2022.04.058 35490617 10.1016/j.jcis.2022.04.058

[126] J. Liu, F. Peng, Y. Kang, D. Gong, J. Fan et al., High-loading self-assembling peptide nanoparticles as a lipid-free carrier for hydrophobic general anesthetics. Int. J. Nanomedicine **16**, 5317–5331 (2021). 10.2147/IJN.S315310 34408412 10.2147/IJN.S315310 PMC8364852

[127] T. Wang, Z. Gao, Y. Zhang, Y. Hong, Y. Tang et al., A supramolecular self-assembled nanomaterial for synergistic therapy of immunosuppressive tumor. J. Control. Release **351**, 272–283 (2022). 10.1016/j.jconrel.2022.09.018 36116581 10.1016/j.jconrel.2022.09.018

[128] X. Zhang, M. Wang, J. Feng, B. Qin, C. Zhang et al., Multifunctional nanoparticles co-loaded with Adriamycin and MDR-targeting siRNAs for treatment of chemotherapy-resistant esophageal cancer. J. Nanobiotechnology **20**(1), 166 (2022). 10.1186/s12951-022-01377-x 35346194 10.1186/s12951-022-01377-x PMC8962182

[129] S. Yang, C. Wang, J. Zhu, C. Lu, H. Li et al., Self-assembling peptide hydrogels functionalized with LN- and BDNF- mimicking epitopes synergistically enhance peripheral nerve regeneration. Theranostics **10**(18), 8227–8249 (2020). 10.7150/thno.44276 32724468 10.7150/thno.44276 PMC7381722

[130] J.L. Chen, C.N. Fries, S.J. Berendam, N.S. Rodgers, E.F. Roe et al., Self-assembling peptide nanofiber HIV vaccine elicits robust vaccine-induced antibody functions and modulates Fc glycosylation. Sci. Adv. **8**(38), eabq0273 (2022). 10.1126/sciadv.abq0273 36149967 10.1126/sciadv.abq0273 PMC9506727

[131] M. Grzelczak, L.M. Liz-Marzán, R. Klajn, Stimuli-responsive self-assembly of nanoparticles. Chem. Soc. Rev. **48**(5), 1342–1361 (2019). 10.1039/c8cs00787j 30688963 10.1039/c8cs00787j

[132] K. Ganguly, D.K. Patel, S.D. Dutta, W.-C. Shin, K.-T. Lim, Stimuli-responsive self-assembly of cellulose nanocrystals (CNCs): Structures, functions, and biomedical applications. Int. J. Biol. Macromol. **155**, 456–469 (2020). 10.1016/j.ijbiomac.2020.03.171 32222290 10.1016/j.ijbiomac.2020.03.171

[133] Y. Zhou, Q. Li, Y. Wu, X. Li, Y. Zhou et al., Molecularly stimuli-responsive self-assembled peptide nanoparticles for targeted imaging and therapy. ACS Nano **17**(9), 8004–8025 (2023). 10.1021/acsnano.3c01452 37079378 10.1021/acsnano.3c01452

[134] W. Zhan, G. Gao, Z. Liu, X. Liu, L. Xu et al., Enzymatic self-assembly of adamantane-peptide conjugate for combating *Staphylococcus aureus* infection. Adv. Healthc. Mater. **12**(18), e2203283 (2023). 10.1002/adhm.202203283 36880480 10.1002/adhm.202203283

[135] A. Vardaxi, S. Pispas, Stimuli-responsive self-assembly of poly(2-(dimethylamino)ethyl methacrylate-co-(oligo ethylene glycol)methacrylate) random copolymers and their modified derivatives. Polymers **15**(6), 1519 (2023). 10.3390/polym15061519 36987299 10.3390/polym15061519 PMC10059824

[136] R. Solano, D. Patiño-Ruiz, L. Tejeda-Benitez, A. Herrera, Metal- and metal/oxide-based engineered nanoparticles and nanostructures: a review on the applications, nanotoxicological effects, and risk control strategies. Environ. Sci. Pollut. Res. Int. **28**(14), 16962–16981 (2021). 10.1007/s11356-021-12996-6 33638785 10.1007/s11356-021-12996-6

[137] L. Sun, R. Zhang, T. Zhang, X. Liu, Y. Zhao et al., Synthesis, applications and biosafety evaluation of carbon dots derived from herbal medicine. Biomed. Mater. **18**(4), 042004 (2023). 10.1088/1748-605X/acdeb8 10.1088/1748-605X/acdeb8 37321231

[138] Y. Cheng, Z. Chen, S. Yang, T. Liu, L. Yin et al., Nanomaterials-induced toxicity on cardiac myocytes and tissues, and emerging toxicity assessment techniques. Sci. Total. Environ. **800**, 149584 (2021). 10.1016/j.scitotenv.2021.149584 34399324 10.1016/j.scitotenv.2021.149584

[139] T. Jiang, Y. Lin, C.A. Amadei, N. Gou, S.M. Rahman et al., Comparative and mechanistic toxicity assessment of structure-dependent toxicity of carbon-based nanomaterials. J. Hazard. Mater. **418**, 126282 (2021). 10.1016/j.jhazmat.2021.126282 34111749 10.1016/j.jhazmat.2021.126282 PMC10631494

[140] K. Djayanti, P. Maharjan, K.H. Cho, S. Jeong, M.S. Kim et al., Mesoporous silica nanoparticles as a potential nanoplatform: therapeutic applications and considerations. Int. J. Mol. Sci. **24**(7), 6349 (2023). 10.3390/ijms24076349 37047329 10.3390/ijms24076349 PMC10094416

[141] L. Xu, Y.-Y. Wang, J. Huang, C.-Y. Chen, Z.-X. Wang et al., Silver nanoparticles: Synthesis, medical applications and biosafety. Theranostics **10**(20), 8996–9031 (2020). 10.7150/thno.45413 32802176 10.7150/thno.45413 PMC7415816

[142] J.T. Buchman, N.V. Hudson-Smith, K.M. Landy, C.L. Haynes, Understanding nanoparticle toxicity mechanisms to inform redesign strategies to reduce environmental impact. Acc. Chem. Res. **52**(6), 1632–1642 (2019). 10.1021/acs.accounts.9b00053 31181913 10.1021/acs.accounts.9b00053

[143] Y. Yao, T. Zhang, M. Tang, The DNA damage potential of quantum dots: Toxicity, mechanism and challenge. Environ. Pollut. **317**, 120676 (2023). 10.1016/j.envpol.2022.120676 36395913 10.1016/j.envpol.2022.120676

[144] A.D. Dey, A. Bigham, Y. Esmaeili, M. Ashrafizadeh, F.D. Moghaddam et al., Dendrimers as nanoscale vectors: unlocking the bars of cancer therapy. Semin. Cancer Biol. **86**(Pt 2), 396–419 (2022). 10.1016/j.semcancer.2022.06.003 35700939 10.1016/j.semcancer.2022.06.003

[145] H. Su, X. Song, J. Li, M.Z. Iqbal, S.S.F. Kenston et al., Biosafety evaluation of Janus Fe_3_O_4_-TiO_2_ nanoparticles in Sprague Dawley rats after intravenous injection. Int. J. Nanomedicine **13**, 6987–7001 (2018). 10.2147/IJN.S167851 30464454 10.2147/IJN.S167851 PMC6217909

[146] X. Liang, M. Tang, Research advances on cytotoxicity of cadmium-containing quantum dots. J. Nanosci. Nanotechnol. **19**(9), 5375–5387 (2019). 10.1166/jnn.2019.16783 30961689 10.1166/jnn.2019.16783

[147] J. Frontiñan-Rubio, E. Llanos-González, V.J. González, E. Vázquez, M. Durán-Prado, Subchronic graphene exposure reshapes skin cell metabolism. J. Proteome Res. **21**(7), 1675–1685 (2022). 10.1021/acs.jproteome.2c00064 35611947 10.1021/acs.jproteome.2c00064 PMC9251767

[148] J. Hou, L. Wang, C. Wang, S. Zhang, H. Liu et al., Toxicity and mechanisms of action of titanium dioxide nanoparticles in living organisms. J. Environ. Sci. **75**, 40–53 (2019). 10.1016/j.jes.2018.06.010 10.1016/j.jes.2018.06.010 30473306

[149] S. Chen, Y. Su, M. Zhang, Y. Zhang, P. Xiu et al., Insights into the toxicological effects of nanomaterials on atherosclerosis: mechanisms involved and influence factors. J. Nanobiotechnology **21**(1), 140 (2023). 10.1186/s12951-023-01899-y 37118804 10.1186/s12951-023-01899-y PMC10148422

[150] H. Yu, Y. Wan, G. Zhang, X. Huang, L. Lin et al., Blood compatibility evaluations of two-dimensional Ti_3_C_2_T _*x*_ nanosheets. Biomed. Mater. **17**(2), 025004 (2022). 10.1088/1748-605X/ac45ed 10.1088/1748-605X/ac45ed 34937009

[151] X. Zhou, W. Jin, H. Sun, C. Li, J. Jia, Perturbation of autophagy: an intrinsic toxicity mechanism of nanoparticles. Sci. Total. Environ. **823**, 153629 (2022). 10.1016/j.scitotenv.2022.153629 35131247 10.1016/j.scitotenv.2022.153629

[152] X. Feng, Y. Zhang, C. Zhang, X. Lai, Y. Zhang et al., Nanomaterial-mediated autophagy: coexisting hazard and health benefits in biomedicine. Part. Fibre Toxicol. **17**(1), 53 (2020). 10.1186/s12989-020-00372-0 33066795 10.1186/s12989-020-00372-0 PMC7565835

[153] J. Zhang, F. Wang, S.S.K. Yalamarty, N. Filipczak, Y. Jin et al., Nano silver-induced toxicity and associated mechanisms. Int. J. Nanomed. **17**, 1851–1864 (2022). 10.2147/IJN.S355131 10.2147/IJN.S355131 PMC9056105 35502235

[154] A. Lérida-Viso, A. Estepa-Fernández, A. García-Fernández, V. Martí-Centelles, R. Martínez-Máñez, Biosafety of mesoporous silica nanoparticles; towards clinical translation. Adv. Drug Deliv. Rev. **201**, 115049 (2023). 10.1016/j.addr.2023.115049 37573951 10.1016/j.addr.2023.115049

[155] N.A. Hanan, H.I. Chiu, M.R. Ramachandran, W.H. Tung, N.N. Mohamad Zain et al., Cytotoxicity of plant-mediated synthesis of metallic nanoparticles: a systematic review. Int. J. Mol. Sci. **19**(6), 1725 (2018). 10.3390/ijms19061725 29891772 10.3390/ijms19061725 PMC6032206

[156] V. Vilas-Boas, M. Vinken, Hepatotoxicity induced by nanomaterials: mechanisms and in vitro models. Arch. Toxicol. **95**(1), 27–52 (2021). 10.1007/s00204-020-02940-x 33155068 10.1007/s00204-020-02940-x

[157] J.G. Croissant, Y. Fatieiev, A. Almalik, N.M. Khashab, Mesoporous silica and organosilica nanoparticles: physical chemistry, biosafety, delivery strategies, and biomedical applications. Adv. Healthc. Mater. **7**(4), 1700831 (2018). 10.1002/adhm.201700831 10.1002/adhm.201700831 29193848

[158] X. Gao, X. Zhang, Y. Wang, C. Fan, Effects of morphology and surface hydroxyl on the toxicity of BiOCl in human HaCaT cells. Chemosphere **163**, 438–445 (2016). 10.1016/j.chemosphere.2016.08.063 27565311 10.1016/j.chemosphere.2016.08.063

[159] Z. Wang, Y. Long, J. Fan, C. Xiao, C. Tong et al., Biosafety and biocompatibility assessment of Prussian blue nanoparticles in vitro and in vivo. Nanomedicine **15**(27), 2655–2670 (2020). 10.2217/nnm-2020-0191 33179590 10.2217/nnm-2020-0191

[160] F. Li, R. Li, F. Lu, L. Xu, L. Gan et al., Adverse effects of silver nanoparticles on aquatic plants and zooplankton: a review. Chemosphere **338**, 139459 (2023). 10.1016/j.chemosphere.2023.139459 37437614 10.1016/j.chemosphere.2023.139459

[161] X. Yang, Z. Wang, J. Xu, C. Zhang, P. Gao et al., Effects of dissolved organic matter on the environmental behavior and toxicity of metal nanomaterials: a review. Chemosphere **358**, 142208 (2024). 10.1016/j.chemosphere.2024.142208 38704042 10.1016/j.chemosphere.2024.142208

[162] J. Zhang, L. Jiang, D. Wu, Y. Yin, H. Guo, Effects of environmental factors on the growth and microcystin production of *Microcystis aeruginosa* under TiO_2_ nanoparticles stress. Sci. Total. Environ. **734**, 139443 (2020). 10.1016/j.scitotenv.2020.139443 32454338 10.1016/j.scitotenv.2020.139443

[163] L. Sun, Y. Sogo, X. Wang, A. Ito, Biosafety of mesoporous silica nanoparticles: a combined experimental and literature study. J. Mater. Sci. Mater. Med. **32**(9), 102 (2021). 10.1007/s10856-021-06582-y 34406531 10.1007/s10856-021-06582-y PMC8373747

[164] M. Hassanpour, S.A. Hosseini Tafreshi, O. Amiri, M. Hamadanian, M. Salavati-Niasari, Toxic effects of Fe_2_WO_6_ nanoparticles towards microalga *Dunaliella salina*: Sonochemical synthesis nanoparticles and investigate its impact on the growth. Chemosphere **258**, 127348 (2020). 10.1016/j.chemosphere.2020.127348 32540542 10.1016/j.chemosphere.2020.127348

[165] G. Wu, Y. Huang, J. Li, Y. Lu, L. Liu et al., Chronic level of exposures to low-dosed MoS_2_ nanomaterials exhibits more toxic effects in HaCaT keratinocytes. Ecotoxicol. Environ. Saf. **242**, 113848 (2022). 10.1016/j.ecoenv.2022.113848 35835073 10.1016/j.ecoenv.2022.113848

[166] M. Canta, V. Cauda, The investigation of the parameters affecting the ZnO nanoparticle cytotoxicity behaviour: a tutorial review. Biomater. Sci. **8**(22), 6157–6174 (2020). 10.1039/D0BM01086C 33079078 10.1039/d0bm01086c PMC7610635

[167] A.A.M. Kämpfer, M. Busch, V. Büttner, G. Bredeck, B. Stahlmecke et al., Model complexity as determining factor for *in vitro* nanosafety studies: effects of silver and titanium dioxide nanomaterials in intestinal models. Small **17**(15), 2004223 (2021). 10.1002/smll.202004223 10.1002/smll.202004223 33458953

[168] M.M. Alsmadi, N.K. Al-Nemrawi, R. Obaidat, A.E. Abu Alkahsi, K.M. Korshed et al., Insights into the mapping of green synthesis conditions for ZnO nanoparticles and their toxicokinetics. Nanomedicine **17**(18), 1281–1303 (2022). 10.2217/nnm-2022-0092 36254841 10.2217/nnm-2022-0092

[169] M. Kus-Liśkiewicz, P. Fickers, I. Ben Tahar, Biocompatibility and cytotoxicity of gold nanoparticles: recent advances in methodologies and regulations. Int. J. Mol. Sci. **22**(20), 10952 (2021). 10.3390/ijms222010952 34681612 10.3390/ijms222010952 PMC8536023

[170] Y. Li, L. Zhong, L. Zhang, X. Shen, L. Kong et al., Research advances on the adverse effects of nanomaterials in a model organism. Caenorhabditis elegans. Environ. Toxicol. Chem. **40**(9), 2406–2424 (2021). 10.1002/etc.5133 34078000 10.1002/etc.5133

[171] Y. Yao, T. Zhang, M. Tang, A critical review of advances in reproductive toxicity of common nanomaterials to *Caenorhabditis elegans* and influencing factors. Environ. Pollut. **306**, 119270 (2022). 10.1016/j.envpol.2022.119270 35398402 10.1016/j.envpol.2022.119270

[172] H.-R. Jia, Y.-X. Zhu, Q.-Y. Duan, Z. Chen, F.-G. Wu, Nanomaterials meet zebrafish: Toxicity evaluation and drug delivery applications. J. Control. Release **311–312**, 301–318 (2019). 10.1016/j.jconrel.2019.08.022 10.1016/j.jconrel.2019.08.022 31446084

[173] I. Guseva Canu, S. Fraize-Frontier, C. Michel, S. Charles, Weight of epidemiological evidence for titanium dioxide risk assessment: current state and further needs. J. Expo. Sci. Environ. Epidemiol. **30**(3), 430–435 (2020). 10.1038/s41370-019-0161-2 31420585 10.1038/s41370-019-0161-2

[174] G. Squillacioti, T. Charreau, P. Wild, V. Bellisario, F. Ghelli et al., Worse pulmonary function in association with cumulative exposure to nanomaterials Hints of a mediation effect *via* pulmonary inflammation. Part. Fibre Toxicol.. Fibre Toxicol. **21**(1), 28 (2024). 10.1186/s12989-024-00589-3 10.1186/s12989-024-00589-3 PMC11212158 38943182

[175] M.R. Miller, C.A. Poland, Nanotoxicology: the need for a human touch? Small **16**(36), e2001516 (2020). 10.1002/smll.202001516 32697439 10.1002/smll.202001516

[176] N. Weng, J. Meng, S. Huo, F. Wu, W.-X. Wang, Hemocytes of bivalve mollusks as cellular models in toxicological studies of metals and metal-based nanomaterials. Environ. Pollut. **312**, 120082 (2022). 10.1016/j.envpol.2022.120082 36057327 10.1016/j.envpol.2022.120082

[177] B. Hu, Z. Cheng, S. Liang, Advantages and prospects of stem cells in nanotoxicology. Chemosphere **291**, 132861 (2022). 10.1016/j.chemosphere.2021.132861 34774913 10.1016/j.chemosphere.2021.132861

[178] C. Yang, Z. Du, L. Mei, X. Chen, Y. Liao et al., Influences of lead-based perovskite nanoparticles exposure on early development of human retina. J. Nanobiotechnol. **23**(1), 144 (2025). 10.1186/s12951-025-03245-w 10.1186/s12951-025-03245-w PMC11863764 40001141

[179] C.D. Abueva, S.R. Yoon, N.T. Carpena, S.C. Ahn, S.Y. Chang et al., Development of NIR photocleavable nanoparticles with BDNF for vestibular neuron regeneration. J. Nanobiotechnol. **23**(1), 209 (2025). 10.1186/s12951-025-03298-x 10.1186/s12951-025-03298-x PMC11905548 40075449

[180] M. Prasad, R. Kumar, L. Buragohain, A. Kumari, M. Ghosh, Organoid technology: a reliable developmental biology tool for organ-specific nanotoxicity evaluation. Front. Cell Dev. Biol. **9**, 696668 (2021). 10.3389/fcell.2021.696668 34631696 10.3389/fcell.2021.696668 PMC8495170

[181] J. Liu, M. Qin, Y. Shi, R. Jiang, Z. Wang et al., Volatile carbonyl metabolites analysis of nanoparticle exposed lung cells in an organ-on-a-chip system. Talanta **274**, 126066 (2024). 10.1016/j.talanta.2024.126066 38599125 10.1016/j.talanta.2024.126066

[182] E. Joossens, P. Macko, T. Palosaari, K. Gerloff, I. Ojea-Jiménez et al., A high throughput imaging database of toxicological effects of nanomaterials tested on HepaRG cells. Sci. Data **6**(1), 46 (2019). 10.1038/s41597-019-0053-2 31048742 10.1038/s41597-019-0053-2 PMC6497662

[183] A.R. Collins, B. Annangi, L. Rubio, R. Marcos, M. Dorn et al., High throughput toxicity screening and intracellular detection of nanomaterials. Wires Nanomed. Nanobiotechnol. **9**(1), e1413 (2017). 10.1002/wnan.1413 10.1002/wnan.1413 PMC5215403 27273980

[184] D.A. Winkler, Role of artificial intelligence and machine learning in nanosafety. Small **16**(36), e2001883 (2020). 10.1002/smll.202001883 32537842 10.1002/smll.202001883

[185] Y. Zhou, Y. Wang, W. Peijnenburg, M.G. Vijver, S. Balraadjsing et al., Using machine learning to predict adverse effects of metallic nanomaterials to various aquatic organisms. Environ. Sci. Technol. **57**(46), 17786–17795 (2023). 10.1021/acs.est.2c07039 36730792 10.1021/acs.est.2c07039

[186] Y. Wang, Y. Yang, Y. Shi, H. Song, C. Yu, Antibiotic-free antibacterial strategies enabled by nanomaterials: progress and perspectives. Adv. Mater. **32**(18), e1904106 (2020). 10.1002/adma.201904106 31799752 10.1002/adma.201904106

[187] B. Abbasi, M. Zaka, S. Hashmi, Z. Khan, Biogenic synthesis of Au, Ag and Au–Ag alloy nanoparticles using *Cannabis sativa* leaf extract. IET Nanobiotechnol. **12**(3), 277–284 (2018). 10.1049/iet-nbt.2017.0169

[188] M. Zhang, C. Zhang, X. Zhai, F. Luo, Y. Du et al., Antibacterial mechanism and activity of cerium oxide nanoparticles. Sci. China Mater. **62**(11), 1727–1739 (2019). 10.1007/s40843-019-9471-7

[189] C. Dunnill, T. Patton, J. Brennan, J. Barrett, M. Dryden et al., Reactive oxygen species (ROS) and wound healing: the functional role of ROS and emerging ROS-modulating technologies for augmentation of the healing process. Int. Wound J. **14**(1), 89–96 (2017). 10.1111/iwj.12557 26688157 10.1111/iwj.12557 PMC7950185

[190] Y. Feng, X. Li, D. Ji, J. Tian, Q. Peng et al., Functionalised penetrating peptide-chondroitin sulphate-gold nanoparticles: Synthesis, characterization, and applications as an anti-Alzheimer’s disease drug. Int. J. Biol. Macromol. **230**, 123125 (2023). 10.1016/j.ijbiomac.2022.123125 36603725 10.1016/j.ijbiomac.2022.123125

[191] D.M. Teleanu, A.-G. Niculescu, I.I. Lungu, C.I. Radu, O. Vladâcenco et al., An overview of oxidative stress, neuroinflammation, and neurodegenerative diseases. Int. J. Mol. Sci. **23**(11), 5938 (2022). 10.3390/ijms23115938 35682615 10.3390/ijms23115938 PMC9180653

[192] A. Misrani, S. Tabassum, L. Yang, Mitochondrial dysfunction and oxidative stress in Alzheimer’s disease. Front. Aging Neurosci. **13**, 617588 (2021). 10.3389/fnagi.2021.617588 33679375 10.3389/fnagi.2021.617588 PMC7930231

[193] J.L.M. Björkegren, A.J. Lusis, Atherosclerosis: recent developments. Cell **185**(10), 1630–1645 (2022). 10.1016/j.cell.2022.04.004 35504280 10.1016/j.cell.2022.04.004 PMC9119695

[194] Z. Han, X. Gao, Y. Wang, S. Cheng, X. Zhong et al., Ultrasmall iron-quercetin metal natural product nano complex with antioxidant and macrophage regulation in rheumatoid arthritis. Acta Pharm. Sin. B **13**(4), 1726–1739 (2023). 10.1016/j.apsb.2022.11.020 37139421 10.1016/j.apsb.2022.11.020 PMC10150182

[195] G. Aviello, U.G. Knaus, ROS in gastrointestinal inflammation: Rescue Or Sabotage? Br. J. Pharmacol. **174**(12), 1704–1718 (2017). 10.1111/bph.13428 26758851 10.1111/bph.13428 PMC5446568

[196] S. Zhang, R. Langer, G. Traverso, Nanoparticulate drug delivery systems targeting inflammation for treatment of inflammatory bowel disease. Nano Today **16**, 82–96 (2017). 10.1016/j.nantod.2017.08.006 31186671 10.1016/j.nantod.2017.08.006 PMC6557461

[197] H. Chen, S. Zhou, M. Zhu, B. Wang, W. Chen et al., Gold nanoparticles modified with polyethyleneimine disturbed the activity of drug-metabolic enzymes and induced inflammation-mediated liver injury in mice. Front. Pharmacol. **12**, 706791 (2021). 10.3389/fphar.2021.706791 34335268 10.3389/fphar.2021.706791 PMC8321413

[198] Y. Yang, S. Fan, Q. Chen, Y. Lu, Y. Zhu et al., Acute exposure to gold nanoparticles aggravates lipopolysaccharide-induced liver injury by amplifying apoptosis *via* ROS-mediated macrophage-hepatocyte crosstalk. J. Nanobiotechnology **20**(1), 37 (2022). 10.1186/s12951-021-01203-w 35057820 10.1186/s12951-021-01203-w PMC8772144

[199] H. Chen, M. Zhang, B. Li, D. Chen, X. Dong et al., Versatile antimicrobial peptide-based ZnO quantum dots for *in vivo* bacteria diagnosis and treatment with high specificity. Biomaterials **53**, 532–544 (2015). 10.1016/j.biomaterials.2015.02.105 25890749 10.1016/j.biomaterials.2015.02.105

[200] Y. Iqbal, A. Raouf Malik, T. Iqbal, M. Hammad Aziz, F. Ahmed et al., Green synthesis of ZnO and Ag-doped ZnO nanoparticles using *Azadirachta indica* leaves: Characterization and their potential antibacterial, antidiabetic, and wound-healing activities. Mater. Lett. **305**, 130671 (2021). 10.1016/j.matlet.2021.130671

[201] M. Irfan, H. Munir, H. Ismail, Characterization and fabrication of zinc oxide nanoparticles by gum *Acacia modesta* through green chemistry and impregnation on surgical sutures to boost up the wound healing process. Int. J. Biol. Macromol. **204**, 466–475 (2022). 10.1016/j.ijbiomac.2022.02.043 35157899 10.1016/j.ijbiomac.2022.02.043

[202] K. Hou, J. Zhao, H. Wang, B. Li, K. Li et al., Chiral gold nanoparticles enantioselectively rescue memory deficits in a mouse model of Alzheimer’s disease. Nat. Commun. **11**(1), 4790 (2020). 10.1038/s41467-020-18525-2 32963242 10.1038/s41467-020-18525-2 PMC7509831

[203] J. Xue, T. Liu, Y. Liu, Y. Jiang, V.D.D. Seshadri et al., Neuroprotective effect of biosynthesised gold nanoparticles synthesised from root extract of *Paeonia moutan* against Parkinson disease - *In vitro* & *In vivo* model. J. Photochem. Photobiol. B. **200**, 111635 (2019). 10.1016/j.jphotobiol.2019.111635 31671372 10.1016/j.jphotobiol.2019.111635

[204] M. Jung, H. Kim, J.W. Hwang, Y. Choi, M. Kang et al., Iron oxide nanoparticle-incorporated mesenchymal stem cells for Alzheimer’s disease treatment. Nano Lett. **23**(2), 476–490 (2023). 10.1021/acs.nanolett.2c03682 36638236 10.1021/acs.nanolett.2c03682

[205] P.K. Pandey, R. Maheshwari, N. Raval, P. Gondaliya, K. Kalia et al., Nanogold-core multifunctional dendrimer for pulsatile chemo-, photothermal- and photodynamic- therapy of rheumatoid arthritis. J. Colloid Interface Sci. **544**, 61–77 (2019). 10.1016/j.jcis.2019.02.073 30825801 10.1016/j.jcis.2019.02.073

[206] X. Lu, J. Liu, L. Gou, J. Li, B. Yuan et al., Designing melittin-graphene hybrid complexes for enhanced antibacterial activity. Adv. Healthc. Mater. **8**(9), e1801521 (2019). 10.1002/adhm.201801521 30866165 10.1002/adhm.201801521

[207] Q. Xin, H. Shah, A. Nawaz, W. Xie, M.Z. Akram et al., Antibacterial carbon-based nanomaterials. Adv. Mater. **31**(45), e1804838 (2019). 10.1002/adma.201804838 30379355 10.1002/adma.201804838

[208] L. Kashinath, K. Namratha, K. Byrappa, Microwave mediated synthesis and characterization of CeO_2_-GO hybrid composite for removal of chromium ions and its antibacterial efficiency. J. Environ. Sci. **76**, 65–79 (2019). 10.1016/j.jes.2018.03.027 10.1016/j.jes.2018.03.027 30528036

[209] N. Dubey, K. Ellepola, F.E.D. Decroix, J.L.P. Morin, A.C. Neto et al., Graphene onto medical grade titanium: an atom-thick multimodal coating that promotes osteoblast maturation and inhibits biofilm formation from distinct species. Nanotoxicology **12**(4), 274–289 (2018). 10.1080/17435390.2018.1434911 29409364 10.1080/17435390.2018.1434911

[210] B. Bhaduri, M. Engel, T. Polubesova, W. Wu, B. Xing et al., Dual functionality of an Ag-Fe_3_O_4_-carbon nanotube composite material: Catalytic reduction and antibacterial activity. J. Environ. Chem. Eng. **6**(4), 4103–4113 (2018). 10.1016/j.jece.2018.06.023

[211] J. Cao, S.P. Hlaing, J. Lee, J. Kim, E.H. Lee et al., Bacteria-adhesive nitric oxide-releasing graphene oxide nanoparticles for MRPA-infected wound healing therapy. ACS Appl. Mater. Interfaces **14**(45), 50507–50519 (2022). 10.1021/acsami.2c13317 36331408 10.1021/acsami.2c13317

[212] X. He, Y. Lv, Y. Lin, H. Yu, Y. Zhang et al., Platinum nanoparticles regulated V2C MXene nanoplatforms with NIR-II enhanced nanozyme effect for photothermal and chemodynamic anti-infective therapy. Adv. Mater. **36**(25), 2400366 (2024). 10.1002/adma.202400366 10.1002/adma.202400366 38469896

[213] F. Attar, M.G. Shahpar, B. Rasti, M. Sharifi, A.A. Saboury et al., Nanozymes with intrinsic peroxidase-like activities. J. Mol. Liq. **278**, 130–144 (2019). 10.1016/j.molliq.2018.12.011

[214] L. Mei, S. Zhu, Y. Liu, W. Yin, Z. Gu et al., An overview of the use of nanozymes in antibacterial applications. Chem. Eng. J. **418**, 129431 (2021). 10.1016/j.cej.2021.129431

[215] S. Kumar, I.M. Adjei, S.B. Brown, O. Liseth, B. Sharma, Manganese dioxide nanoparticles protect cartilage from inflammation-induced oxidative stress. Biomaterials **224**, 119467 (2019). 10.1016/j.biomaterials.2019.119467 31557589 10.1016/j.biomaterials.2019.119467 PMC7025913

[216] A. Adhikari, S. Mondal, M. Das, P. Biswas, U. Pal et al., Incorporation of a biocompatible nanozyme in cellular antioxidant enzyme cascade reverses Huntington’s like disorder in preclinical model. Adv. Healthc. Mater. **10**(7), e2001736 (2021). 10.1002/adhm.202001736 33326181 10.1002/adhm.202001736

[217] X. Zhang, H. Yang, Y. He, D. Zhang, G. Lu et al., Yeast-inspired orally-administered nanocomposite scavenges oxidative stress and restores gut immune homeostasis for inflammatory bowel disease treatment. ACS Nano **19**(7), 7350–7369 (2025). 10.1021/acsnano.4c18099 39943645 10.1021/acsnano.4c18099

[218] Y. Gao, L. Zhai, J. Chen, D. Lin, L.-K. Zhang et al., Focused ultrasound-mediated cerium-based nanoreactor against Parkinson’s disease *via* ROS regulation and microglia polarization. J. Control. Release **368**, 580–594 (2024). 10.1016/j.jconrel.2024.03.010 38467194 10.1016/j.jconrel.2024.03.010

[219] J. Zhang, C. Wang, X. Wu, Q. Shen, Y. Du, Nanozyme-based therapeutic strategies for rheumatoid arthritis. J. Control. Release **377**, 716–734 (2025). 10.1016/j.jconrel.2024.11.072 39617172 10.1016/j.jconrel.2024.11.072

[220] J. Yang, S. Xiao, J. Deng, Y. Li, H. Hu et al., Oxygen vacancy-engineered cerium oxide mediated by copper-platinum exhibit enhanced SOD/CAT-mimicking activities to regulate the microenvironment for osteoarthritis therapy. J. Nanobiotechnology **22**(1), 491 (2024). 10.1186/s12951-024-02678-z 39155382 10.1186/s12951-024-02678-z PMC11330606

[221] Q. Chen, X. Yang, Y. Yu, X. Duan, R. Ni et al., Biomimetic cerium-assisted supra-carbon dots assembly for reactive oxygen species-activated atherosclerosis theranostic. Small **21**(8), 2408980 (2025). 10.1002/smll.202408980 10.1002/smll.202408980 39777854

[222] Y. He, E. Peng, X. Ba, J. Wu, W. Deng et al., ROS responsive cerium oxide biomimetic nanoparticles alleviates calcium oxalate crystals induced kidney injury *via* suppressing oxidative stress and M1 macrophage polarization. Small **21**(3), 2405417 (2025). 10.1002/smll.202405417 10.1002/smll.202405417 39629501

[223] K. Zhang, M. Tu, W. Gao, X. Cai, F. Song et al., Hollow Prussian blue nanozymes drive neuroprotection against ischemic stroke *via* attenuating oxidative stress, counteracting inflammation, and suppressing cell apoptosis. Nano Lett. **19**(5), 2812–2823 (2019). 10.1021/acs.nanolett.8b04729 30908916 10.1021/acs.nanolett.8b04729

[224] M. Xu, D. Ran, J. Hu, J. Mao, D. Qiao et al., Multifunctional Prussian blue nanozymes alleviate atherosclerosis through inhibiting the inflammation feedback loop. J. Mater. Chem. B **13**(4), 1459–1473 (2025). 10.1039/D4TB01926A 39692245 10.1039/d4tb01926a

[225] C. Cho, H. Oh, J.S. Lee, L.-J. Kang, E.-J. Oh et al., Prussian blue nanozymes coated with Pluronic attenuate inflammatory osteoarthritis by blocking c-Jun N-terminal kinase phosphorylation. Biomaterials **297**, 122131 (2023). 10.1016/j.biomaterials.2023.122131 37119581 10.1016/j.biomaterials.2023.122131

[226] C. Chen, H. Wu, Q. Li, M. Liu, F. Yin et al., Manganese Prussian blue nanozymes with antioxidant capacity prevent acetaminophen-induced acute liver injury. Biomater. Sci. **11**(7), 2348–2358 (2023). 10.1039/D2BM01968J 36722889 10.1039/d2bm01968j

[227] S. Zhang, W. Ruan, J. Guan, Single-atom nanozymes for antibacterial applications. Food Chem. **456**, 140094 (2024). 10.1016/j.foodchem.2024.140094 38908326 10.1016/j.foodchem.2024.140094

[228] Y. Huang, J. Ren, X. Qu, Nanozymes: classification, catalytic mechanisms, activity regulation, and applications. Chem. Rev. **119**(6), 4357–4412 (2019). 10.1021/acs.chemrev.8b00672 30801188 10.1021/acs.chemrev.8b00672

[229] F. Wu, Y. Wang, Y. Li, L. Shi, L. Yuan et al., Single-atom Cu anchored on carbon nitride as a bifunctional glucose oxidase and peroxidase nanozyme for antibacterial therapy. ACS Nano **19**(11), 10816–10828 (2025). 10.1021/acsnano.4c12348 40087138 10.1021/acsnano.4c12348 PMC11948616

[230] Y. Zhang, C. Zhang, W. Qian, F. Lei, Z. Chen et al., Recent advances in MOF-based nanozymes: synthesis, activities, and bioapplications. Biosens. Bioelectron. **263**, 116593 (2024). 10.1016/j.bios.2024.116593 39059178 10.1016/j.bios.2024.116593

[231] L. Huang, D.-W. Sun, H. Pu, Photosensitized peroxidase mimicry at the hierarchical 0D/2D heterojunction-like quasi metal-organic framework interface for boosting biocatalytic disinfection. Small **18**(20), 2200178 (2022). 10.1002/smll.202200178 10.1002/smll.202200178 35436386

[232] B. Yang, H. Yao, J. Yang, C. Chen, Y. Guo et al., *In situ* synthesis of natural antioxidase mimics for catalytic anti-inflammatory treatments: rheumatoid arthritis as an example. J. Am. Chem. Soc. **144**(1), 314–330 (2022). 10.1021/jacs.1c09993 34881869 10.1021/jacs.1c09993

[233] B. Yang, H. Yao, J. Yang, C. Chen, J. Shi, Construction of a two-dimensional artificial antioxidase for nanocatalytic rheumatoid arthritis treatment. Nat. Commun. **13**(1), 1988 (2022). 10.1038/s41467-022-29735-1 35418125 10.1038/s41467-022-29735-1 PMC9008001

[234] M. Liao, Q. Cui, Y. Hu, J. Xing, D. Wu et al., Recent advances in the application of MXenes for neural tissue engineering and regeneration. Neural Regen. Res. **19**(2), 258–263 (2024). 10.4103/1673-5374.379037 37488875 10.4103/1673-5374.379037 PMC10503607

[235] H. Hu, H. Huang, L. Xia, X. Qian, W. Feng et al., Engineering vanadium carbide MXene as multienzyme mimetics for efficient *in vivo* ischemic stroke treatment. Chem. Eng. J. **440**, 135810 (2022). 10.1016/j.cej.2022.135810

[236] X. Sun, S. Luo, L. Zhang, Y. Miao, G. Yan, Photodynamic antibacterial activity of oxidase-like nanozyme based on long-lived room-temperature phosphorescent carbon dots. Food Chem. **434**, 137541 (2024). 10.1016/j.foodchem.2023.137541 37757701 10.1016/j.foodchem.2023.137541

[237] E.A. McHugh, A.V. Liopo, K. Mendoza, C.S. Robertson, G. Wu et al., Oxidized activated charcoal nanozymes: synthesis, and optimization for *in vitro* and *in vivo* bioactivity for traumatic brain injury. Adv. Mater. **36**(10), 2211239 (2024). 10.1002/adma.202211239 10.1002/adma.202211239 PMC10509328 36940058

[238] Q. Huang, Y. Yang, Y. Zhu, Q. Chen, T. Zhao et al., Oral metal-free melanin nanozymes for natural and durable targeted treatment of inflammatory bowel disease (IBD). Small **19**(19), e2207350 (2023). 10.1002/smll.202207350 36760016 10.1002/smll.202207350

[239] M. Cordani, J. Fernández-Lucas, A. Khosravi, E.N. Zare, P. Makvandi et al., Carbon-based nanozymes for cancer therapy and diagnosis: a review. Int. J. Biol. Macromol. **297**, 139704 (2025). 10.1016/j.ijbiomac.2025.139704 39793785 10.1016/j.ijbiomac.2025.139704

[240] A. Nair, R.H. Chandrashekhar, C.M. Day, S. Garg, Y. Nayak et al., Polymeric functionalization of mesoporous silica nanoparticles: Biomedical insights. Int. J. Pharm. **660**, 124314 (2024). 10.1016/j.ijpharm.2024.124314 38862066 10.1016/j.ijpharm.2024.124314

[241] Y. Yu, R. Tian, Y. Zhao, X. Qin, L. Hu et al., Self-assembled corrole/chitosan photothermal nanoparticles for accelerating infected diabetic wound healing. Adv. Healthc. Mater. **12**(16), e2201651 (2023). 10.1002/adhm.202201651 36168853 10.1002/adhm.202201651

[242] J. Ye, Y. Fan, Y. She, J. Shi, Y. Yang et al., Biomimetic self-propelled asymmetric nanomotors for cascade-targeted treatment of neurological inflammation. Adv. Sci. **11**(22), e2310211 (2024). 10.1002/advs.202310211 10.1002/advs.202310211 PMC11165487 38460166

[243] K.A. Choi, J.H. Kim, K. Ryu, N. Kaushik, Current nanomedicine for targeted vascular disease treatment: trends and perspectives. Int. J. Mol. Sci. **23**(20), 12397 (2022). 10.3390/ijms232012397 36293254 10.3390/ijms232012397 PMC9604340

[244] L. Li, S. Liu, J. Tan, L. Wei, D. Wu et al., Recent advance in treatment of atherosclerosis: key targets and plaque-positioned delivery strategies. J. Tissue Eng. **13**, 20417314221088508 (2022). 10.1177/20417314221088509 35356091 10.1177/20417314221088509 PMC8958685

[245] P. Dosta, I. Tamargo, V. Ramos, S. Kumar, D.W. Kang et al., Delivery of anti-microRNA-712 to inflamed endothelial cells using poly(β-amino ester) nanoparticles conjugated with VCAM-1 targeting peptide. Adv. Healthc. Mater. **10**(15), e2001894 (2021). 10.1002/adhm.202001894 33448151 10.1002/adhm.202001894 PMC8277885

[246] Q. Bai, Y. Xiao, H. Hong, X. Cao, L. Zhang et al., Scavenger receptor-targeted plaque delivery of microRNA-coated nanoparticles for alleviating atherosclerosis. Proc. Natl. Acad. Sci. U.S.A. **119**(39), e2201443119 (2022). 10.1073/pnas.2201443119 36122215 10.1073/pnas.2201443119 PMC9522431

[247] Y. Wang, Q. Zhou, L. Lu, J. Xu, G. Yang et al., Combining oxygen delivery and generation for targeted atherosclerosis therapy. J. Control. Release **380**, 1017–1030 (2025). 10.1016/j.jconrel.2025.02.053 39983924 10.1016/j.jconrel.2025.02.053

[248] T. Gui, L. Luo, B. Chhay, L. Zhong, Y. Wei et al., Superoxide dismutase-loaded porous polymersomes as highly efficient antioxidant nanoparticles targeting synovium for osteoarthritis therapy. Biomaterials **283**, 121437 (2022). 10.1016/j.biomaterials.2022.121437 35247635 10.1016/j.biomaterials.2022.121437 PMC8977249

[249] M.A. Beach, U. Nayanathara, Y. Gao et al., Polymeric nanoparticles for drug delivery. Chem. Rev. **124**(9), 5505–5616 (2024). 10.1021/acs.chemrev.3c00705 38626459 10.1021/acs.chemrev.3c00705 PMC11086401

[250] L. Guo, H. Yan, Q. Gong, W. Zheng, L. Zhong et al., *Glomerulus*-targeted ROS-responsive polymeric nanoparticles for effective membranous nephropathy therapy. ACS Appl. Mater. Interfaces **16**(27), 35447–35462 (2024). 10.1021/acsami.4c04345 38940537 10.1021/acsami.4c04345

[251] D. González-Restrepo, A. Zuluaga-Vélez, L.M. Orozco, J.C. Sepúlveda-Arias, Silk fibroin-based dressings with antibacterial and anti-inflammatory properties. Eur. J. Pharm. Sci. **195**, 106710 (2024). 10.1016/j.ejps.2024.106710 38281552 10.1016/j.ejps.2024.106710

[252] X. Zhang, Y. Liang, S. Huang, B. Guo, Chitosan-based self-healing hydrogel dressing for wound healing. Adv. Colloid Interface Sci. **332**, 103267 (2024). 10.1016/j.cis.2024.103267 39121832 10.1016/j.cis.2024.103267

[253] X. Ai, Y. Duan, Q. Zhang, D. Sun, R.H. Fang et al., Cartilage-targeting ultrasmall lipid-polymer hybrid nanoparticles for the prevention of cartilage degradation. Bioeng. Transl. Med. **6**(1), e10187 (2021). 10.1002/btm2.10187 33532587 10.1002/btm2.10187 PMC7823131

[254] R.M. Williams, J. Shah, E. Mercer, H.S. Tian, V. Thompson et al., Kidney-targeted redox scavenger therapy prevents cisplatin-induced acute kidney injury. Front. Pharmacol. **12**, 790913 (2022). 10.3389/fphar.2021.790913 35046813 10.3389/fphar.2021.790913 PMC8762298

[255] S. Meng, H. Wu, D. Xiao, S. Lan, A. Dong, Recent advances in bacterial cellulose-based antibacterial composites for infected wound therapy. Carbohydr. Polym. **316**, 121082 (2023). 10.1016/j.carbpol.2023.121082 37321715 10.1016/j.carbpol.2023.121082

[256] F.V. Ferreira, A.G. Souza, R. Ajdary, L.P. de Souza, J.H. Lopes et al., Nanocellulose-based porous materials: regulation and pathway to commercialization in regenerative medicine. Bioact. Mater. **29**, 151–176 (2023). 10.1016/j.bioactmat.2023.06.020 37502678 10.1016/j.bioactmat.2023.06.020 PMC10368849

[257] Y. Li, Y. Tian, W. Zheng, Y. Feng, R. Huang et al., Composites of bacterial cellulose and small molecule-decorated gold nanoparticles for treating gram-negative bacteria-infected wounds. Small **13**(27), 1700130 (2017). 10.1002/smll.201700130 10.1002/smll.201700130 28544761

[258] G. Tan, L. Wang, W. Pan, K. Chen, Polysaccharide electrospun nanofibers for wound healing applications. Int. J. Nanomed. **17**, 3913–3931 (2022). 10.2147/IJN.S371900 10.2147/IJN.S371900 PMC9464040 36097445

[259] S. Chen, R. Li, X. Li, J. Xie, Electrospinning: an enabling nanotechnology platform for drug delivery and regenerative medicine. Adv. Drug Deliv. Rev. **132**, 188–213 (2018). 10.1016/j.addr.2018.05.001 29729295 10.1016/j.addr.2018.05.001

[260] I. Dasgupta, A. Chatterjee, Recent advances in miRNA delivery systems. Methods Protoc. **4**(1), 10 (2021). 10.3390/mps4010010 33498244 10.3390/mps4010010 PMC7839010

[261] A. Vyawahare, R. Prakash, C. Jori, A. Ali, S.S. Raza et al., Caffeic acid modified nanomicelles inhibit articular cartilage deterioration and reduce disease severity in experimental inflammatory arthritis. ACS Nano **16**(11), 18579–18591 (2022). 10.1021/acsnano.2c07027 36222569 10.1021/acsnano.2c07027

[262] Y. Li, Q. Liang, L. Zhou, Y. Cao, J. Yang et al., An ROS-responsive artesunate prodrug nanosystem co-delivers dexamethasone for rheumatoid arthritis treatment through the HIF-1α/NF-κB cascade regulation of ROS scavenging and macrophage repolarization. Acta Biomater. **152**, 406–424 (2022). 10.1016/j.actbio.2022.08.054 36055613 10.1016/j.actbio.2022.08.054

[263] P. Khare, S.X. Edgecomb, C.M. Hamadani, E.E.L. Tanner, D.S. Manickam, Lipid nanoparticle-mediated drug delivery to the brain. Adv. Drug Deliv. Rev. **197**, 114861 (2023). 10.1016/j.addr.2023.114861 37150326 10.1016/j.addr.2023.114861

[264] H.M. Eid, A.A. Ali, A.M. Abdelhaleem Ali, E.M. Eissa, R.M. Hassan et al., Potential use of tailored citicoline chitosan-coated liposomes for effective wound healing in diabetic rat model. Int. J. Nanomed. **17**, 555–575 (2022). 10.2147/IJN.S342504 10.2147/IJN.S342504 PMC8828492 35153481

[265] M.R. Arabestani, A. Bigham, F. Kamarehei, M. Dini, F. Gorjikhah et al., Solid lipid nanoparticles and their application in the treatment of bacterial infectious diseases. Biomed. Pharmacother. **174**, 116433 (2024). 10.1016/j.biopha.2024.116433 38508079 10.1016/j.biopha.2024.116433

[266] E. Ortega Martínez, M.E. Morales Hernández, J. Castillo-González, E. González-Rey, M.A. Ruiz Martínez, Dopamine-loaded chitosan-coated solid lipid nanoparticles as a promise nanocarriers to the CNS. Neuropharmacology **249**, 109871 (2024). 10.1016/j.neuropharm.2024.109871 38412889 10.1016/j.neuropharm.2024.109871

[267] M. Abudurexiti, J. Xue, X. Li, X. Zhang, Y. Qiu et al., Curcumin/TGF-β1 siRNA loaded solid lipid nanoparticles alleviate cerebral injury after intracerebral hemorrhage by transnasal brain targeting. Colloids Surf. B Biointerfaces **237**, 113857 (2024). 10.1016/j.colsurfb.2024.113857 38552289 10.1016/j.colsurfb.2024.113857

[268] M. Mohammed, U.H. Ibrahim, A. Aljoundi, C.A. Omolo, N. Devnarain et al., Enzyme-responsive biomimetic solid lipid nanoparticles for antibiotic delivery against hyaluronidase-secreting bacteria. Int. J. Pharm. **640**, 122967 (2023). 10.1016/j.ijpharm.2023.122967 37084831 10.1016/j.ijpharm.2023.122967

[269] H.-C. Wang, W. Yang, L. Xu, Y.-H. Han, Y. Lin et al., BV2 membrane-coated PEGylated-liposomes delivered hFGF21 to cortical and hippocampal microglia for Alzheimer’s disease therapy. Adv. Healthc. Mater. **13**(19), 2400125 (2024). 10.1002/adhm.202400125 10.1002/adhm.202400125 38513154

[270] J. Mondal, S. Pillarisetti, V. Junnuthula, M. Saha, S.R. Hwang et al., Hybrid exosomes, exosome-like nanovesicles and engineered exosomes for therapeutic applications. J. Control. Release **353**, 1127–1149 (2023). 10.1016/j.jconrel.2022.12.027 36528193 10.1016/j.jconrel.2022.12.027

[271] R. Yang, Y. Liao, L. Wang, P. He, Y. Hu et al., Exosomes derived from M2b macrophages attenuate DSS-induced colitis. Front. Immunol. **10**, 2346 (2019). 10.3389/fimmu.2019.02346 31749791 10.3389/fimmu.2019.02346 PMC6843072

[272] G. Guo, Z. Tan, Y. Liu, F. Shi, J. She, The therapeutic potential of stem cell-derived exosomes in the ulcerative colitis and colorectal cancer. Stem Cell Res. Ther. **13**(1), 138 (2022). 10.1186/s13287-022-02811-5 35365226 10.1186/s13287-022-02811-5 PMC8973885

[273] E. Jin, Y. Yang, S. Cong, D. Chen, R. Chen et al., Lemon-derived nanoparticle-functionalized hydrogels regulate macrophage reprogramming to promote diabetic wound healing. J. Nanobiotechnology **23**(1), 68 (2025). 10.1186/s12951-025-03138-y 39891270 10.1186/s12951-025-03138-y PMC11783766

[274] B. Zhao, H. Lin, X. Jiang, W. Li, Y. Gao et al., Exosome-like nanoparticles derived from fruits, vegetables, and herbs: innovative strategies of therapeutic and drug delivery. Theranostics **14**(12), 4598–4621 (2024). 10.7150/thno.97096 39239509 10.7150/thno.97096 PMC11373634

[275] Z. Tian, H. Ning, X. Wang, Y. Wang, T. Han et al., Endothelial autophagy promotes atheroprotective communication between endothelial and smooth muscle cells *via* exosome-mediated delivery of miR-204-5p. Arterioscler. Thromb. Vasc. Biol. **44**(8), 1813–1832 (2024). 10.1161/ATVBAHA.123.319993 38957984 10.1161/ATVBAHA.123.319993

